# Tricarbocyanine *N*-triazoles: the scaffold-of-choice for long-term near-infrared imaging of immune cells *in vivo*†
†Electronic supplementary information (ESI) available: Additional details for theoretical calculations, chemical synthesis and characterisation of all probes; supplementary figures and experimental procedures for all assays performed with murine and human CD4^+^ T cells. See DOI: 10.1039/c8sc00900g


**DOI:** 10.1039/c8sc00900g

**Published:** 2018-08-08

**Authors:** Richard J. Mellanby, Jamie I. Scott, Iris Mair, Antonio Fernandez, Louise Saul, Jochen Arlt, Monica Moral, Marc Vendrell

**Affiliations:** a Medical Research Council Centre for Inflammation Research , The University of Edinburgh , 47 Little France Crescent , EH16 4TJ Edinburgh , UK . Email: marc.vendrell@ed.ac.uk; b Royal (Dick) School of Veterinary Studies , The Roslin Institute , Division of Veterinary Clinical Studies , The University of Edinburgh , Hospital for Small Animals , Easter Bush Veterinary Centre , EH25 9RG Roslin , UK . Email: richard.mellanby@ed.ac.uk; c School of Physics and Astronomy , The University of Edinburgh , James Clerk Maxwell Building, Peter Guthrie Tait Road , EH9 3FD Edinburgh , UK; d Renewable Energy Research Institute , University of Castilla-La Mancha , 02071 Albacete , Spain

## Abstract

Herein tricarbocyanine *N*-triazoles are first described as a rationally-designed near-infrared (NIR) structure overcoming the brightness and photostability limitations of tricarbocyanines for long-term *in vivo* imaging. The straightforward synthetic approach and the wide availability of alkynes makes this strategy a versatile methodology for the preparation of highly stable *N*-substituted tricarbocyanines. Furthermore, we validated **CIR38M** as a non-transferable marker to monitor the fate of therapeutic T cells non-invasively *in vivo*, showing enhanced performance over conventional NIR fluorophores (*i.e.***DiR**, IR800CW and indocyanine green) as well as compatibility with human cells for translational studies. **CIR38M** is able to track over time smaller numbers of T cells than current NIR agents, and to visualise antigen-driven accumulation of immune cells at specific sites *in vivo*. This chemical technology will improve longitudinal imaging studies to assess the efficacy of cell-based immunotherapies in preclinical models and in human samples.

## Abstract 2

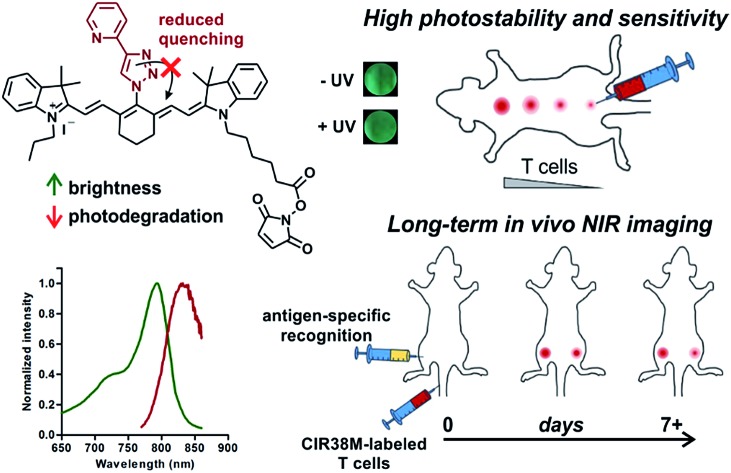
Tricarbocyanine *N*-triazoles are first described as rationally-designed structures to overcome the limitations of NIR dyes for long-term *in vivo* imaging.

## Introduction

**Fig. 1:**
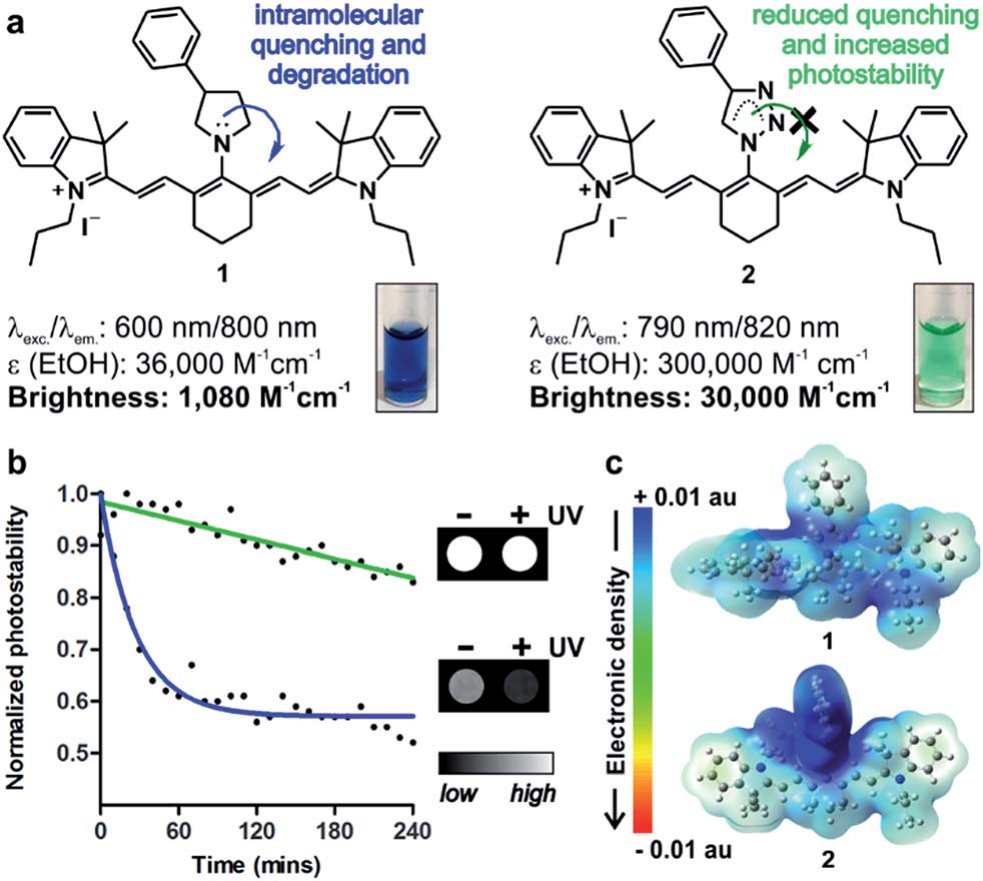
(a) Chemical structures and properties of isosteric tricarbocyanine *N*-amines (**1**) and *N*-triazoles (**2**) in EtOH. (Inset) Absorbance pictograms of compounds **1** and **2** (10 μM). (b) Photostability analysis of compounds **1** (blue) and **2** (green) (both at 50 μM in PBS) under continuous light irradiation. Solid lines correspond to one phase exponential decay regressions for both sets of values. (Inset) NIR fluorescence pictograms of compounds **1** and **2** (10 μM in PBS) before and after UV light irradiation. (c) Pseudo-coloured electronic distribution maps for compounds **1** (top) and **2** (bottom) in their fundamental state.

*In vivo* optical imaging has revolutionised our ability to visualise biological processes with high resolution in intact organisms. Most *in vivo* imaging fluorophores rely on near-infrared (NIR) chemical scaffolds as they allow deep penetration with minimal photodamage and low tissue autofluorescence.^1^,^2^ Many NIR fluorophores have been described for bioimaging applications, ranging from analyte detection to image-guided surgery.^3^–^10^ Choi, Henary and Frangioni among others recently exploited the diversification of NIR cyanine fluorophores for structure-inherent targeting of different tissues.^11^–^13^ Zwitterionic heptamethine cyanine analogues have also been recently described as NIR agents with enhanced capabilities for cell, tissue and *in vivo* imaging.^14^–^16^ Among these structures, the sulfonated heptamethine cyanine dye Indocyanine Green (**ICG**) is the only clinically-approved NIR dye for studies in humans.^17^ The structurally-related IR800CW fluorophores have recently entered clinical trials as biomarker-labelling molecules for fluorescence-assisted surgery.^18^ In both **ICG** and IR800CW, the potential aggregation of the heptamethine cyanine scaffold is minimised by the incorporation of negatively-charged groups (*i.e.* sulfonates); however, these preclude cell uptake and impede long-term tracking of small populations of cells *in vivo*. As an alternative, tricarbocyanine *N*-amines have been reported as cell-permeable NIR fluorophores, and they can be prepared *via* nucleophilic substitution of the **IR780** tricarbocyanine core with amines (**1**, Fig. 1).^19^–^22^ Their straightforward chemistry has enabled their adaptation to diversity-oriented studies,^23^–^26^ but most tricarbocyanines *N*-amines show low quantum yields and rapid photodegradation, which compromise their application for long-term NIR fluorescence imaging. We addressed these shortcomings by rationally designing tricarbocyanine *N*-triazoles as a new family of bright, photostable and cell-permeable NIR fluorophores containing neutral triazole groups. We envisaged that the electron delocalization in the triazole ring would reduce the electron density of *N*-substituted groups, thus minimizing intramolecular quenching and photodegradation of the tricarbocyanine core. To date, tricarbocyanine *N*-triazoles had not been isolated because of the lack of synthetic approaches that were compatible with the relatively unstable intermediate tricarbocyanine azide. Herein we describe a synthetic approach for the isolation of tricarbocyanine *N*-triazoles in reasonable yields and high purities.

## Results and discussion

### Design and synthesis of tricarbocyanine *N*-triazoles

**Fig. 2:**
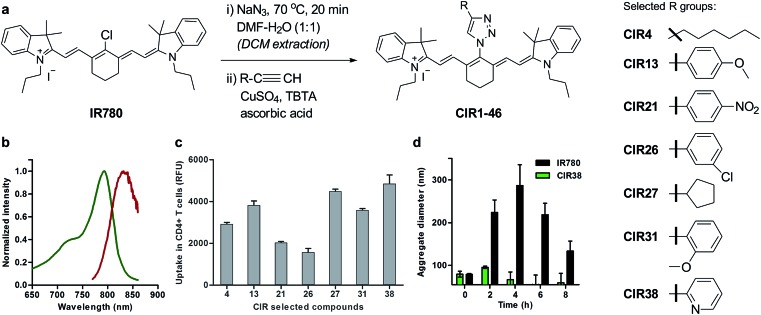
Chemical synthesis of CIR fluorophores. (a) Structures of selected CIR fluorophores (see Table S2†). (b) Absorbance (green) and emission (red) spectra of **CIR38** as a representative CIR fluorophore. (c) Fluorescence NIR intensity of selected CIR fluorophores upon incubation with CD4^+^ T cells (5 × 10^5^ cells, 10 μM PBS, *λ*_exc._: 790 nm; *λ*_em._: 820 nm). Values are represented as means ± s.e.m (*n* = 3). (d) Time-course analysis of the mean aggregate size of **IR780** and **CIR38** in aqueous media (100 μM PBS) determined by dynamic light scattering at r.t. for up to 8 h. Values are represented as means ± s.e.m (*n* = 3).

Nucleophilic substitution of **IR780** with sodium azide in DMF : H_2_O (1 : 1) followed by rapid DCM extraction and 1,3-Huisgen copper-catalysed cycloaddition rendered tricarbocyanine *N*-triazoles (**2**, Fig. 1 and 2). Triazole derivatives display superior spectral properties when compared to their isosteric tricarbocyanine *N*-amines, despite only differing in two nitrogen atoms being replaced by methylene groups. In addition to red-shifted excitation and emission wavelengths, tricarbocyanine *N*-triazoles exhibit higher extinction coefficients and quantum yields (*i.e.* 3% for **1**, 10% for **2**) with 30-fold increase in brightness and remarkably enhanced photostability (Fig. 1). To analyse the differential behaviour of isosteric amine (**1**) and triazole (**2**) fluorophores, we determined their electron density distributions and transitions with Gaussian 09 (Fig. 1 and S1†).^27^

These studies highlight the phenyltriazole group as the most electron-deficient region within **2** (Fig. 1c), minimising any intramolecular quenching. Electronic transitions for both tricarbocyanines **1** and **2** involve charge transfer processes from the heptamethine core to the phenylpyrrolidine in compound **1** (*i.e.* α-HOMO to α-LUMO and β-HOMO to β-SOMO) or to the phenyltriazole in compound **2** (*i.e.* β-HOMO to β-SOMO) (Table S1†). However, the bridgehead nitrogen atom of the pyrrolidine ring in compound **1** shows significantly higher contribution to α-LUMO (6%) and β-SOMO (6%) than the corresponding nitrogen atom of the triazole ring in compound **2** (2% to its β-SOMO) (Fig. S1†). This observation confirms our hypothesis that the reduced electron density at the bridgehead nitrogen atom in tricarbocyanine *N*-triazoles results in enhanced photostability upon photoexcitation. Altogether, the properties of the tricarbocyanine *N*-triazole scaffold as a bright, cell-permeable and photostable NIR structure encouraged us to explore its potential to produce new fluorophores able to track small populations of cells *in vivo* for longer periods of time than currently available NIR dyes.

### Chemical optimisation of a photostable NIR fluorophore for labelling CD4^+^ T cells

Cellular immunotherapies represent promising strategies for treating disorders driven by malfunctioning immune responses, including cancer, chronic infections and autoimmune diseases.^28^–^34^ Among these, T cell immunotherapies have shown great potential, in both experimental models and human patients.^35^–^39^ For instance, in cancer immunotherapy, tumour-responsive T cells are isolated from the peripheral blood of patients, expanded *ex vivo* and then transferred back to elicit anti-tumour immune responses. One important obstacle in the clinical translation of T cell immunotherapies is the lack of chemical agents to track post-transferred therapeutic cells *in vivo*.^40^

Longitudinal imaging allows researchers to evaluate whether T cells reach and accumulate at the site of disease, particularly shortly after the transfusion of the cells, when adverse events may occur. Despite the utility of magnetic resonance imaging (MRI) and radionuclide-based imaging for cell tracking,^41^–^43^ their respective limited sensitivity and safety concerns hamper their utility to detect small numbers of therapeutic T cells (*e.g.* <10 000 cells) *in vivo* and at multiple time points. Using the above mentioned synthetic approach, we prepared a collection of ‘click’ infrared (CIR) fluorophores by modifying **IR780** with 46 structurally-diverse alkynes (Fig. 2a). CIR fluorophores were isolated by semi-preparative HPLC in very high purities (>95%), and, unlike amino-derivatised tricarbocyanines,^44^ all CIR fluorophores showed excitation and emission wavelengths in the NIR window (*λ*_exc._ ∼ 780–800 nm, *λ*_em._ ∼ 805–840 nm) (Fig. 2b and Tables S2 and S3†). Given the broad availability of alkyne building blocks and versatility of the synthetic approach, this route might become the methodology-of-choice to produce bright and photostable NIR dyes *via* functionalisation of tricarbocyanines with *N*-substituted groups.

**Fig. 3:**
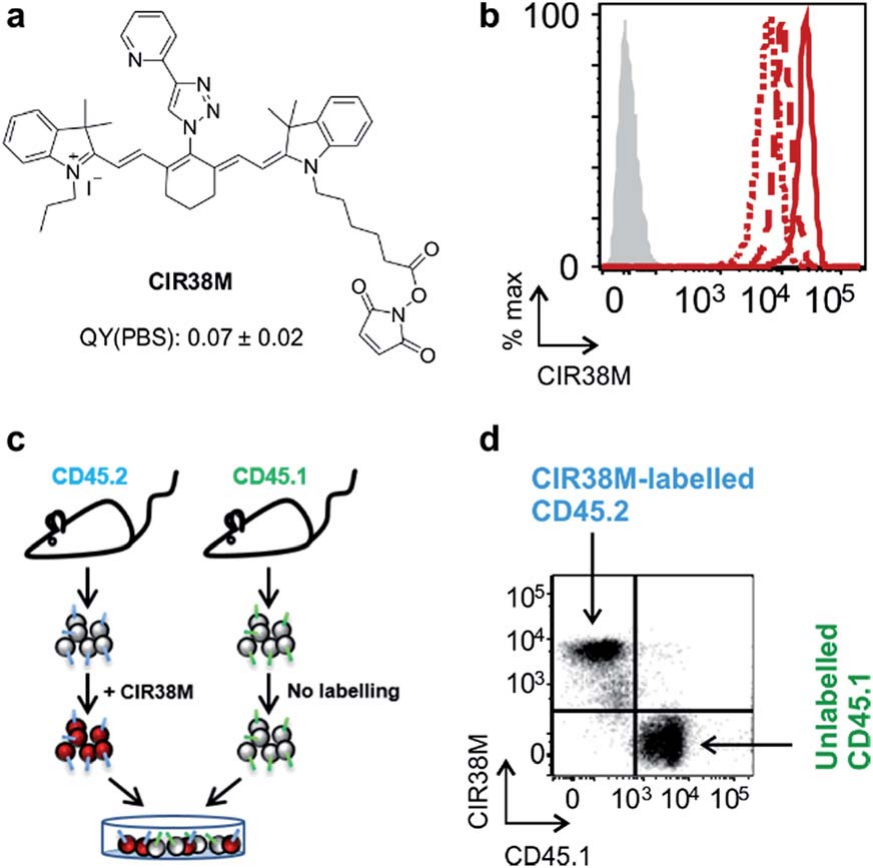
**CIR38M** brightly labels CD4^+^ T cells with no leaking to neighbouring cells. (a) Chemical structure of **CIR38M** and fluorescence quantum yield in PBS (relative to **ICG**). (b) Murine CD4^+^ T cells were labelled with **CIR38M** (10 μM, 2 min) and cultured on anti-CD3 and anti-CD28 coated plates. NIR fluorescence emission was measured by flow cytometry on day 0 (solid), day 1 (dashed) and day 3 (dotted), compared to unlabelled cells (shaded). (c, d) CD4^+^ T cells expressing the congenic marker CD45.2 were labelled with **CIR38M** and co-cultured with equal numbers of unlabelled CD4^+^ T cells expressing the congenic marker CD45.1. After 3 days of *in vitro* culture, fluorescence emission of CD45.1^+^ and CD45.2^+^ cells were assessed by flow cytometry.

Next, we evaluated the fluorescence emission of the 46 CIR fluorophores upon incubation with murine T cells, and **CIR38** showed the brightest fluorescence emission (Fig. 2c). Since the derivatisation of fluorescent sulfonamides with 1,2,3-triazole groups has been recently reported to produce water-soluble carbonic anhydrase inhibitors with optimal properties for *in vivo* studies,^45^ we envisaged that the neutral uncharged character of **CIR38** would also enhance the solubility of the tricarbocyanine core and form less non-fluorescent aggregates in aqueous media. We compared water solutions of **CIR38** and the generic heptamethine structure **IR780** by dynamic light scattering, and observed that **CIR38** formed less insoluble aggregates in water (Fig. 2d), highlighting the pyridinyl-triazole moiety as an effective chemical group to enhance the solubility of heptamethine dyes. Furthermore, **CIR38** showed excellent photostability, retaining full chemical integrity even after 12 h of continuous UV light irradiation (Fig. S2†). Based on these results, we synthesized both maleimide (**CIR38M**) and succinimidyl ester (**CIR38SE**) analogues to increase its intracellular retention (Fig. 3a and Scheme S1†). The derivatisation of **CIR38** with reactive groups did not affect the NIR spectral properties nor the uptake in murine CD4^+^ T cells (Fig. S3†). We selected **CIR38M** as a fluorophore with similar spectral properties to NIR commercially available dyes (Table S4†) and optimal features for intracellular labelling of CD4^+^ T cells under physiological conditions.

### *In vitro* characterisation of **CIR38M** for murine T cell imaging

First, we examined the staining of CD4^+^ T cells following rapid 2 min incubation with different concentrations of **CIR38M**, and observed that the incubation of CD4^+^ T cells with 10 μM **CIR38M** provided very bright fluorescence signals, close to saturation levels (Fig. S4†). Next, we assessed the intensity of **CIR38M** after several cell divisions by stimulating CD4^+^ T cells with plate-bound anti-CD3/CD28 antibodies and measuring the NIR fluorescence emission for several days. As shown in Fig. S5,† a smaller decline in fluorescence emission was observed after 3 days of *in vitro* stimulation in **CIR38M**-labelled CD4^+^ T cells when compared to CFSE, a standard fluorescent marker of cell proliferation. **CIR38M** therefore demonstrated the utility to label T cells for multiple rounds of proliferation without a major loss of fluorescence intensity.

We then analysed the stability of **CIR38M** labelling and evaluated whether the probe leaked to neighbouring cells, which would lead to the misinterpretation of *in vivo* imaging data. We employed **CIR38M** to label CD4^+^ T cells expressing the CD45.2 cell marker (*i.e.* CD45.2^+^ CD4^+^ T cells), and co-cultured them with the same number of unlabelled CD4^+^ T cells expressing the CD45.1 cell marker (*i.e.* CD45.1^+^ CD4^+^ T cells) (Fig. 3c). The expression of two different CD45 congenic markers allowed us to distinguish between **CIR38M**-labelled and unlabelled cells in the co-culture. Flow cytometry analysis after 3 days under stimulatory conditions confirmed the intracellular retention of **CIR38M**, as labelled cells expressed the CD45.2 marker but not the CD45.1 marker (Fig. 3d). The fluorescence characterisation of **CIR38M**-labelled CD4^+^ T cell lysates also showed that **CIR38M** was retained inside cells by forming covalent bonds with intracellular proteins (Fig. S6†), preventing leakage to neighbouring cells. In addition, we did not observe significant differences in the viability of CD4^+^ T cells after **CIR38M** treatment nor reduction in proliferation after culture for 3 days in stimulatory conditions (Fig. S7a and b†). The non-invasive character of **CIR38M** was also corroborated by measuring the production of key pro-inflammatory cytokines (*e.g.* TNF-α, GM-CSF) of stimulated labelled CD4^+^ T cells, which remained unaltered after treatment with **CIR38M** (Fig. S7c and d†).

### Fluorescence microscopy of murine CD4^+^ T cells

**Fig. 4:**
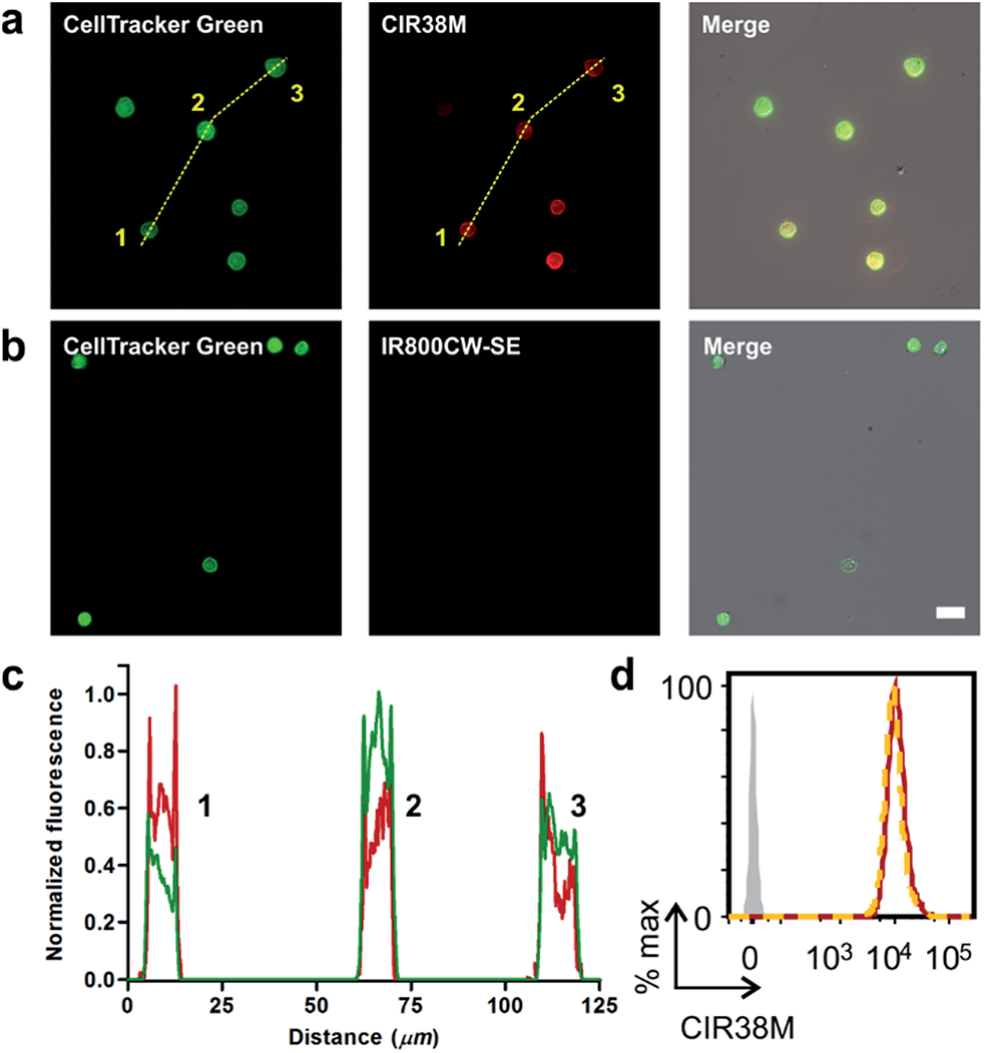
Fluorescence cell imaging of **CIR38M**-labelled T cells. (a) Brightfield, fluorescence and merged microscope images of CD4^+^ T cells after labelling with **CIR38M** and CellTracker Green. (b) Brightfield, fluorescence and merged microscope images of CD4^+^ T cells after labelling with **IR800CW-SE** and CellTracker Green. Scale bar: 10 μm. (c) Plot profile analysis of the fluorescence intracellular staining of CellTracker Green (green) and **CIR38M** (red) shown images in (a) (dashed yellow lines). (d) Intracellular staining of **CIR38M** confirmed by comparison of **CIR38M**-labelled CD4^+^ T cells (red) to **CIR38M**-labelled CD4^+^ T cells treated with 0.1% trypan blue (orange).

Given the features of **CIR38M** as a NIR fluorophore for non-invasive labelling of CD4^+^ T cells, we performed *in vitro* fluorescence microscopy experiments to compare its imaging capabilities to other fluorophores emitting in the far NIR region (*i.e.* 800–900 nm). We compared the fluorescence staining of **CIR38M** to the commercially available **IR800CW-SE**, a gold standard in NIR fluorescence labelling,^46^,^47^ by treating CD4^+^ T cells with both fluorophores under the same conditions and visualising them under a fluorescence microscope. Counter-staining with the green fluorophore CellTracker Green was used to confirm CD4^+^ T cell labelling. As shown in Fig. 4a and b, the lack of charges and enhanced permeability of **CIR38M** led to much brighter staining of CD4^+^ T cells than **IR800CW-SE**. Direct comparison of **CIR38M** to **IR780M**, the maleimide analogue of **IR780** (Scheme S2†), showed similar intensity but more photostable NIR signals with the former, highlighting the importance of the *N*-triazole group within the **CIR38M** structure (Fig. S8†). Notably, the strong fluorescence emission of **CIR38M** enabled imaging of labelled CD4^+^ T cells using both high and low-power excitation sources (*i.e.* Ti-sapphire laser and mercury lamps, respectively) (Fig. S9†) as well as low magnification objectives (Fig. S10†), expanding the scope of applications for CIR fluorophores to various optical imaging modalities. High-magnification images were analysed by plot profiling (Fig. 4c) and confirmed that **CIR38M**, like CellTracker Green, localised mainly in the cytosol of CD4^+^ T cells with preferential accumulation in mitochondria compared to other organelles (*e.g.* lysosomes) (Fig. S11†). Flow cytometry analysis also corroborated over 12-fold higher CD4^+^ T cell staining of **CIR38M** when compared to **IR800CW-SE** (Fig. S12†), and its intracellular retention was confirmed by lack of fluorescence quenching in an exclusion assay with the impermeant dye trypan blue^48^ (Fig. 4d).

Altogether, these results validate the applicability of **CIR38M** as a bright and non-toxic NIR agent to track the recruitment, expansion and mobility of CD4^+^ T cells without affecting their physiology and with enhanced capabilities over commercially available NIR dyes (*e.g.***IR800CW-SE**).

### *In vivo* long-term tracking of labelled T cells

**Fig. 5:**
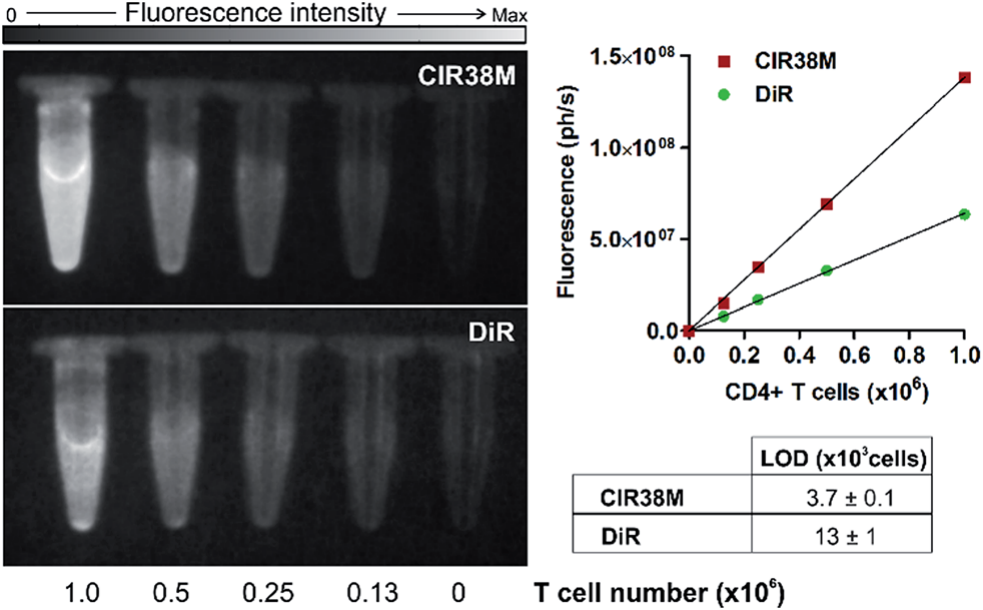
Determination of the limit of detection in suspensions of **CIR38M-**labelled and **DiR**-labelled (both at 10 μM) CD4^+^ T cells by NIR fluorescence imaging. Values shown as means ± s.e.m. Images acquired in PhotonImager™ (*λ*_exc._ ∼ 760 nm; *λ*_em._ ∼ 800–900 nm). Max: 2 × 10^8^ photons per s.

**Fig. 6:**
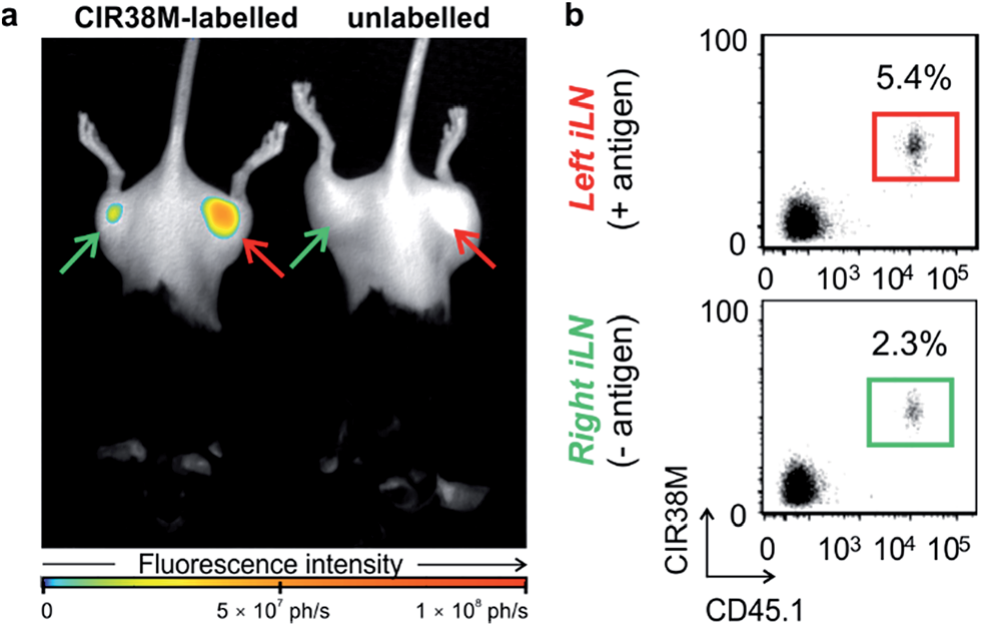
Site-specific accumulation of **CIR38M**-labelled cells in a model of T cell activation. (a) Whole-body fluorescence images (*λ*_exc._ ∼ 760 nm; *λ*_em._ ∼ 800–900 nm) of C57BL/6 mice (3 per group) 2 days after the tail vein i.v. injection of **CIR38M**-labelled (left) or unlabelled CD45.1^+^ CD4^+^ OT-II T cells (right). Right hind legs (green arrows) were injected with PBS emulsified in CFA whereas left hind legs (red arrows) were injected with 10 μg pOVA emulsified in CFA. (b) Flow cytometric *ex vivo* analysis of inguinal lymph nodes (iLNs) from both left and right hind limbs of mice that had been injected with **CIR38M**-labelled CD4^+^ T cells. Percentages indicate the proportion of CD45.1^+^ donor cells within the CD4^+^ T cell compartment.

Next, we examined the properties of **CIR38M** for tracking CD4^+^ T cells *in vivo* using whole-body NIR fluorescence imaging. To assess the capabilities of **CIR38M** for detecting small populations of therapeutic T cells, CD4^+^ T cells were labelled with **CIR38M** and sequential dilutions were prepared before being directly imaged using whole-body imaging acquisition settings. The limit of detection of **CIR38M** was around 4000 cells, 3-fold lower than the conventional NIR cell tracer **DiR**,^49^,^50^ highlighting the utility of **CIR38M** for monitoring small numbers of T cells that could not be detected by other methods (Fig. 5). We then applied **CIR38M** in a model of T cell activation, whereby an antigen was injected subcutaneously together with complete Freund's adjuvant (CFA), to monitor the dynamics of T cell activation *in vivo*. The co-administration of autoantigens and CFA is one of the most used approaches to induce experimental disease and to examine T cell activation *in vivo*. Numerous models have been described using this approach.^51^–^55^ With these experiments, we examined whether: (1) **CIR38M** could be used to image the antigen-specific accumulation of T cells *in vivo*, and (2) **CIR38M** could be used to track T cells longitudinally during the whole process of *in vivo* activation. To address the first question, we transferred **CIR38M**-labelled CD45.1^+^ CD4^+^ OT-II T cells, which express a T cell receptor that responds to ovalbumin peptide (pOVA), into CD45.2^+^ CD45.1^–^ host mice. Host mice were administered a subcutaneous injection of CFA and pOVA antigen on the left hind leg to recruit the labelled OT-II T cells into the antigen-containing site, and CFA and PBS on the right hind leg as a negative control. We acquired whole-body fluorescence images of the mice 48 h post-injection and observed that **CIR38M**-labelled CD4^+^ T cells preferentially accumulated in the lymph nodes close to where the pOVA antigen had been injected (Fig. 6a). The CD45.1^+^ expression on OT-II cells allowed the transferred cells to be identified *ex vivo* by flow cytometry analysis, and we confirmed over 2-fold higher **CIR38M**-labelled cells in the lymph nodes draining the side immunised with CFA and pOVA when compared to the lymph nodes draining the side immunised with CFA and PBS (Fig. 6b).

*Ex vivo* analysis of the lymph nodes confirmed that **CIR38M** was only present in CD45.1^+^ donor cells and had not transferred onto host CD45.1^–^ cells, confirming that no leakage of **CIR38M** to other subpopulations of T cells occurred *in vivo*. These results corroborate that the fluorescence emission of **CIR38M** directly correlated to the antigen-specific accumulation of CD4^+^ T cells in mice.

**Fig. 7:**
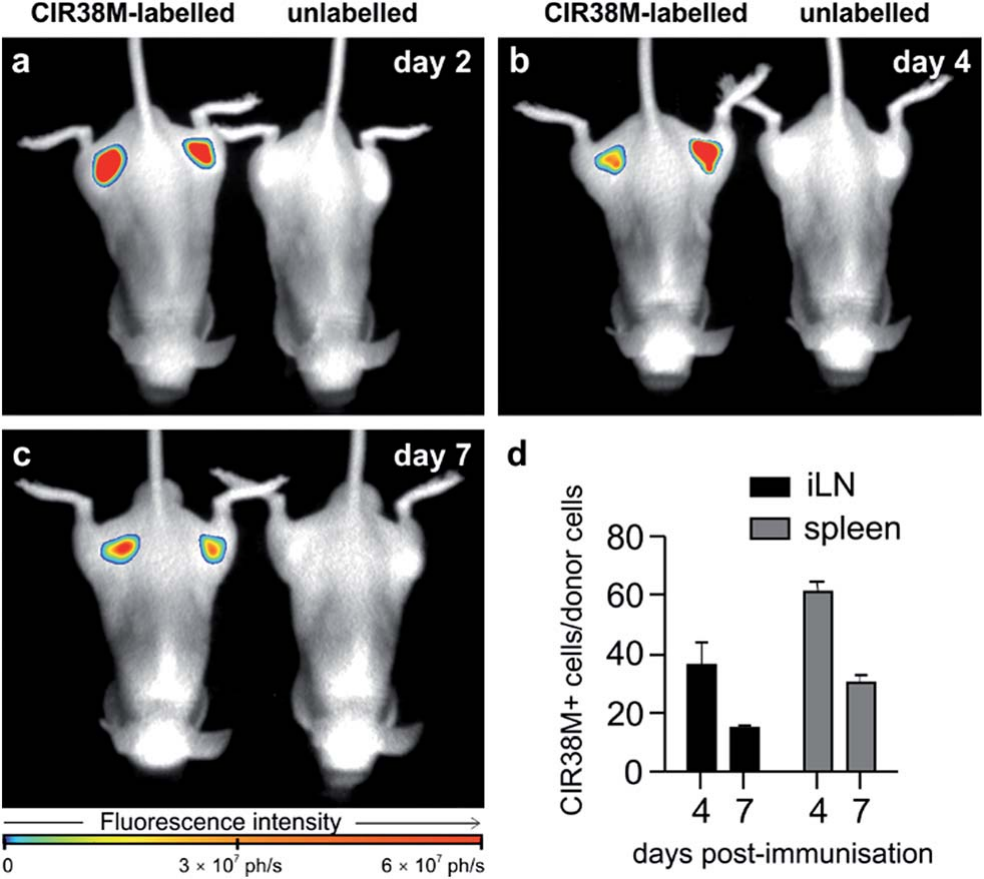
Long-term longitudinal tracking of **CIR38M**-labelled CD4^+^ T cells *in vivo*. C57BL/6 (B6.Cg-Tyr^c-2J^ Hr^hr^/J) mice were injected i.v. (tail vein) with **CIR38M**-labelled or unlabelled pOVA-reactive CD45.1^+^ CD4^+^ OT-II T cells and immunised by injection of pOVA and CFA into each limb. Whole-body *in vivo* fluorescence images (*λ*_exc._ ∼ 760 nm; *λ*_em._ ∼ 800–900 nm) of representative mice (3 per group) injected with **CIR38M**-labelled or unlabelled T cells were acquired on (a) day 2 (b) day 4 and (c) day 7, prior to organ harvest and flow cytometry. (d) Proportion of donor cells retaining NIR fluorescence in the inguinal lymph nodes (iLN) draining the immunisation site and the spleen on days 4 and 7. Values as means ± s.e.m (*n* = 3 per group).

To address the second question, we examined **CIR38M** for long-term imaging of T cell activation *in vivo*. **CIR38M**-labelled CD45.1^+^ CD4^+^ OT-II T cells were transferred into C57BL/6 hairless mice, followed by immunisation with CFA and pOVA at both hind limbs. Whole-body fluorescence images were acquired at 2, 4 and 7 days after immunisation. As shown in Fig. 7a–c, **CIR38M**-labelled T cells were clearly visible in the draining lymph nodes for the entirety of the *in vivo* activation process. Despite the highly proliferative environment which occurs when T cells interact with antigen in draining lymph nodes, quantitative analysis confirmed that ∼40% of the transferred T cells still retained **CIR38M** at day 4 and over 15% at day 7 (Fig. 7d). **CIR38M**-stained T cells were detected in the spleen *ex vivo*, but were difficult to image in whole bodies due to its deep location *in vivo*. The lack of NIR fluorescence emission in other immune cells, most importantly CD11b^+^ phagocytic myeloid cells, corroborated the applicability of **CIR38M** to faithfully identify the originally labelled T cells (Fig. S13†). Furthermore, we confirmed the presence of **CIR38M**-labelled cells by *ex vivo* analysis using flow cytometry and NIR tissue imaging (Fig. S14†).

Furthermore, we compared the sensitivity of **CIR38M** and **DiR** for detecting post-transferred cells in mice. We injected small numbers of **CIR38M** or **DiR**-labelled Tg4 CD4^+^ T cells, which express a transgenic T cell receptor that responds to myelin basic protein (MBP), into mice that were immunised with MBP peptide, and monitored their accumulation in antigen-treated lymph nodes by whole-body imaging. Brighter signals were detected for **CIR38M**-labelled cells (Fig. S15†), confirming its suitability for NIR imaging of activated CD4^+^ T cells even when low numbers of cells are recruited. Finally, to assess any potential effects of **CIR38M**-labelled cells on T cell physiology upon *in vivo* activation, we compared the recruitment of T cells to the lymph nodes in mice that had either received labelled or unlabelled CD45.1^+^ CD4^+^ OT-II T cells. We found no significant differences between the absolute numbers nor the proportions of CD45.1^+^ CD4^+^ OT-II T cells at any of the time points, which confirms the non-perturbative character of **CIR38M** for non-invasive *in vivo* imaging of cellular localisation and proliferation (Fig. S16†). We also examined any potential systemic toxicity derived from the injection of **CIR38M**-labelled CD4^+^ T cells. We analysed the biochemical profile of serum from mice that had been immunised with pOVA and CFA and injected the same amounts of either **CIR38M**-labelled or non-labelled CD45.1^+^ CD4^+^ OT-II T cells. Serum biochemistry was performed from mice that had been administered either **CIR38M**-labelled or unlabelled cells, and no significant differences were observed between the two groups in any of the analytes measured (Table S5†). Furthermore, H&E staining of formalin-fixed livers and kidney sections revealed no tissue pathology after administration of **CIR38M**-labelled cells (Fig. S17†).

### *Ex vivo* staining of human CD4^+^ T cells

**Fig. 8:**
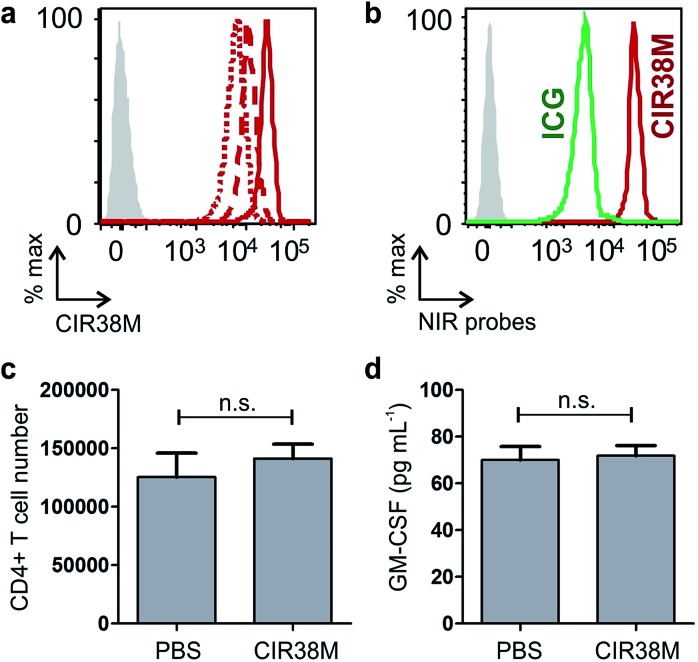
*Ex vivo* staining of human T cells with **CIR38M**. (a) Flow cytometric analysis of human CD4^+^ T cells from peripheral blood after labelling with **CIR38M** and culture with equal amounts of antigen-presenting cells and 2 μg mL^–1^ soluble anti-CD3. NIR fluorescence on day 0 (solid), day 1 (dashed) and day 3 (dotted) in comparison to unlabelled cells (shade). (b) Analysis of human CD4^+^ T cells labelled with **CIR38M** (red) or **ICG** (green) under the same conditions. (c) Equal numbers of human CD4^+^ T cells were incubated with **CIR38M** or PBS, incubated overnight at 37 °C and viable cells were counted 24 h later. Values represented as means ± s.e.m (*n* = 3); *p* > 0.05 for n.s. (d) Human CD4^+^ T cells were incubated with **CIR38M** or PBS and co-cultured with antigen-presenting cells in the presence of 2 μg mL^–1^ soluble anti-CD3. GM-CSF concentrations in the supernatant were determined by ELISA 72 h later. Values represented as means ± s.e.m (*n* = 3); *p* > 0.05 for n.s.

With the aim of assessing the translational potential of **CIR38M** in adoptive T cell transfer clinical studies, we labelled human CD4^+^ T cells from the peripheral blood of healthy volunteers. We observed that 2 min treatments with 10 μM **CIR38M** effectively stained human CD4^+^ T cells, with the cells remaining clearly labelled after 3 days of stimulation (Fig. 8a).

We also compared the fluorescence staining of human CD4^+^ T cells with **CIR38M** to the FDA-approved **ICG**, and observed that **CIR38M**-labelled cells were over an order of magnitude brighter than **ICG**-labelled cells under the same conditions (Fig. 8b). Subsequent culture and analysis of the **CIR38M**-labelled cells showed that the probe did not induce significant cytotoxicity in resting human CD4^+^ T cells after 24 h (Fig. 8c) or affected the cytokine expression (*e.g.* GM-CSF) of activated T cells cultured for 3 days under stimulatory conditions (Fig. 8d). The high stability and neutral character of **CIR38M** asserts its utility for longitudinal imaging studies of murine and human T cell mobility in both preclinical imaging as well as clinical research.

## Conclusions

This is the first report of tricarbocyanine *N*-triazoles as a new family of NIR dyes overcoming the limitations of tricarbocyanines for long-term *in vivo* fluorescence cell tracking. We rationally designed tricarbocyanine *N*-triazoles as a NIR structure with brighter fluorescence emission and minimal photodegradation by theoretical calculations of electron density at the bridgehead nitrogen atoms of the tricarbocyanine core. We have also developed a one-pot synthetic methodology compatible with a broad range of alkyne building blocks, making this strategy an optimal approach for the preparation of highly photostable NIR agents. We further optimised the tricarbocyanine *N*-triazole **CIR38M** as a non-transferable marker of therapeutic immune cells, both from mouse and human origin. We demonstrated that **CIR38M** can track post-transferred T cells *in vivo* at multiple time points as well as detect antigen-driven accumulation of T cells at specific sites. **CIR38M** can label small populations of T cells with high sensitivity (*i.e.* around 4000 cells) and with cells being detectable *in vivo* over 7 days post-transfer. Tricarbocyanine *N*-triazoles open multiple opportunities for *in situ* NIR imaging of therapy efficacy and disease progression in preclinical and clinical research.

## Experimental section

### General materials

Commercially available reagents were used without further purification. Thin-layer chromatography was conducted on Merck silica gel 60 F254 sheets and visualised by UV (254 and 365 nm). Silica gel (particle size 35–70 μm) was used for column chromatography. ^1^H and ^13^C spectra were recorded in a Bruker Avance 500 spectrometer (at 500 and 125 MHz, respectively). Data for ^1^H NMR spectra reported as chemical shift *δ* (ppm), multiplicity, coupling constant (Hz) and integration. Data for ^13^C NMR spectra reported as chemical shifts relative to the solvent peak. HPLC-MS analysis was performed on a Waters Alliance 2695 separation module connected to a Waters PDA2996 photodiode array detector and a ZQ Micromass mass spectrometer (ESI-MS) with a Phenomenex® column (C_18_, 5 μm, 4.6 × 150 mm). Compounds were purified using a Waters semi-preparative HPLC system using a Phenomenex® column (C_18_ Axial, 10 μm, 21.2 × 150 mm) and UV detection. HRMS (ESI positive) were obtained in a LTQ-FT Ultra (Thermo Scientific) mass spectrometer.

### Chemical synthesis

Compound **1** was synthesized following previously reported procedures.^44^^1^H NMR (500 MHz, MeOD) *δ* 7.42–7.21 (m, 12H), 7.05–6.94 (m, 4H), 5.61 (d, *J* = 12.7 Hz, 2H), 4.64–4.57 (m, 1H), 4.46–4.36 (m, 1H), 4.28–4.18 (m, 1H), 4.18–4.10 (m, 1H), 3.84 (t, *J* = 7.3 Hz, 4H), 3.64–3.56 (m, 1H), 2.68 (s, 4H), 2.39–2.31 (m, 1H), 1.91–1.75 (m, 6H), 1.60 (d, *J* = 7.1 Hz, 12H), 1.03 (t, *J* = 7.4 Hz, 6H). ^13^C NMR (126 MHz, MeOD) *δ* 128.5, 127.8, 126.9, 126.6, 121.5, 121.4, 121.1, 107.9, 91.9, 73.9, 62.5, 55.3, 46.6, 42.4, 31.4, 28.2, 28.1, 28.0, 19.4, 10.4.

### General synthesis of CIR fluorophores

To a solution of **IR780** (300 mg, 0.44 mmol, 1 eq.) in DMF (2 mL), was added sodium azide (145 mg, 2.2 mmol, 5 eq.) in H_2_O (2 mL). The resulting mixture was stirred at 70 °C for 20 min. The reaction was then cooled down, diluted with CH_2_Cl_2_ (20 mL) and washed with H_2_O (×1). Combined organic layers containing the IR780-azide intermediate (confirmed by HPLC-MS; *m*/*z*: 546) were evaporated and used without further purification. IR780-azide was dissolved in 15 mL CH_2_Cl_2_ and aliquoted to react with different alkynes (typically, in batches of 10 different alkynes). To each aliquot, we added CuSO_4_ (14 mg, 0.08 mmol, 2 eq.), tris[(1-benzyl-1*H*-1,2,3-triazol-4-yl)methyl]amine (TBTA) (42 mg, 0.08 mmol, 2 eq.) and sodium ascorbate (16 mg, 0.08 mmol, 2 eq.) pre-dissolved in DMF : H_2_O (1 : 1, 0.2 mL), and the alkynes (0.44 mmol, 10 eq.). The resulting mixtures were stirred at r.t. typically for 2 h (longer reaction times depending on the alkynes). The crude reaction mixtures were diluted in CH_2_Cl_2_ (10–20 mL), and the organic phases were washed with H_2_O (3 × 10 mL). The organic extracts were dried over MgSO_4_, filtered and evaporated under reduced pressure. The resulting crudes were then purified by semi-preparative HPLC to yield CIR fluorophores (full characterisation data in Tables S2 and S3†).

#### Compound **2**

^1^H NMR (500 MHz, MeOD) *δ* 8.89 (s, 1H), 8.03 (d, *J* = 7.0 Hz, 2H), 7.57 (dd, *J* = 8.3, 6.9 Hz, 2H), 7.49 (t, *J* = 7.5 Hz, 1H), 7.44–7.38 (m, 4H), 7.34 (d, *J* = 7.8 Hz, 2H), 7.25 (s, 2H), 6.95 (d, *J* = 14.1 Hz, 2H), 6.38 (d, *J* = 14.1 Hz, 2H), 4.17 (t, *J* = 7.4 Hz, 4H), 2.96–2.76 (m, 4H), 2.26–2.08 (m, 2H), 1.89 (q, *J* = 7.4 Hz, 4H), 1.36 (s, 6H), 1.31 (s, 6H), 1.06 (t, *J* = 7.4 Hz, 6H). ^13^C NMR (126 MHz, MeOD): *δ* 173.1, 148.1, 147.6, 142.1, 141.6, 141.1, 129.5, 128.9, 128.7, 128.5, 126.4, 125.5, 125.4, 124.8, 122.1, 111.1, 101.4, 49.1, 45.4, 26.6, 26.5, 24.1, 20.6, 20.5, 10.2.

#### Compound **CIR38M**

Chemical synthesis detailed in Scheme S1.† To a solution of CIR38-COOH (10 mg, 0.01 mmol, 1 eq.) and (1-cyano-2-ethoxy-2-oxoethylidenaminooxy) dimethylaminomorpholino-carbenium hexafluorophosphate (COMU) (10 mg, 0.02 mmol, 2 eq.) dissolved in DMF (0.4 mL) was added DIPEA (4.1 μL, 0.02 mmol, 2 eq.). The mixture was stirred at r.t. for 15 min. Next, 2-maleimidoethylamine (4.5 mg, 0.02 mmol, 2 eq.) and DIPEA (4.1 μL, 0.02 mmol, 2 eq.) were added in DMF (0.2 mL). The mixture was stirred at r.t. for 2 h. Then, H_2_O (20 mL) was added to the reaction mixture and the organic layer was extracted with CH_2_Cl_2_ (3 × 20 mL). The organic extracts were dried over MgSO_4_, filtered and evaporated under reduced pressure. The resulting crude was then purified by semi-preparative HPLC to render **CIR38M** as a green solid (4 mg, 37% yield).

^1^H NMR (500 MHz, MeOD) *δ* 8.85 (s, 1H), 8.67 (d, *J* = 4.9 Hz, 1H), 8.30 (d, *J* = 7.9 Hz, 1H), 8.06 (t, *J* = 7.8 Hz, 1H), 7.50 (d, *J* = 4.9 Hz, 1H), 7.42–7.36 (m, 3H), 7.35–7.31 (m, 2H), 7.27–7.21 (m, 2H), 6.92 (dd, *J* = 14.1, 4.8 Hz, 2H), 6.82 (s, 1H), 6.37 (d, *J* = 14.1 Hz, 2H), 4.22–4.09 (m, 4H), 3.62 (dt, *J* = 750.8, 5.8, 4.1 Hz, 3H), 3.39–3.34 (m, 3H), 2.88 (s, 4H), 2.66 (d, *J* = 9.4 Hz, 1H), 2.14 (t, *J* = 7.3 Hz, 3H), 1.92–1.78 (m, 4H), 1.69–1.60 (m, 4H), 1.35 (s, 6H), 1.27 (s, 6H), 1.04 (t, *J* = 7.4 Hz, 3H). ^13^C NMR (126 MHz, MeOD) *δ* 174.7, 173.1, 172.9, 171.1, 149.6, 148.9, 148.0, 147.3, 142.1, 142.0, 141.5, 141.4, 141.2, 141.1, 137.7, 134.1, 128.5, 126.5, 125.4, 125.4, 123.7, 122.1, 120.4, 111.1, 111.1, 101.6, 101.5, 49.1, 49.1, 48.2, 45.4, 43.8, 37.5, 37.1, 35.1, 26.8, 26.6, 26.5, 25.9, 24.8, 24.1, 24.1, 20.6, 20.4, 10.2. HRMS: *m*/*z* [M^+^] calcd for C_52_H_59_N_8_O_3_: 843.4705; found: 843.4660.

### *In vitro* spectral measurements

Spectroscopic and quantum yield data were recorded on a Synergy HT spectrophotometer (Biotek). Compounds were dissolved at the indicated concentrations and spectra were recorded at r.t. Spectra are represented as means from at least two independent experiments with *n* = 3. Relative quantum yields were calculated by measuring the integrated emission area of the fluorescence spectra and comparing it to the area measured for the standard^56^ (*e.g.* for CIR fluorophores, **ICG** was used as the reference).

### Calculations

All quantum chemical calculations were performed with Gaussian 09 (Revision D.01).^27^ Ground state geometries of compounds **1** and **2** were optimised using the density function theory (DFT) with B3LYP^57^,^58^ M06-2X,^59^ PBE0 (ref. 60) and wB97xd^61^ functionals together with the Pople's basis set 6-31G*. Geometry optimisations were performed both in the gas phase and in EtOH, where the solvent was described by the Polarizable Continuum Model (PCM).^62^ The nature of the excitation energies, oscillator strengths and contributions of the different orbitals involved in the electronic transitions were calculated using time-dependent density functional theory (TD-DFT) at the selected levels of theory, both in gas phase and in EtOH.

### Particle size analysis

The mean size of aggregates in aqueous media were determined by dynamic light scattering using a PS90 Particle Size Analyser (Brookhaven Instrument Corporation). **IR780** and **CIR38** were dissolved in PBS (100 μM) and kept at r.t. to measure the size of aggregates every 2 h for a total of 8 h. Data is represented as means ± s.e.m from two independent experiments with *n* = 3.

### *In vitro* labelling and characterisation of CD4^+^ T cells

Single cell suspensions were made from the spleen after which red blood cells were lysed using NH_4_Cl buffer. CD4^+^ T cells were purified by magnetic cell sorting as per manufacturer's instructions (Miltenyi Biotech). CD4^+^ T cells were re-suspended in PBS and then labelled with the probes for 2 min at the stated concentrations at 37 °C. Cells were washed twice with cell culture medium, after which they were re-suspended in FACS buffer (PBS, 2% FCS, 0.01% sodium azide) or appropriate cell culture medium. For flow cytometric analysis, cells were then incubated with antibodies for 20 min at 4 °C. The antibodies used were anti-CD4-e450 or anti-CD4-PE, anti-CD45.1-FITC or anti-CD45.1-PE, and anti-CD11b-APC (all from eBioscience). Samples were also stained with a fixable viability dye (conjugated with eFluor455, eBioscience) prior to surface staining. Flow cytometry data were collected using an LSR Fortessa (BD Biosciences) and analysed using FlowJo software.

### *In vitro* cell viability and proliferation assays

To study the primary activation of **CIR38M**-labelled CD4^+^ T cells, 2 × 10^5^ CD4^+^ Tg4. CD45.1^+^ T cells were added per well to 2 μg mL^–1^ anti-CD3 and 2 μg mL^–1^ anti-CD28 coated, flat-bottomed 96-well plates. After 48 h, cell proliferation was assessed by the addition of [^3^H]-thymidine at 0.5 μCi per well for the last 18 h of culture. [^3^H]-thymidine incorporation was measured using a scintillation β-counter (Wallac) as mean counts per minute (c.p.m). The production of cytokines was assessed in culture supernatants by ELISA using Ready-SET-Go ELISA kits according to manufacturer's instructions after 72 h of culture. In some experiments, CD45.1^+^ and CD45.2^+^ CD4^+^ T cells were co-cultured together to assess the extent of probe transfer between cells. In addition, in some instances, cells were double-labelled with 5 μM CFSE and 10 μM **CIR38M** for 2 min at 37 °C.

### Fluorescence microscopy of CD4^+^ T cells

Single cell suspensions were made from spleens of C57BL/6 mice and red blood cells lysed using NH_4_Cl buffer. CD4^+^ T cells were purified by magnetic cell sorting as per manufacturer's instructions, re-suspended in PBS and then labelled with CellTracker Green (10 μM), MitoTracker Red CMXRos (500 nM) or LysoTracker Red DND-99 (100 nM) at 37 °C, prior to addition of **CIR38M** or **IR800CW-SE** (10 μM) and incubation at 37 °C. Cells were washed twice with cell culture medium, twice in PBS, before re-suspension of the cell pellet in 1% PFA. T cells were mounted and imaged under a fluorescence microscope (Nikon Ti Eclipse) using a 60× oil immersion objective (NA 1.4) and a Hamamatsu Orca Flash 4.0 V2 camera for NIR detection. Excitation was achieved either by a Ti-sapphire laser (Coherent Mira 900, 740 nm CW) or a 100 W mercury lamp and a NIR epifluorescence filter cube (EX 747/33, DM 776LP, EM 776LP).

### Whole-body fluorescence *in vivo* imaging and *ex vivo* analysis

C57BL/6 (CD45.1^–^ CD45.2^+^) and OT-II (CD45.1^+^ CD45.2^–^) mice were bred under specific pathogen-free conditions at the University of Edinburgh. Hair from C57BL/6 mice was removed using hair clippers followed by application of a coat of Nair and subsequent wiping with gauze sponges and water. Hairless albino C57BL/6 (B6.Cg-Tyrc-2J Hrhr/J) mice were purchased from The Jackson Laboratory. The housing facility was compliant with Federation of European Laboratory Animal Science Associations guidelines on screening mice for infectious diseases. All experiments had local ethical approval from the University of Edinburgh's Animal Welfare and Ethical Review Body and were performed in accordance with UK legislation. OT-II transgenic mice express an I-A^b^-restricted T cell receptor, which is reactive toward ovalbumin peptide 323–339.^63^ The ovalbumin peptide (pOVA) was obtained from Cambridge Research Biochemicals. Tissue culture medium (RPMI 1640 medium) was supplemented with 2 mM l-glutamine, 100 U mL^–1^ penicillin, 100 μg mL^–1^ streptomycin, 5 × 10^–5^ M 2-ME (all from Invitrogen Life Technologies) and 10% FCS (Labtech).

Albino hairless female C57BL/6 (CD45.2) mice were intravenously injected with 20 × 10^6^ CD45.1^+^ CD4^+^ OT-II T cells which had been labelled or not with 10 μM **CIR38M** for 2 min. On the same day, mice received a 50 μL subcutaneous injection of 10 μg of pOVA peptide emulsified in 2 mg mL^–1^ CFA, or PBS and CFA into each hind leg flank. Whole-body *in vivo* fluorescence images (*λ*_exc._ ∼ 760 nm; *λ*_em._ ∼ 800–900 nm) of representative mice from both groups (*i.e.* injected with **CIR38M**-labelled cells or injected with unlabelled cells) were acquired on a PhotonImager™ (Biospace Lab) on days 2, 4 and 7. Mice were then culled to harvest the spleens and draining inguinal lymph nodes for *ex vivo* tissue imaging and analysis by flow cytometry as described above.

For **CIR38M***vs.***DiR** comparative analysis, 10^6^ CD4^+^ Tg4 T cells were transferred into B10.PLxC57BL/6 mice followed by subcutaneous immunisation with 10 μg of MBP Ac1-9(4Tyr) peptide emulsified in CFA containing 50 μg of heat-killed *Mycobacterium tuberculosis* H37Ra at a total final volume of 100 μL injected subcutaneously into the hind legs. Whole-body fluorescence images of representative mice from both groups (*i.e.* injected with **CIR38M**-labelled cells or **DiR**-labelled cells) were acquired on a PhotonImager™ (Biospace Lab) 48 h post-injection.

### *Ex vivo* labelling of human CD4^+^ T cells

*Ex vivo* experiments with fresh human peripheral blood from healthy donors were approved by the Accredited Medical Regional Ethics Committee (AMREC, reference number 15-HV-013), as previously reported.^64^ Human CD4^+^ T cells were purified by magnetic cell sorting as per manufacturer's instructions (Miltenyi Biotech). CD4^+^ T cells were re-suspended in PBS and then labelled with **CIR38M** or **ICG** (10 μM) at 37 °C. Cells were washed twice with cell culture medium, after which they were re-suspended in FACS buffer or appropriate culture medium. Proliferation studies were undertaken by adding 2 × 10^5^ CD4^+^ T cells per well to equal amounts of antigen-presenting cells and 2 μg mL^–1^ soluble anti-CD3 in flat-bottomed 96-well plates. The production of cytokines was assessed in culture supernatants by ELISA using Ready-SET-Go ELISA kits according to manufacturer's instructions (eBioscience) after 72 h of culture.

## Conflicts of interest

The University of Edinburgh has submitted a priority file to protect the technology described in the study.

## Supplementary Material

Supplementary information
