# Supplementary material for: Tricarbocyanine N-triazoles: the scaffold-of-choice for long-term near-infrared imaging of immune cells in vivo
Source: Chem Sci. 2018 Aug 8;9(36):7261–70. doi: 10.1039/c8sc00900g (PMC6148684; doi:10.1039/c8sc00900g)
Supplement: Supplementary file 1 [file SC-009-C8SC00900G-s001.pdf]

Electronic Supplementary Material (ESI) for Chemical Science.
This journal is © The Royal Society of Chemistry 2018

Electronic Supplementary Information

Tricarbocyanine-*N*-triazoles: The Scaffold-of-Choice for Long-Term Near-Infrared Imaging of Immune Cells *in vivo*

Richard J. Mellanby, Jamie I. Scott, Iris Mair, Antonio Fernandez, Louise Saul, Jochen Arlt, Monica Moral, Marc Vendrell

Table of Contents:

Supplementary Methods and NMR spectra

Supplementary Schemes

Supplementary Figures

Supplementary Tables

Supplementary Methods

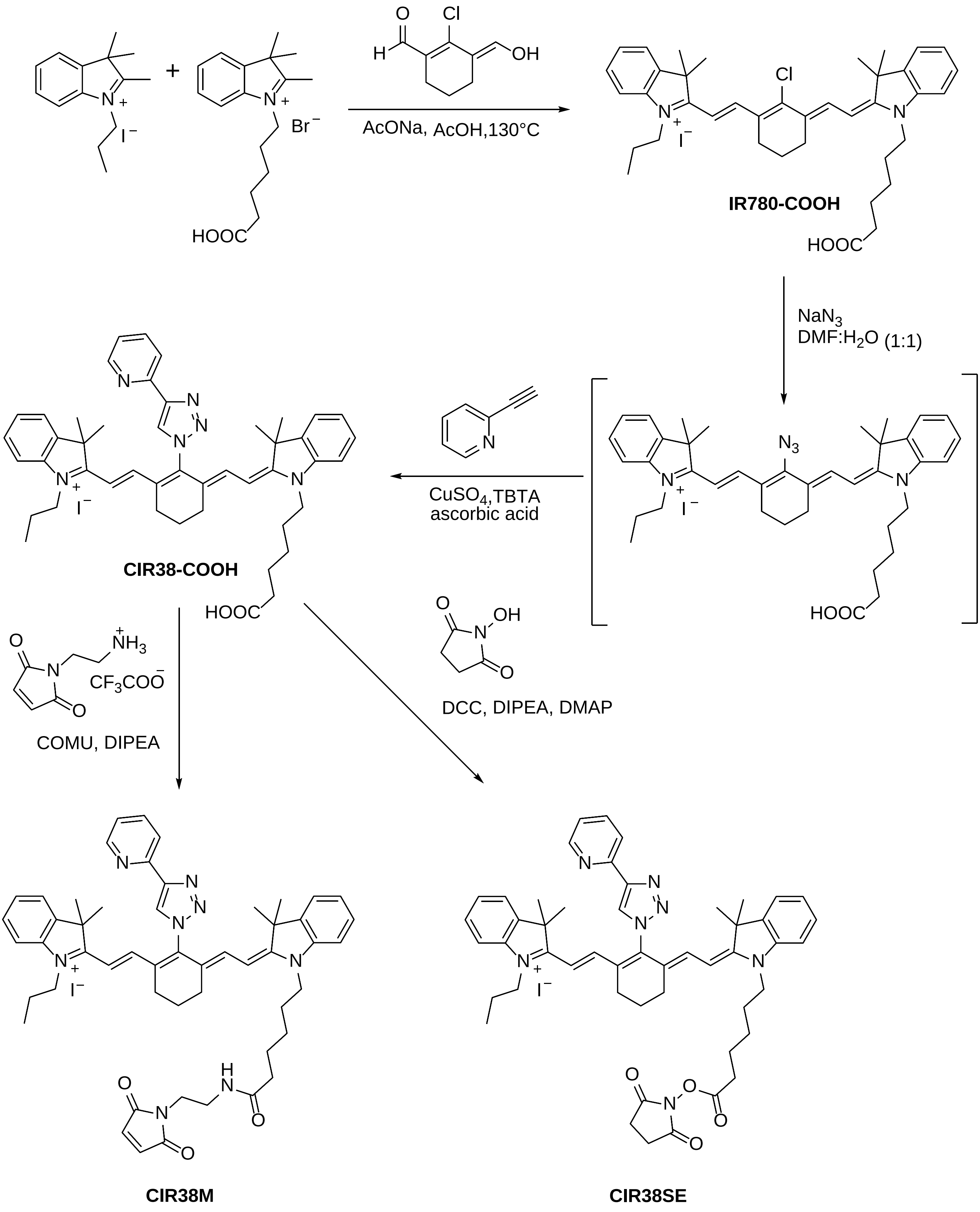

Scheme S1. Synthetic scheme for the preparation of **CIR38** derivatives.

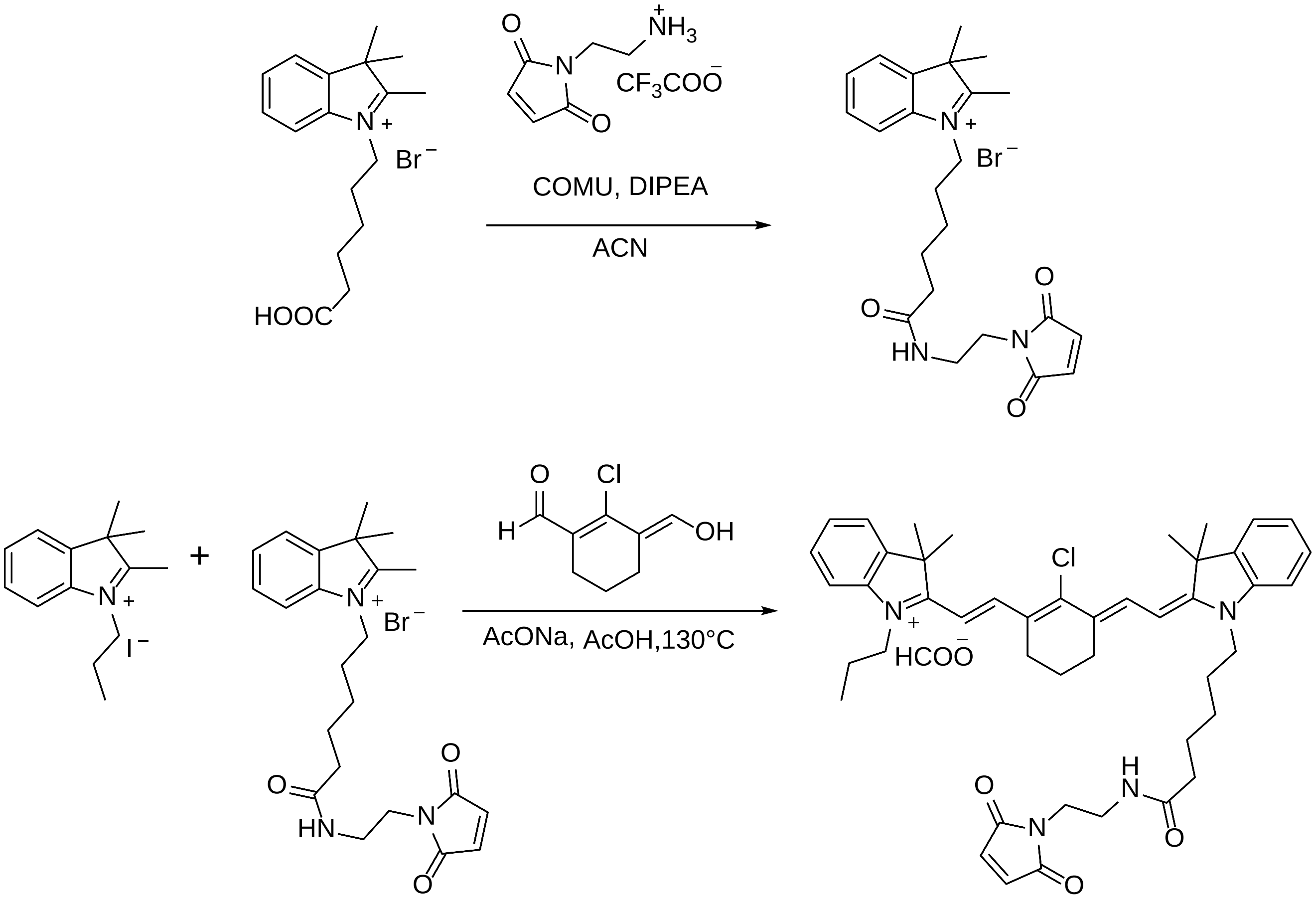

Scheme S2. Synthetic scheme for the preparation of IR780-maleimide **(IR780M)**.

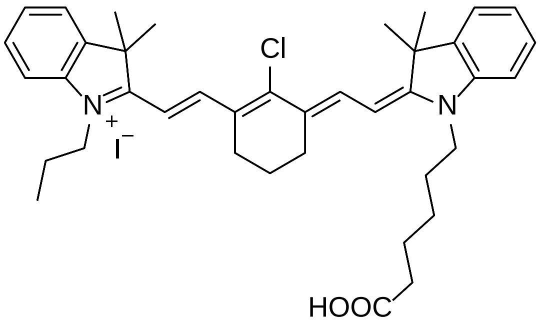

IR780-COOH

IR780-COOH. To a solution of sodium acetate (258 mg, 3.15 mmol) in AcOH (20 mL) was added 2-chloro-3-(hydroxymethylene)-1-cyclohexene-1-carboxaldehyde (86 mg, 0.5 mmol) and 3*H*-indolium,1-(5-carboxypentyl)-2,3,3-trimethylbromide (214 mg, 0.6 mmol). The resulting solution was stirred at 130 ^oC under reflux for 2 h. To the reaction was then added 3*H*-indolium,2,3,3-trimethyl-1-propyl iodide (148 mg, 0.5 mmol) and the mixture was stirred overnight at 130 ^oC. The reaction was then cooled to r.t. and solvents were evaporated under reduced pressure. The resulting crude was then purified by column chromatography (CH_2Cl_2: MeOH, 98:2) to isolate **IR780-COOH** as a green solid (120 mg, 32% yield).

^{1}H NMR (500 MHz, MeOD) δ 8.45 (dd, *J* = 14.1, 5.0 Hz, 2H), 7.57 – 7.51 (m, 2H), 7.44 (ddt, *J* = 8.4, 7.5, 1.1 Hz, 2H), 7.38 – 7.33 (m, 2H), 7.30 (tdd, *J* = 7.4, 3.0, 0.9 Hz, 2H), 6.31 (dd, *J* = 14.1, 9.5 Hz, 2H), 4.19 (dt, *J* = 9.9, 7.5 Hz, 4H), 2.75 (t, *J* = 6.3 Hz, 3H), 2.32 (t, *J* = 7.3 Hz, 2H), 1.99 (s, 3H), 1.94 – 1.86 (m, 4H), 1.80 (d, *J* = 2.8 Hz, 1H), 1.74 (s, 10H), 1.73 – 1.67 (m, 2H), 1.63 (s, 1H), 1.56 – 1.48 (m, 2H), 1.07 (t, *J* = 7.4 Hz, 3H). **^{13}C NMR** (126 MHz, MeOD) δ 176.3, 174.2, 173.1, 172.8, 149.7, 144.2, 144.0, 142.3, 142.2, 141.2, 141.2, 132.8, 132.8, 129.8, 129.1, 128.5, 128.5, 126.6, 125.2, 125.1, 123.3, 122.1, 115.2, 111.0, 110.9, 101.1, 100.9, 53.4, 45.4, 43.7, 33.6, 27.0, 26.0, 24.4, 21.5, 20.7, 20.5, 19.6, 10.3.

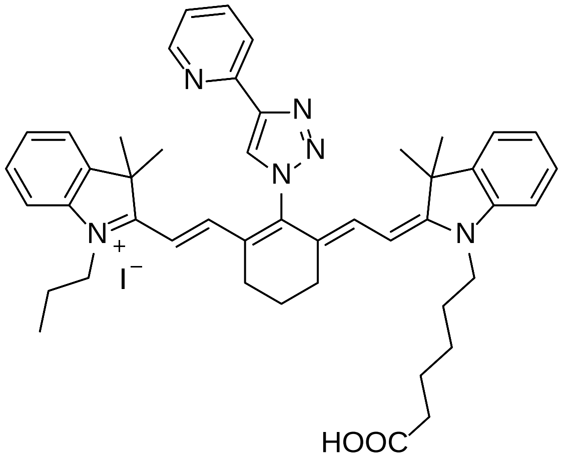

CIR38-COOH

CIR38-COOH. To a solution of **IR780-COOH** (57 mg, 0.08 mmol) in DMF (1 mL) was added sodium azide (25 mg, 0.4 mmol) in H_2O (1 mL). The resulting mixture was stirred at r. t. for 20 min. To the reaction was then added 2-ethynylpyridine (79 mg, 0.8 mmol), $CuSO_4$ (25 mg, 0.16 mmol), TBTA (85 mg, 0.16 mmol) and sodium ascorbate (32 mg, 0.16 mmol) and the mixture was stirred at r. t. for 2 h. The reaction crude was diluted in CH_2Cl_2 (20 mL), and the organic phase was washed with H_2O (3 × 10 mL). The organic extracts were dried over $MgSO_4$, filtered and evaporated under reduced pressure. The crude was then purified by normal-phase chromatography (CH_2Cl_2: MeOH, 95:5) to yield **CIR38-COOH** as a green solid (14 mg, 20% yield).

^{1}H NMR (500 MHz, MeOD) δ 8.87 (s, 1H), 8.70 (s, 1H), 8.32 (d, *J* = 7.9 Hz, 1H), 8.06 (t, *J* = 7.8 Hz, 1H), 7.53 – 7.48 (m, 1H), 7.45 – 7.36 (m, 4H), 7.34 (d, *J* = 2.5 Hz, 2H), 7.27 – 7.21 (m, 2H), 6.92 (dd, *J* = 14.1, 3.0 Hz, 2H), 6.37 (dd, *J* = 14.1, 6.2 Hz, 2H), 4.22 – 4.13 (m, 4H), 2.89 (s, 4H), 2.31 (t, *J* = 7.3 Hz, 3H), 2.22 – 2.11 (m, 2H), 1.91 – 1.82 (m, 4H), 1.75 – 1.67 (m, 3H), 1.35 (s, 6H), 1.28 (s, 6H), 1.05 (t, *J* = 7.4 Hz, 3H).

^{13}C NMR (126 MHz, MeOD) δ 173.1, 172.9, 149.5, 148.9, 147.3, 142.1, 142.0, 141.4, 141.4, 141.1, 137.7, 128.6, 128.5, 128.5, 126.5, 126.5, 125.4, 125.4, 122.1, 111.2, 111.1, 101.6, 101.5, 49.1, 49.1, 45.4, 43.8, 26.8, 26.6, 26.0, 24.6, 24.1, 24.1, 20.6, 20.4, 10.2.

HRMS: m/z [M^+] calcd. for $C_{46}H_{53}O_2N_6$: 721.4225; found: 721.4206.

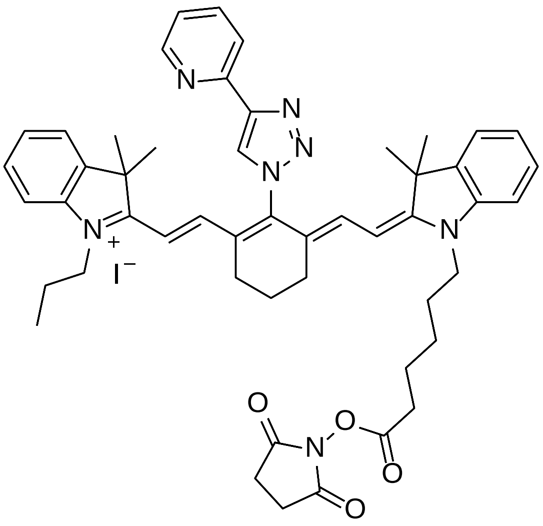

CIR38SE

CIR38SE. CIR38-COOH (9 mg, 0.01 mmol), *N*-hydroxysuccinimide (4 mg, 0.03 mmol), *N,N'*-dicyclohexylcarbodiimide (10 mg, 0.04 mmol) and DIPEA (4 μL, 0.04 mmol) were dissolved in $CHCl_3$ (1.5 mL) with a catalytic amount of DMAP. The reaction was stirred at r.t. for 2 h. The reaction crude was purified by preparative TLC (CH_2Cl_2: MeOH, 95:5) to yield **CIR38SE** as a green solid (7 mg, 81% yield).

^{1}H NMR (500 MHz, MeOD) δ 8.86 (s, 1H), 8.67 (d, *J* = 4.6 Hz, 1H), 8.31 (d, *J* = 8.0 Hz, 1H), 8.06 (td, *J* = 7.8, 1.8 Hz, 1H), 7.52 – 7.48 (m, 1H), 7.43 – 7.37 (m, 4H), 7.33 (d, *J* = 10.6 Hz, 2H), 7.24 (t, *J* = 7.9 Hz, 2H), 6.92 (d, *J* = 14.2 Hz, 2H), 6.37 (dd, *J* = 14.1, 4.1 Hz, 2H), 4.23 – 4.11 (m, 4H), 2.90 – 2.85 (m, 4H) 2.86 (s, 4H), 1.92 – 1.81 (m, 5H), 1.64 – 1.53 (m, 2H), 1.35 (s, 6H), 1.27 (s, 6H), 1.05 (t, *J* = 7.4 Hz, 3H), 0.95 – 0.84 (m, 5H). **^{13}C NMR** (126 MHz, MeOD) δ 173.6, 173.0, 171.3, 170.4, 149.6, 148.9, 148.0, 147.3, 145.5, 141.9, 141.1, 137.8, 129.4, 128.5, 126.5, 125.4, 123.7, 122.1, 120.4, 114.5, 111.1, 111.1, 107.4, 104.7, 101.6, 100.6, 96.1, 53.6, 45.4, 45.3, 43.9, 43.8, 31.7, 30.5, 30.0, 29.3, 26.6, 25.5, 25.1, 24.9, 24.1, 23.9, 22.3, 20.6, 20.4, 13.0, 10.2.

HRMS: m/z [M^+] calcd. for $C_{50}H_{56}N_7O_4$: 818.4388; found: 818.4453.

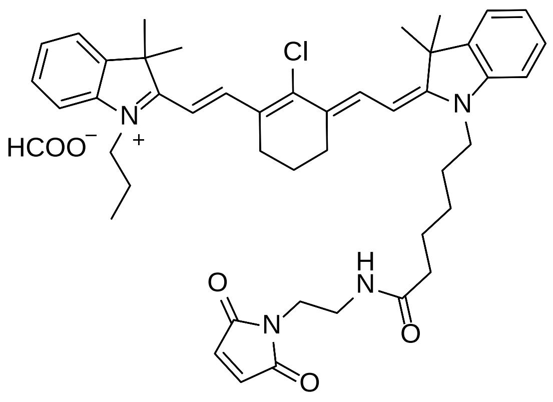

IR780M

IR780M. To a solution of 3*H*-indolium,1-(5-carboxypentyl)-2,3,3-trimethylbromide (50 mg, 0.14 mmol) and 1-cyano-2-ethoxy-2-oxoethylidenaminooxy) dimethylaminomorpholino-carbeniumhexafluorophosphate (COMU) (90 mg, 0.21 mmol) dissolved in ACN (0.4 mL), DIPEA (37μL, 0.21 mmol) was added. The mixture was stirred at r.t. for 10 min. Next, 2-maleimidoethylamine (36 mg, 0.21 mmol) and DIPEA (3 7μL, 0.21mmol) were added in ACN (0.2 mL) and the mixture was stirred at r.t. for 2 h. Then, H_2O (20 mL) was added to the reaction mixture which was extracted with CH_2Cl_2 (3 × 20 mL). The organic extracts were dried over $MgSO_4$, filtered and evaporated under reduced pressure. The resulting crude was used in the next step without any purification. To a solution of AcONa (65 mg, 0.80 mmol) in AcOH (5 mL), 2-chloro-3-(hydroxymethylene)-1-cyclohexene-1-carboxaldehyde (21 mg, 0.14mmol) and 3*H*-indolium,2,3,3-trimethyl-1-propyl iodide (37 mg, 0.14 mmol) were added. The resulting solution was stirred at 130ºC under reflux for 2 h. Next, 1-(6-((2-(2,5-dioxo-2,5-dihydro-1H-pyrrol-1-yl)ethyl)amino)-6-oxohexyl)-2,3,3-trimethyl-3H-indol-1-ium bromide (56 mg, 0.14 mmol) was added to the reaction mixture, which was stirred for 2 h at 130 ºC. The reaction was then cooled to r.t. and solvents were evaporated under reduced pressure. The resulting crude was then purified by semi-preparative HPLC to isolate **IR780-M** as a green solid (2 mg).

^{1}H NMR (500 MHz, MeOD) δ 8.50 – 8.43 (m, 1H), 7.55 (ddd, *J* = 7.6, 3.7, 1.1 Hz, 2H), 7.46 (td, *J* = 7.7, 1.2 Hz, 2H), 7.41 – 7.34 (m, 2H), 7.32 (tdd, *J* = 7.5, 3.0, 0.9 Hz, 2H), 6.81 (s, 2H), 6.32 (d, *J* = 14.1 Hz, 1H), 5.51 (s, 2H), 4.25 – 4.13 (m, 2H), 4.12 – 4.03 (m, 2H), 3.89 – 3.79 (m, 4H), 3.60 –

3.54 (m, 4H), 2.80 – 2.69 (m, 2H), 2.37 (t, J = 7.4 Hz, 2H), 1.76 (s, 6H), 1.76 (s, 6H), 1.69 – 1.59 (m, 2H), 1.38 – 1.31 (m, 5H), 1.08 (t, J = 7.4 Hz, 3H), 0.98 – 0.88 (m, 2H).

HRMS: m/z [M^+] calcd. for $C_{45}H_{54}ClN_4O_3$: 733.3879; found: 733.3849.

CIR library (representative subset)

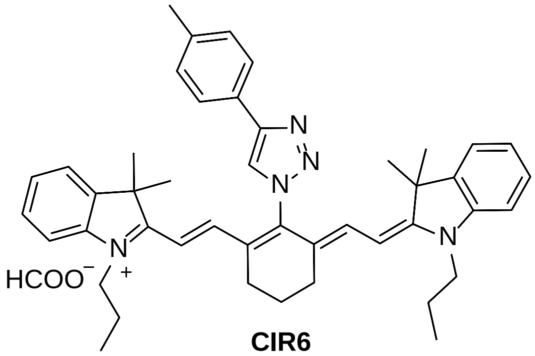

CIR6

^{1}H NMR (500 MHz, MeOD) δ 8.81 (s, 1H), 7.89 (d, *J* = 8.1 Hz, 2H), 7.45 – 7.34 (m, 5H), 7.36 – 7.29 (m, 2H), 7.28 – 7.20 (m, 2H), 6.94 (d, *J* = 14.2 Hz, 3H), 6.36 (d, *J* = 14.2 Hz, 2H), 4.16 (t, *J* = 7.3 Hz, 2H), 2.91 – 2.83 (m, 2H), 2.45 (s, 3H), 1.87 (q, *J* = 7.4 Hz, 4H), 1.35 (s, 6H), 1.30 (s, 6H), 1.25 – 1.12 (m, 4H), 1.04 (t, *J* = 7.4 Hz, 6H), 1.01 – 0.93 (m, 2H). **^{13}C NMR** (126 MHz, MeOD) δ 173.1, 148.2, 147.7, 142.1, 141.6, 141.1, 138.9, 129.5, 128.5, 126.6, 126.4, 125.5, 125.4, 124.5, 122.1, 111.1, 101.4, 49.1, 45.4, 26.6, 24.1, 20.6, 20.0, 10.2.

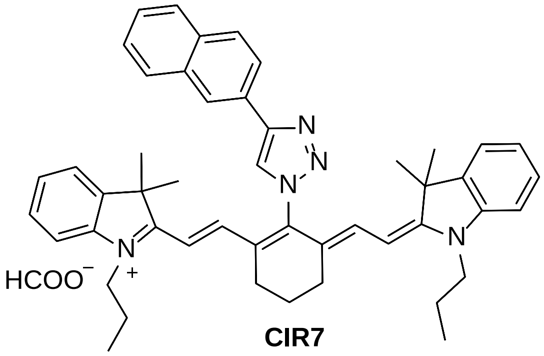

CIR7

^{1}H NMR (500 MHz, MeOD) δ 9.01 (s, 1H), 8.55 (s, 1H), 8.12 (dd, *J* = 8.5, 1.7 Hz, 1H), 8.08 – 8.03 (m, 1H), 8.04 – 7.99 (m, 1H), 7.96 (dd, *J* = 7.8, 1.9 Hz, 1H), 7.63 – 7.55 (m, 2H), 7.44 – 7.35 (m, 4H), 7.32 (dt, *J* = 8.0, 0.8 Hz, 2H), 7.21 (td, *J* = 7.5, 1.0 Hz, 2H), 6.99 (d, *J* = 14.1 Hz, 2H), 6.38 (d, *J* = 14.1 Hz, 2H), 4.16 (t, *J* = 7.4 Hz, 2H), 2.89 (q, *J* = 6.7 Hz, 2H), 1.88 (q, *J* = 7.4 Hz, 4H), 1.37 (s, 6H), 1.30 (s, 6H), 1.24 – 1.15 (m, 4H), 1.05 (t, *J* = 7.4 Hz, 6H), 1.03 – 0.94 (m, 2H). **^{13}C NMR** (126 MHz, MeOD) δ 173.1, 148.1, 147.7, 142.1, 141.6, 141.1, 133.7, 133.6, 128.8, 128.4, 127.9, 127.5, 126.8, 126.5, 126.4, 125.4, 125.1, 124.3, 123.2, 122.2, 111.1, 101.4, 49.1, 45.4, 26.6, 24.1, 20.6, 10.2.

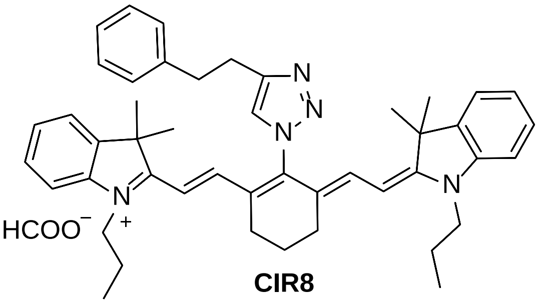

CIR8

¹H NMR (500 MHz, MeOD) δ 8.19 (s, 1H), 7.50 – 7.42 (m, 3H), 7.44 – 7.38 (m, 4H), 7.40 – 7.31 (m, 4H), 7.32 – 7.25 (m, 2H), 6.80 (d, *J* = 14.1 Hz, 2H), 6.33 (d, *J* = 14.2 Hz, 2H), 4.15 (t, *J* = 7.4 Hz, 2H), 3.32 – 3.25 (m, 2H), 3.22 – 3.16 (m, 2H), 2.89 – 2.78 (m, 2H), 1.87 (q, *J* = 7.4 Hz, 4H), 1.34 (s, 6H), 1.22 (s, 6H), 1.05 (t, *J* = 7.4 Hz, 6H), 1.02 – 0.85 (m, 6H). **¹³C NMR** (126 MHz, MeOD) δ 173.0, 148.0, 142.1, 141.6, 141.1, 140.8, 128.5, 128.3, 128.3, 128.0, 128.0, 126.5, 126.1, 125.4, 123.9, 122.1, 111.1, 101.3, 49.1, 45.3, 35.3, 26.6, 24.0, 20.5, 10.2.

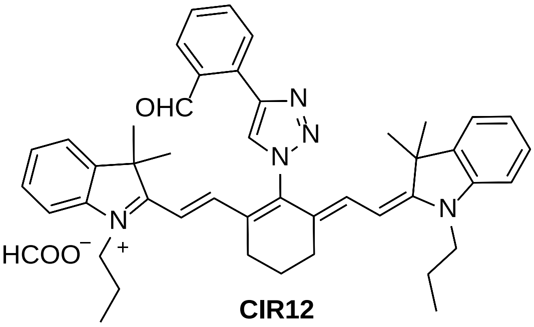

CIR12

¹H NMR (500 MHz, MeOD) δ 10.57 (s, 1H), 8.90 (s, 1H), 8.66 (s, 1H), 8.21 – 8.10 (m, 2H), 7.96 – 7.83 (m, 2H), 7.73 (ddd, *J* = 7.8, 1.4, 0.7 Hz, 1H), 7.47 – 7.38 (m, 2H), 7.34 (dt, *J* = 7.9, 0.8 Hz, 2H), 7.27 (td, *J* = 7.5, 0.9 Hz, 2H), 6.94 (d, *J* = 14.2 Hz, 2H), 6.39 (d, *J* = 14.1 Hz, 2H), 4.26 – 4.09 (m, 2H), 2.98 – 2.83 (m, 2H), 1.89 (q, *J* = 7.4 Hz, 4H), 1.40 (s, 6H), 1.34 (s, 6H), 1.26 – 1.14 (m, 4H), 1.05 (t, *J* = 7.4 Hz, 6H), 1.03 – 0.88 (m, 2H). **¹³C NMR** (126 MHz, MeOD) δ 191.8, 173.0, 142.1, 141.4, 141.1, 134.0, 130.1, 129.4, 129.2, 128.5, 127.9, 126.6, 125.5, 122.1, 111.2, 101.6, 49.2, 45.4, 26.7, 24.1, 20.6, 10.2.

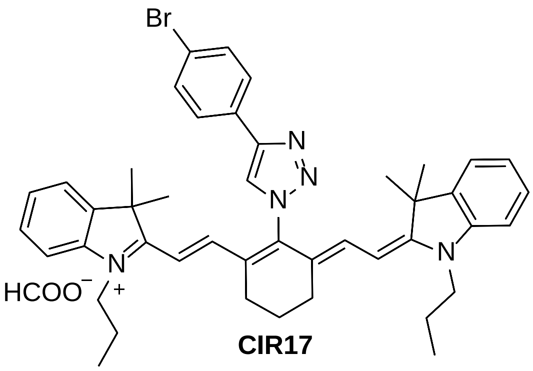

CIR17

^{1}H NMR (500 MHz, MeOD) δ 8.90 (s, 1H), 7.95 (d, *J* = 8.5 Hz, 1H), 7.72 (d, *J* = 8.6 Hz, 2H), 7.46 – 7.37 (m, 5H), 7.36 – 7.30 (m, 2H), 7.26 (dd, *J* = 7.4, 0.9 Hz, 2H), 6.92 (d, *J* = 14.2 Hz, 2H), 6.36 (d, *J* = 14.1 Hz, 2H), 4.16 (t, *J* = 7.3 Hz, 2H), 2.93 – 2.82 (m, 2H), 1.87 (q, *J* = 7.4 Hz, 4H), 1.35 (s, 6H), 1.29 (s, 6H), 1.23 – 1.12 (m, 4H), 1.04 (t, *J* = 7.4 Hz, 6H), 1.00 – 0.86 (m, 2H). **^{13}C NMR** (126 MHz, MeOD) δ 173.1, 147.5, 147.0, 142.1, 141.5, 141.1, 132.2, 128.5, 127.2, 126.4, 125.4, 122.2, 111.1, 101.4, 49.1, 45.4, 26.6, 24.1, 20.6, 10.2.

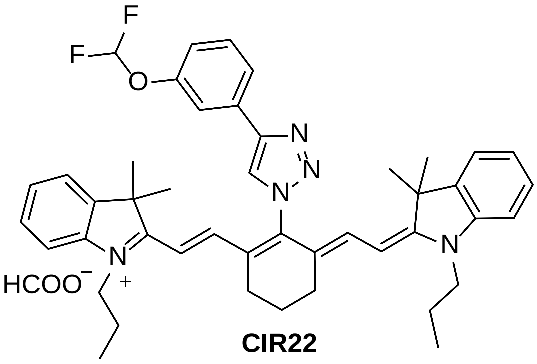

CIR22

^{1}H NMR (500 MHz, MeOD) δ 8.95 (s, 1H), 7.91 – 7.80 (m, 2H), 7.59 (t, *J* = 8.0 Hz, 1H), 7.43 – 7.38 (m, 3H), 7.38 – 7.31 (m, 2H), 7.29 – 7.22 (m, 3H), 7.13 (s, 1H), 6.98 (s, 1H), 6.92 (d, *J* = 14.1 Hz, 2H), 6.37 (d, *J* = 14.1 Hz, 2H), 4.16 (t, *J* = 7.4 Hz, 2H), 2.92 – 2.84 (m, 2H), 1.87 (q, *J* = 7.4 Hz, 4H), 1.35 (s, 6H), 1.30 (s, 6H), 1.21 – 1.10 (m, 4H), 1.05 (t, *J* = 7.4 Hz, 6H), 0.99 – 0.94 (m, 2H). **^{13}C NMR** (126 MHz, MeOD) δ 173.1, 152.2, 147.5, 147.0, 141.5, 131.5, 130.6, 128.5, 126.4, 125.4, 125.4, 122.3, 122.1, 119.1, 116.3, 116.2, 111.2, 101.5, 49.1, 45.4, 26.6, 26.6, 24.1, 20.6, 20.5, 10.2.

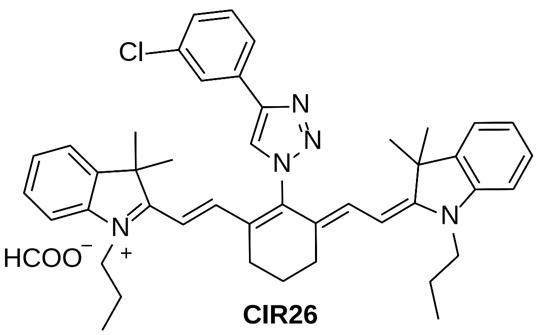

CIR26

^{1}H NMR (500 MHz, MeOD) δ 8.94 (s, 1H), 8.56 (s, 1H), 8.08 (t, *J* = 1.8 Hz, 1H), 8.01 – 7.93 (m, 2H), 7.88 (d, *J* = 8.0 Hz, 1H), 7.59 – 7.52 (m, 1H), 7.49 (ddd, *J* = 8.1, 2.1, 1.1 Hz, 2H), 7.45 – 7.36 (m, 2H), 7.36 – 7.23 (m, 2H), 6.92 (d, *J* = 14.3 Hz, 2H), 6.36 (d, *J* = 14.1 Hz, 2H), 4.16 (t, *J* = 7.3 Hz, 2H), 2.87 (q, *J* = 7.5, 7.0 Hz, 2H), 1.87 (q, *J* = 7.4 Hz, 4H), 1.36 (s, 6H), 1.30 (s, 6H), 1.26 – 1.12 (m, 4H), 1.05 (t, *J* = 7.4 Hz, 6H), 1.02 – 0.91 (m, 2H). **^{13}C NMR** (126 MHz, MeOD) δ 173.1, 142.1, 141.5, 141.1, 130.6, 128.5, 126.4, 125.5, 125.3, 123.7, 111.1, 101.4, 49.1, 45.4, 26.6, 24.1, 20.6, 10.2.

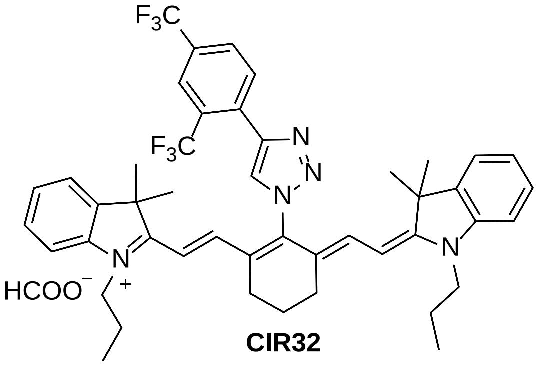

CIR32

^{1}H NMR (500 MHz, MeOD) δ 9.19 (s, 1H), 8.72 – 8.62 (m, 2H), 8.10 (dt, *J* = 1.9, 0.9 Hz, 1H), 7.40 (dd, *J* = 7.4, 1.3 Hz, 4H), 7.39 – 7.31 (m, 2H), 7.25 (td, *J* = 7.4, 0.9 Hz, 2H), 6.92 (d, *J* = 14.1 Hz, 2H), 6.38 (d, *J* = 14.1 Hz, 2H), 4.17 (t, *J* = 7.3 Hz, 2H), 2.96 – 2.82 (m, 2H), 1.88 (q, *J* = 7.4 Hz, 4H), 1.36 (s, 6H), 1.29 (s, 6H), 1.21 – 1.18 (m, 4H), 1.05 (t, *J* = 7.4 Hz, 6H), 0.99 – 0.95 (m, 2H). **^{13}C NMR** (126 MHz, MeOD) δ 173.1, 147.1, 145.1, 142.1, 141.3, 141.1, 132.4, 128.5, 126.4, 125.5, 122.2, 111.2, 101.6, 49.1, 45.4, 26.6, 26.4, 24.1, 20.6, 10.2.

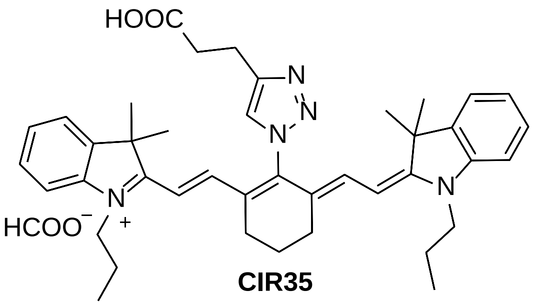

CIR35

^{1}H NMR (500 MHz, MeOD) δ 8.21 (s, 1H), 7.47 (dd, *J* = 7.6, 1.1 Hz, 2H), 7.46 – 7.39 (m, 2H), 7.34 (d, *J* = 7.9 Hz, 2H), 7.28 (td, *J* = 7.5, 0.9 Hz, 2H), 6.80 (d, *J* = 14.1 Hz, 2H), 6.33 (d, *J* = 14.1 Hz, 2H), 4.15 (t, *J* = 7.3 Hz, 2H), 3.23 (t, *J* = 7.1 Hz, 2H), 2.89 – 2.78 (m, 4H), 2.10 – 2.06 (m, 4H), 1.87 (q, *J* = 7.4 Hz, 4H), 1.37 (s, 6H), 1.35 (s, 6H), 1.05 (t, *J* = 7.4 Hz, 6H), 1.04 – 0.99 (m, 2H). **^{13}C NMR** (126 MHz, MeOD) δ 173.0, 147.8, 147.6, 142.1, 141.6, 141.2, 128.5, 126.5, 126.0, 125.4, 122.1, 111.1, 101.3, 49.1, 45.3, 33.8, 26.9, 26.6, 24.0, 20.6, 20.6, 20.4, 10.2.

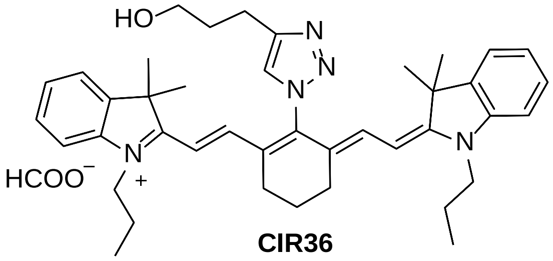

CIR36

^{1}H NMR (500 MHz, MeOD) δ 8.20 (s, 1H), 7.91 (s, 1H), 7.44 (ddd, *J* = 20.8, 7.6, 1.2 Hz, 3H), 7.34 (d, *J* = 8.0 Hz, 2H), 7.28 (td, *J* = 7.5, 0.9 Hz, 2H), 6.82 (d, *J* = 17.5 Hz, 2H), 6.34 (d, *J* = 14.1 Hz, 2H), 4.29 – 4.18 (m, 1H), 4.15 (t, *J* = 7.3 Hz, 2H), 3.05 (d, *J* = 6.2 Hz, 2H), 2.83 (q, *J* = 6.5, 5.4 Hz, 4H), 1.88 (q, *J* = 7.4 Hz, 4H), 1.39 (s, 6H), 1.37 (s, 6H), 1.29 – 1.21 (m, 3H), 1.20 – 1.13 (m, 4H) 1.05 (t, *J* = 7.4 Hz, 6H), 1.02 – 0.84 (m, 2H). **^{13}C NMR** (126 MHz, MeOD) δ 173.0, 147.8, 145.6, 142.1, 141.6, 141.2, 128.5, 126.7, 126.6, 125.4, 122.1, 111.1, 101.4, 66.5, 49.1, 48.1, 45.3, 34.4, 26.9, 26.6, 24.1, 22.3, 20.6, 10.2.

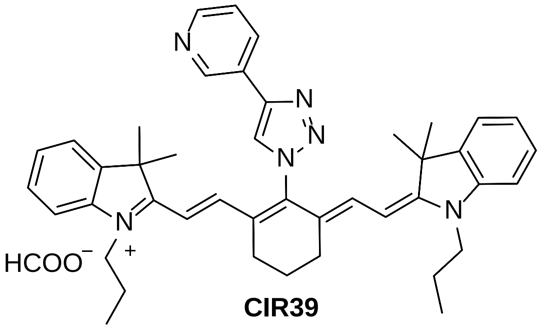

CIR39

^{1}H NMR (500 MHz, MeOD) δ 9.04 (s, 1H), 8.66 (d, *J* = 4.8 Hz, 1H), 8.49 – 8.46 (m, 1H), 7.66 (dd, *J* = 8.0, 4.8 Hz, 1H), 7.45 – 7.38 (m, 4H), 7.36 – 7.32 (m, 2H), 7.25 (td, *J* = 7.5, 0.9 Hz, 2H), 6.91 (d, *J* = 14.1 Hz, 3H), 6.37 (d, *J* = 14.1 Hz, 2H), 4.16 (t, *J* = 7.3 Hz, 2H), 2.92 – 2.84 (m, 2H), 1.88 (q, *J* = 7.4 Hz, 4H), 1.36 (s, 6H), 1.29 (s, 6H), 1.24 – 1.13 (m, 4H), 1.05 (t, *J* = 7.4 Hz, 6H), 0.98 – 0.81 (m, 2H). **^{13}C NMR** (126 MHz, MeOD) δ 173.1, 149.0, 147.3, 146.0, 144.7, 142.1, 141.4, 141.1, 133.7, 128.5, 126.4, 125.6, 125.5, 122.1, 111.2, 101.5, 49.1, 45.4, 26.6, 24.1, 20.6, 10.2.

NMR spectra

Compound 1

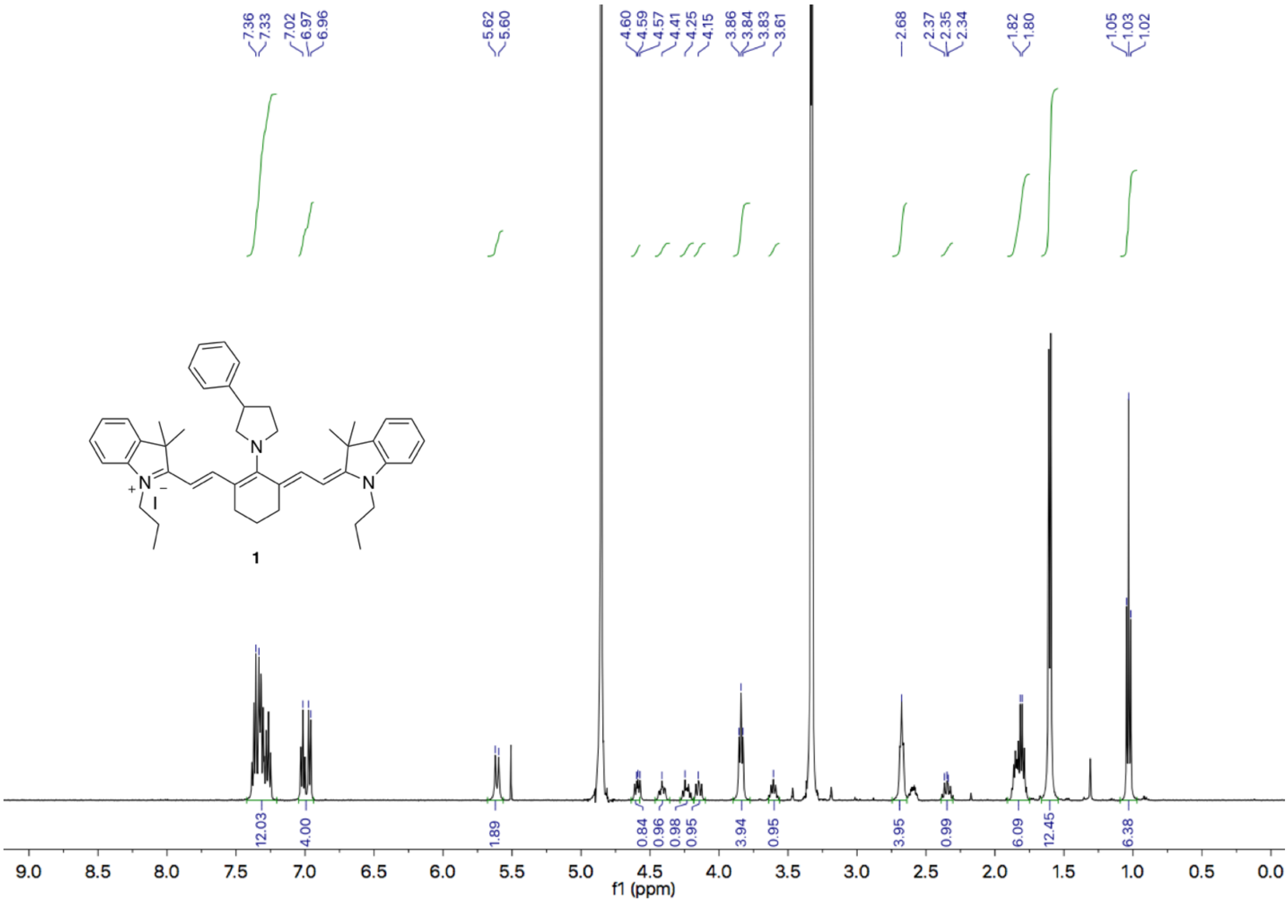

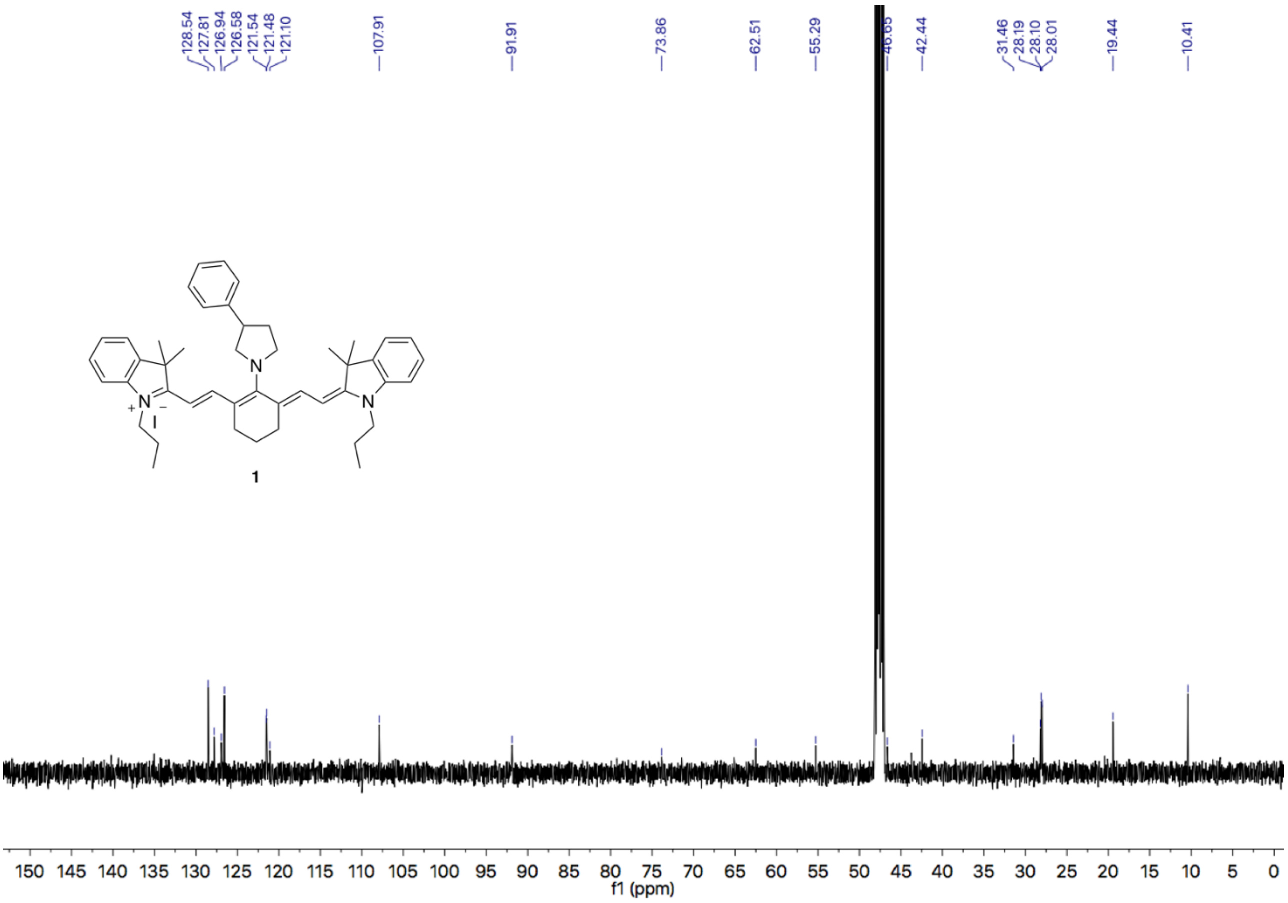

Compound 2

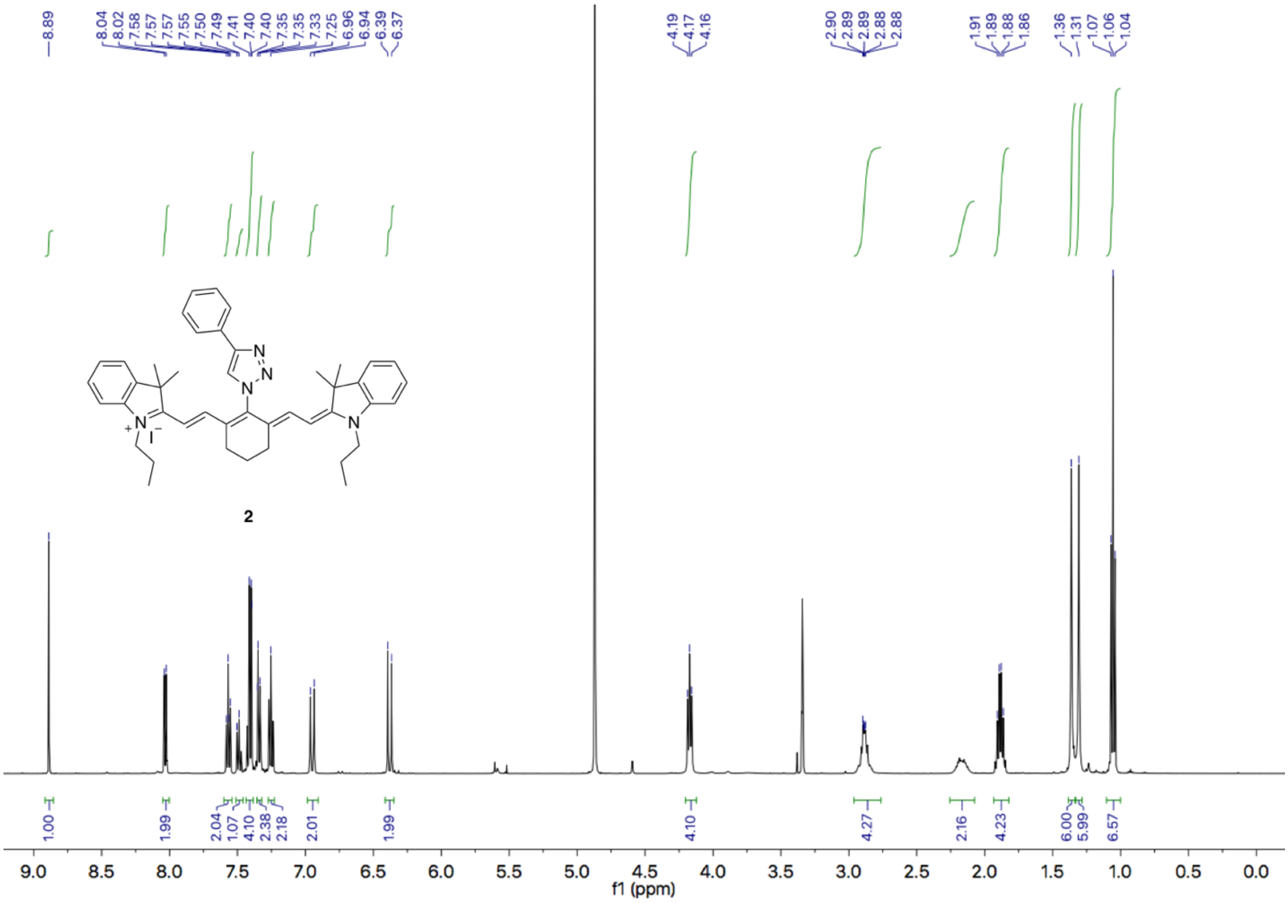
8.89
8.04 8.02 7.58 7.57 7.57 7.55 7.50 7.49 7.41 7.40 7.40 7.35 7.35 7.33 7.25 6.96 6.94 6.39 6.37
4.19 4.17 4.16
2.90 2.89 2.89 2.88 2.88
1.91 1.89 1.88 1.86
1.36 1.31 1.07 1.06 1.04
1.00 1.99 2.04 1.07 4.10 2.38 2.18 2.01 1.99 4.10 4.27 2.16 4.23 6.00 5.99 6.57
2
f1 (ppm)

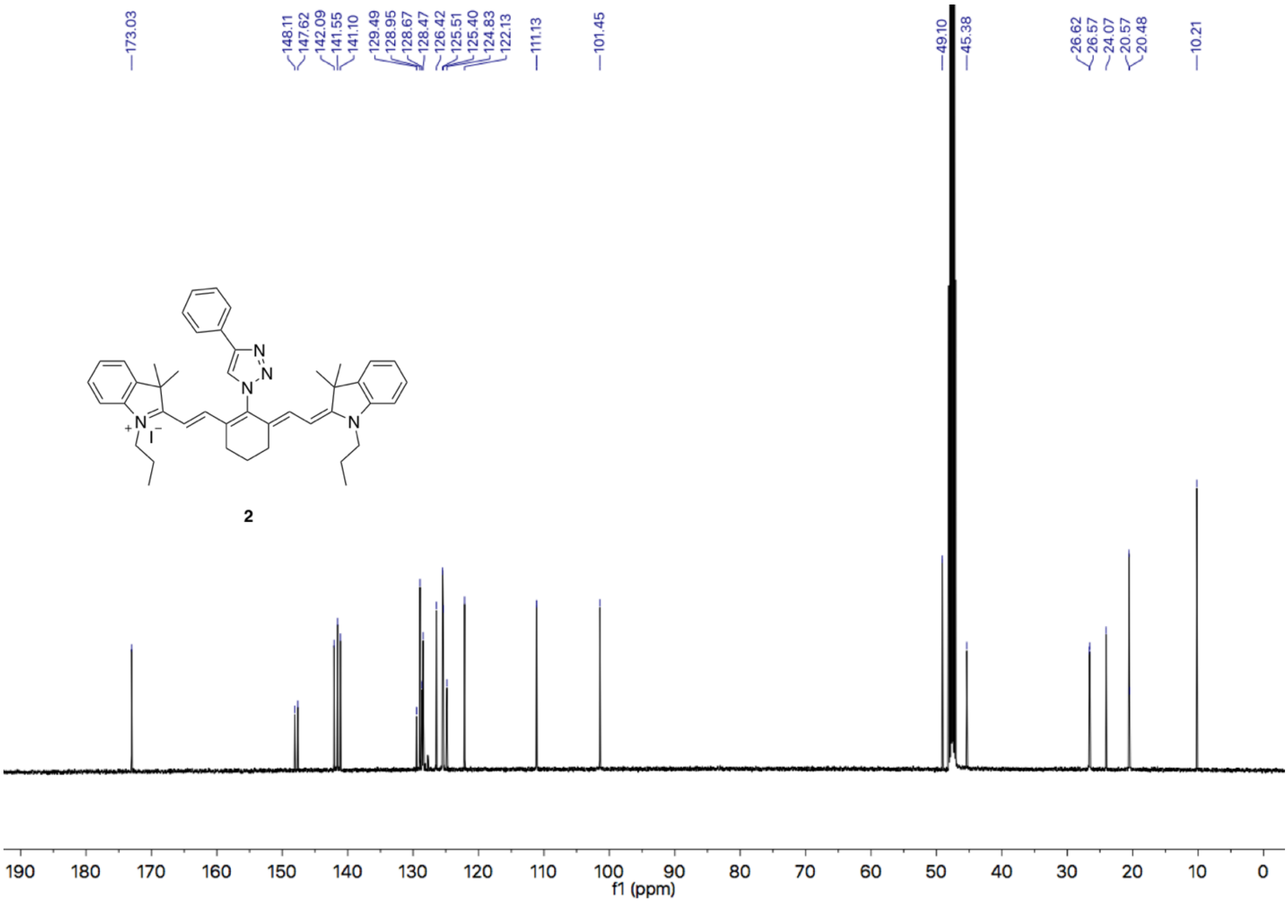
173.03
148.11 147.62 142.09 141.55 141.10 129.49 128.95 128.67 128.47 126.42 125.51 125.40 124.83 122.13 111.13 101.45
49.10 45.38
26.62 26.57 24.07 20.57 20.48 10.21
2
f1 (ppm)

Compound IR780-COOH

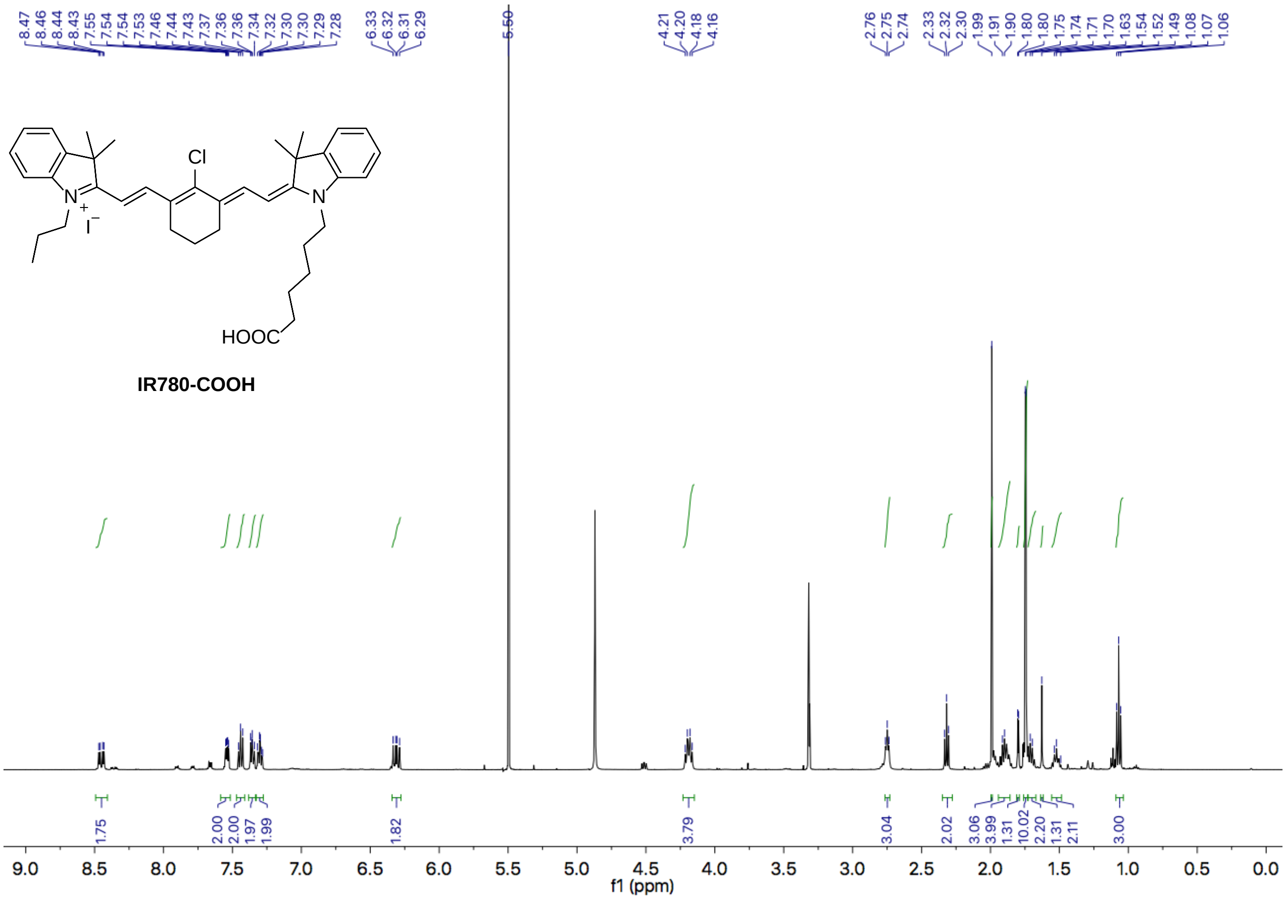

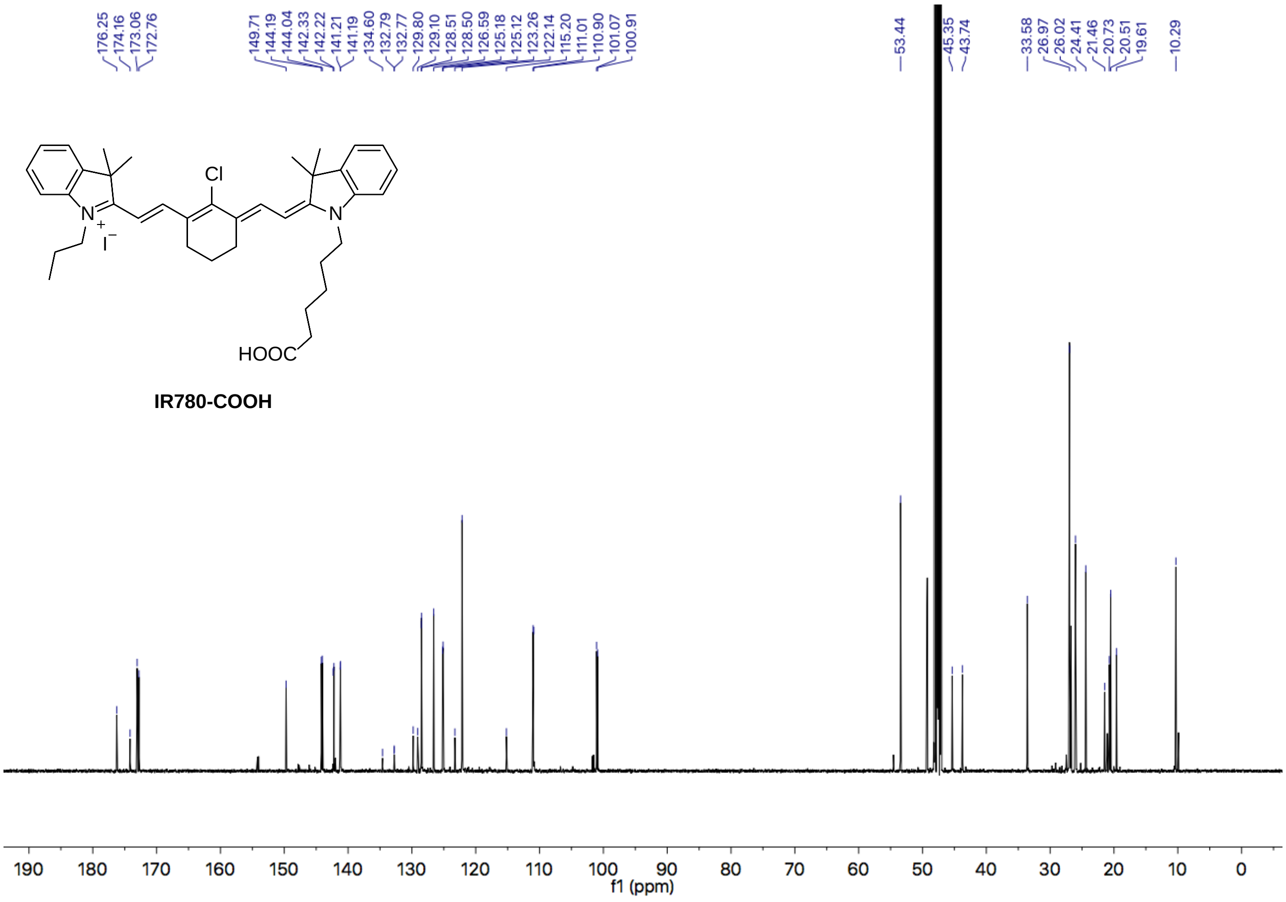

Compound CIR38-COOH

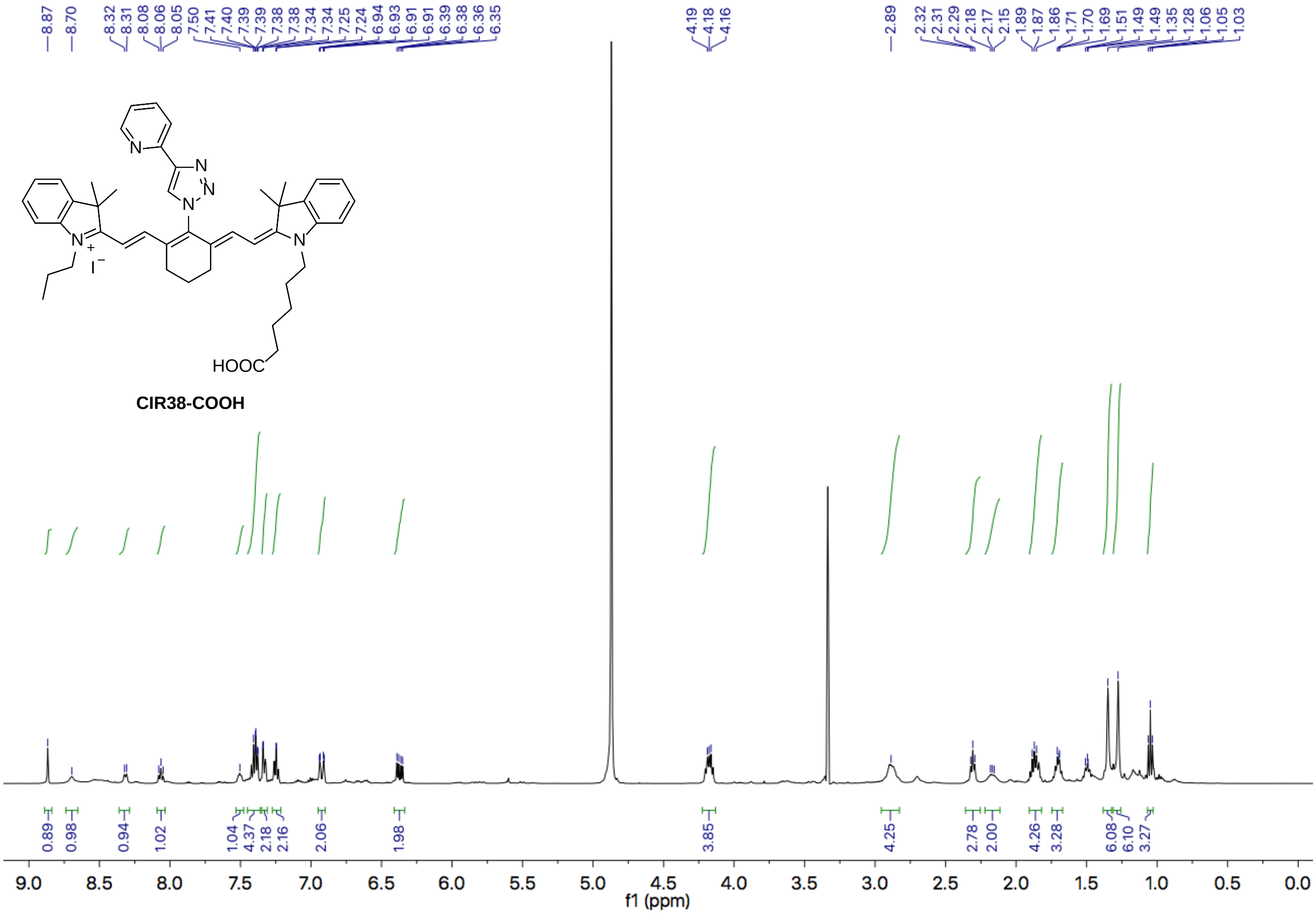

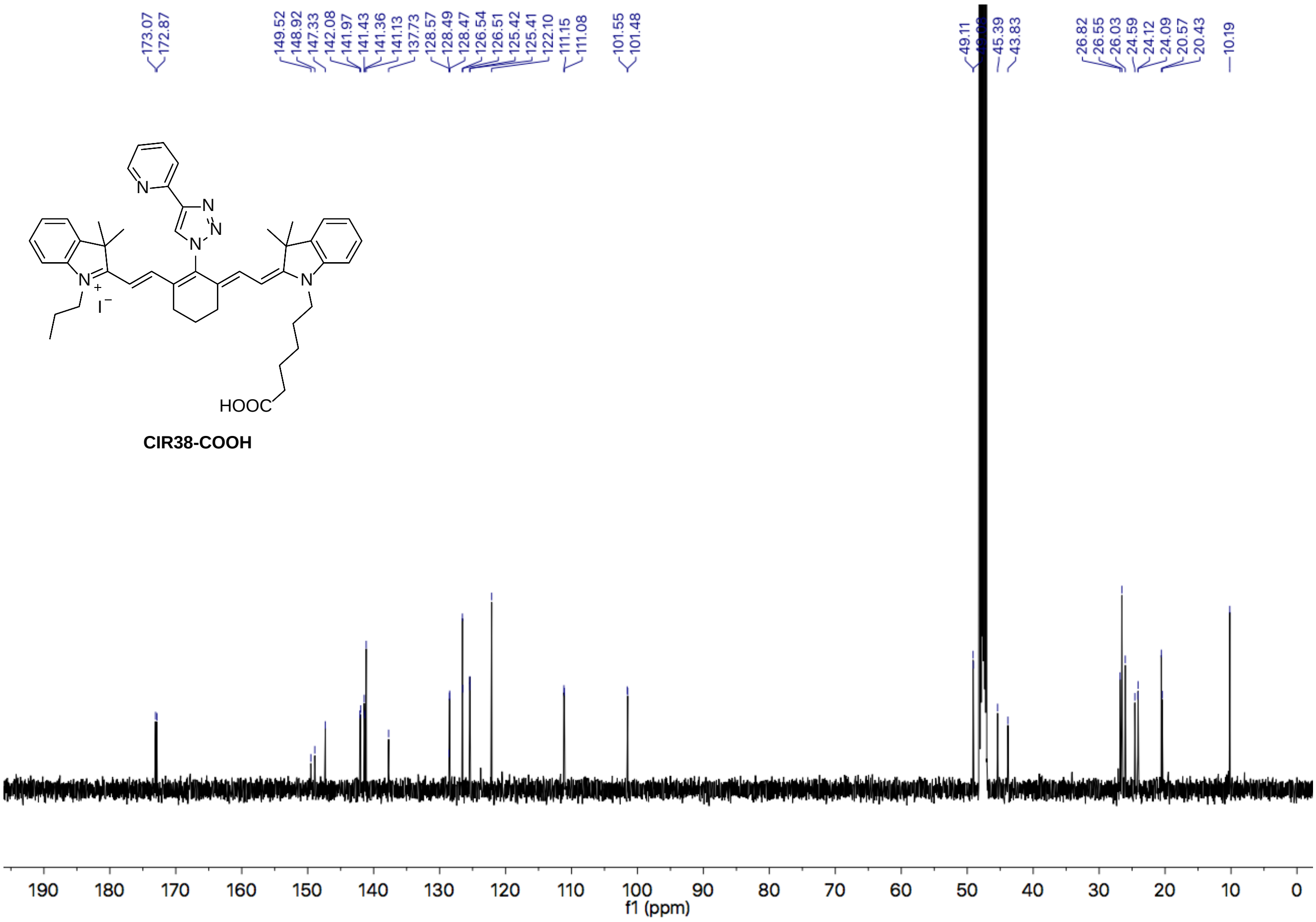

Compound CIR38M

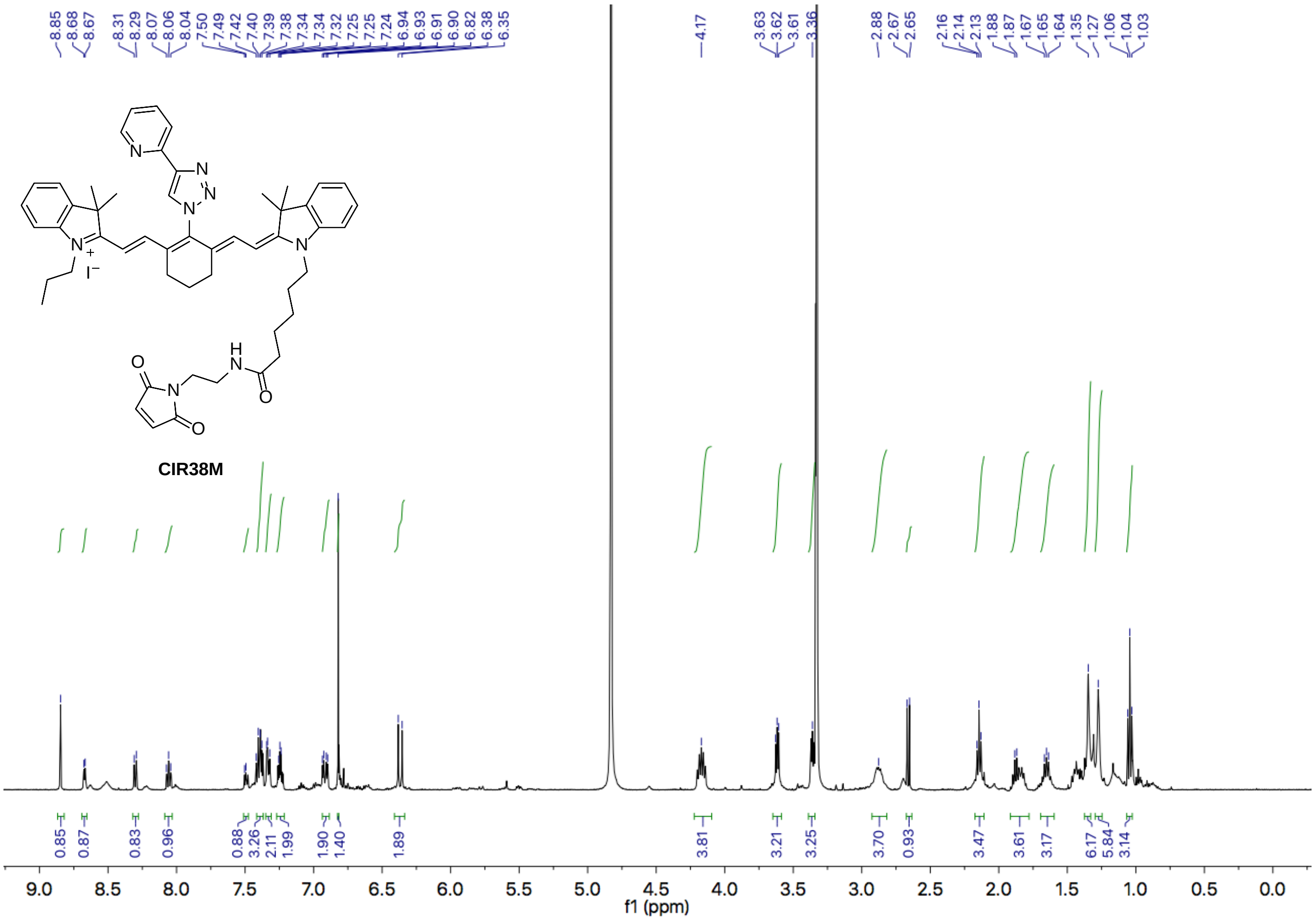

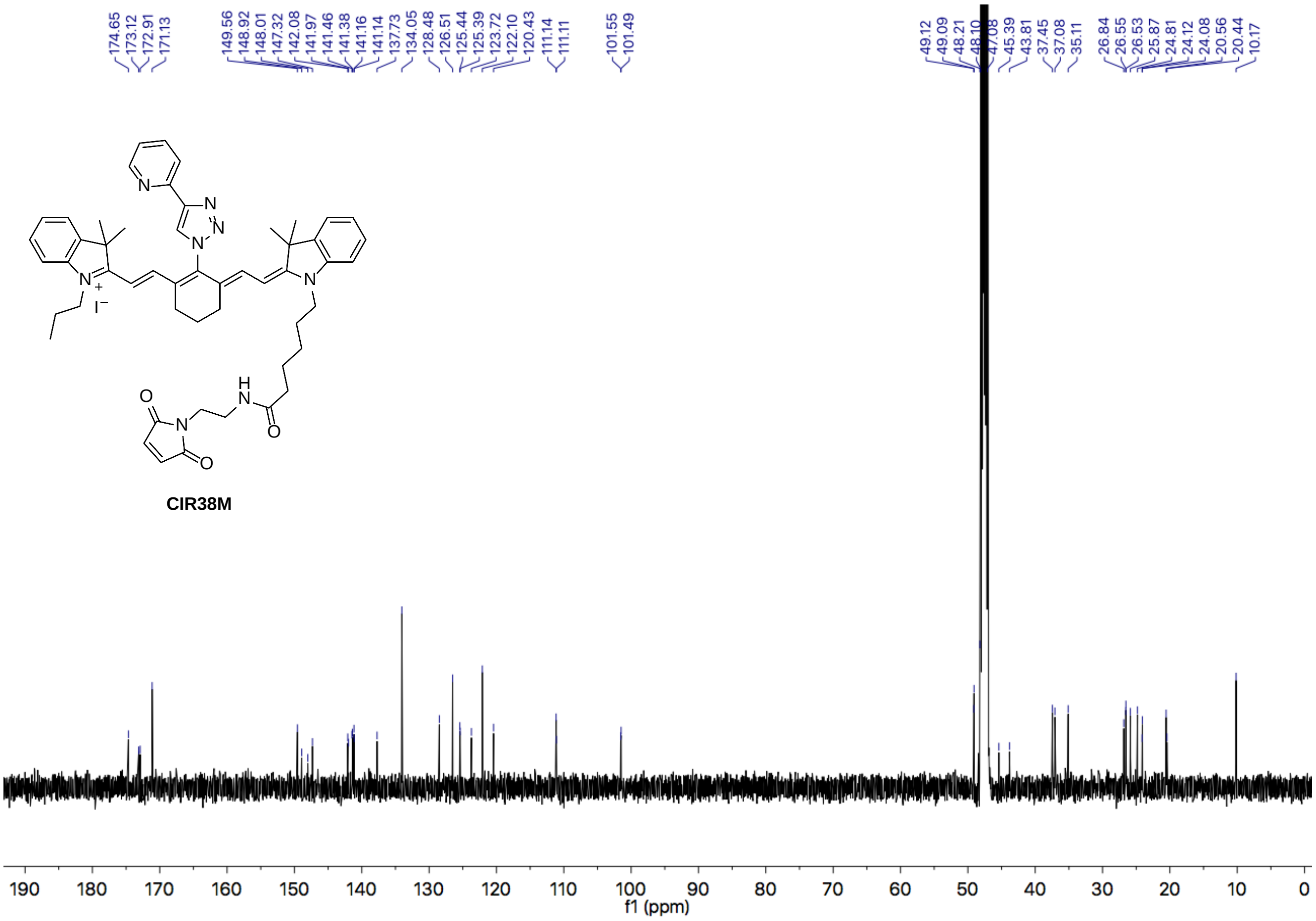

CIR library (representative subset)

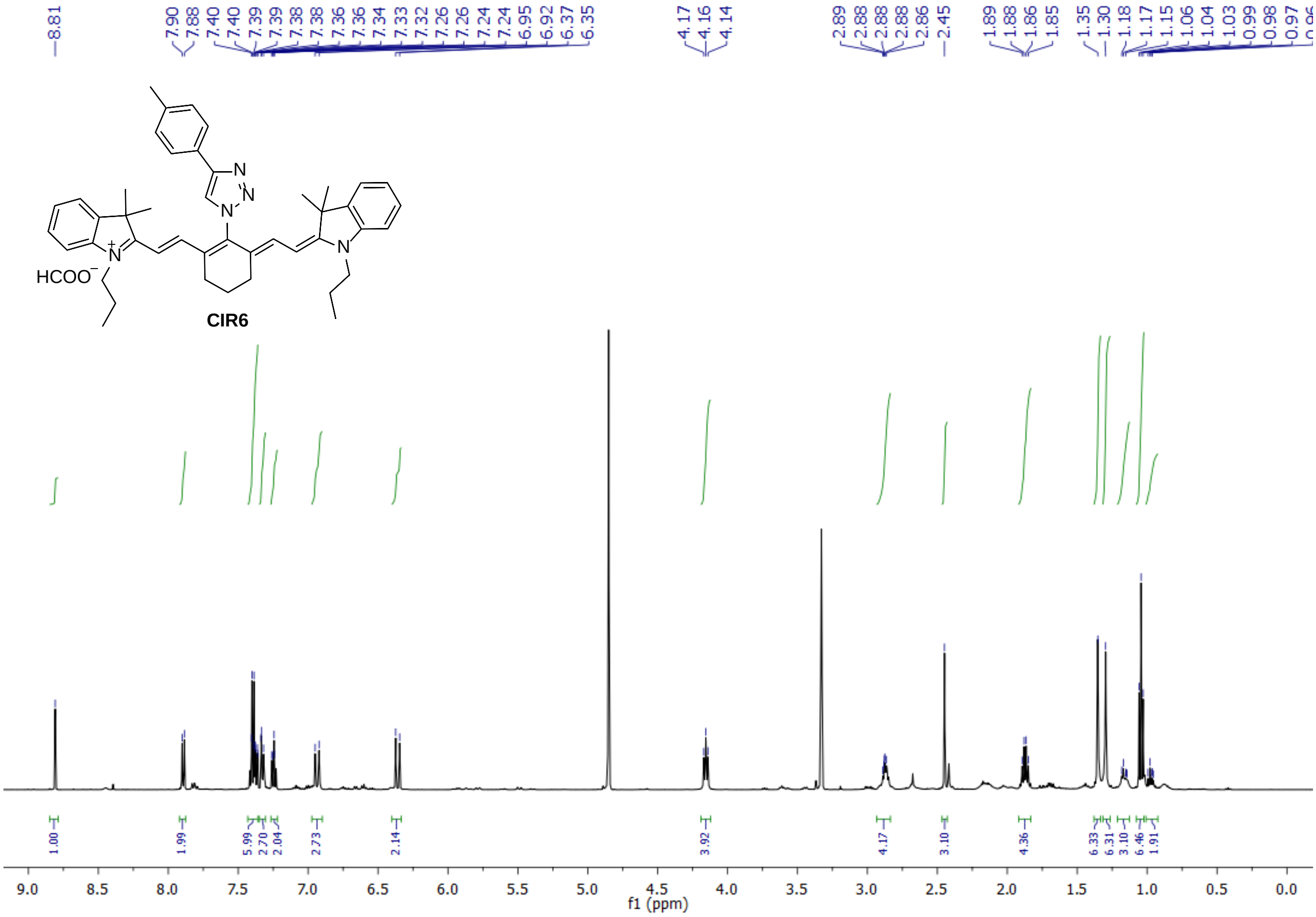

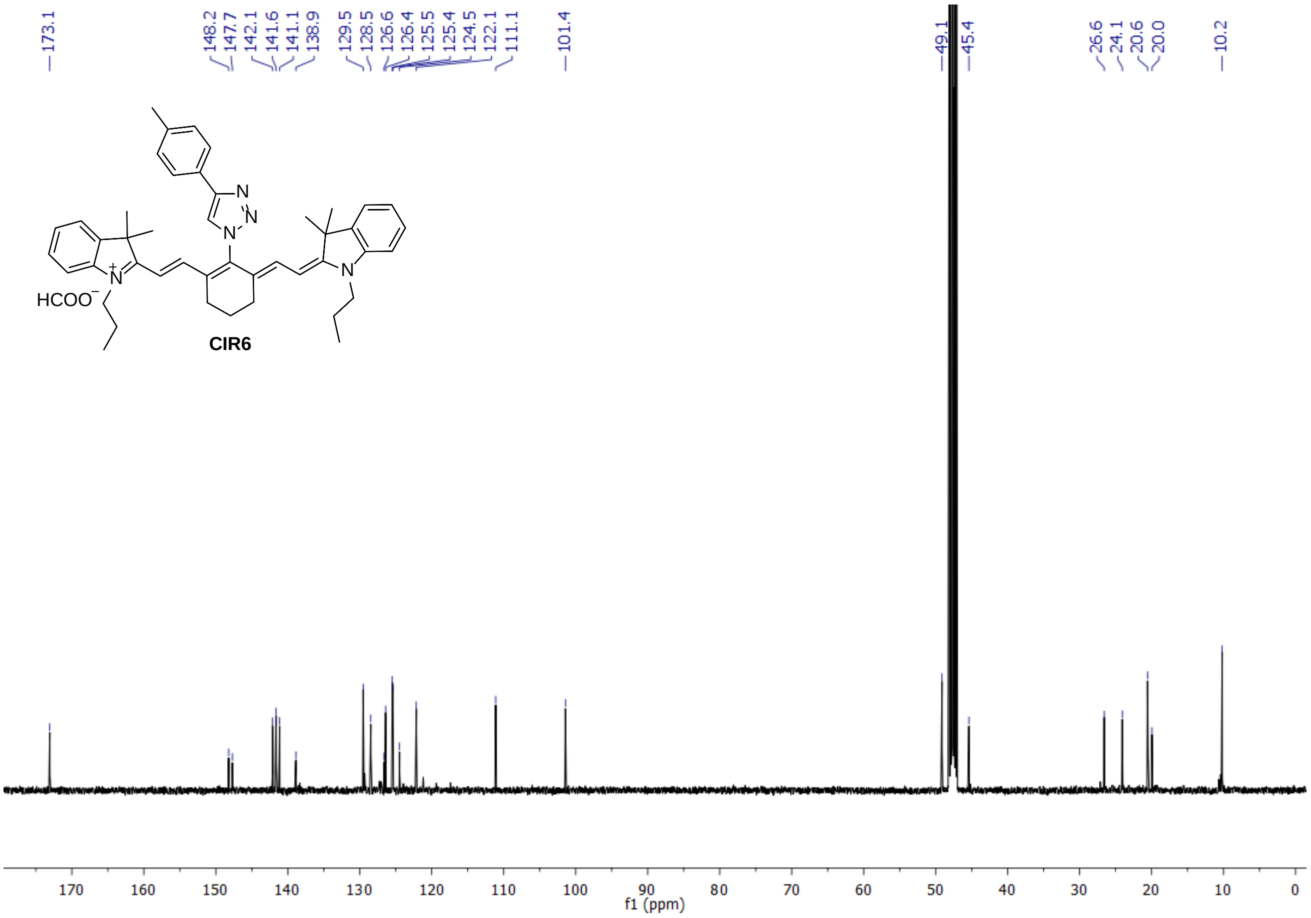

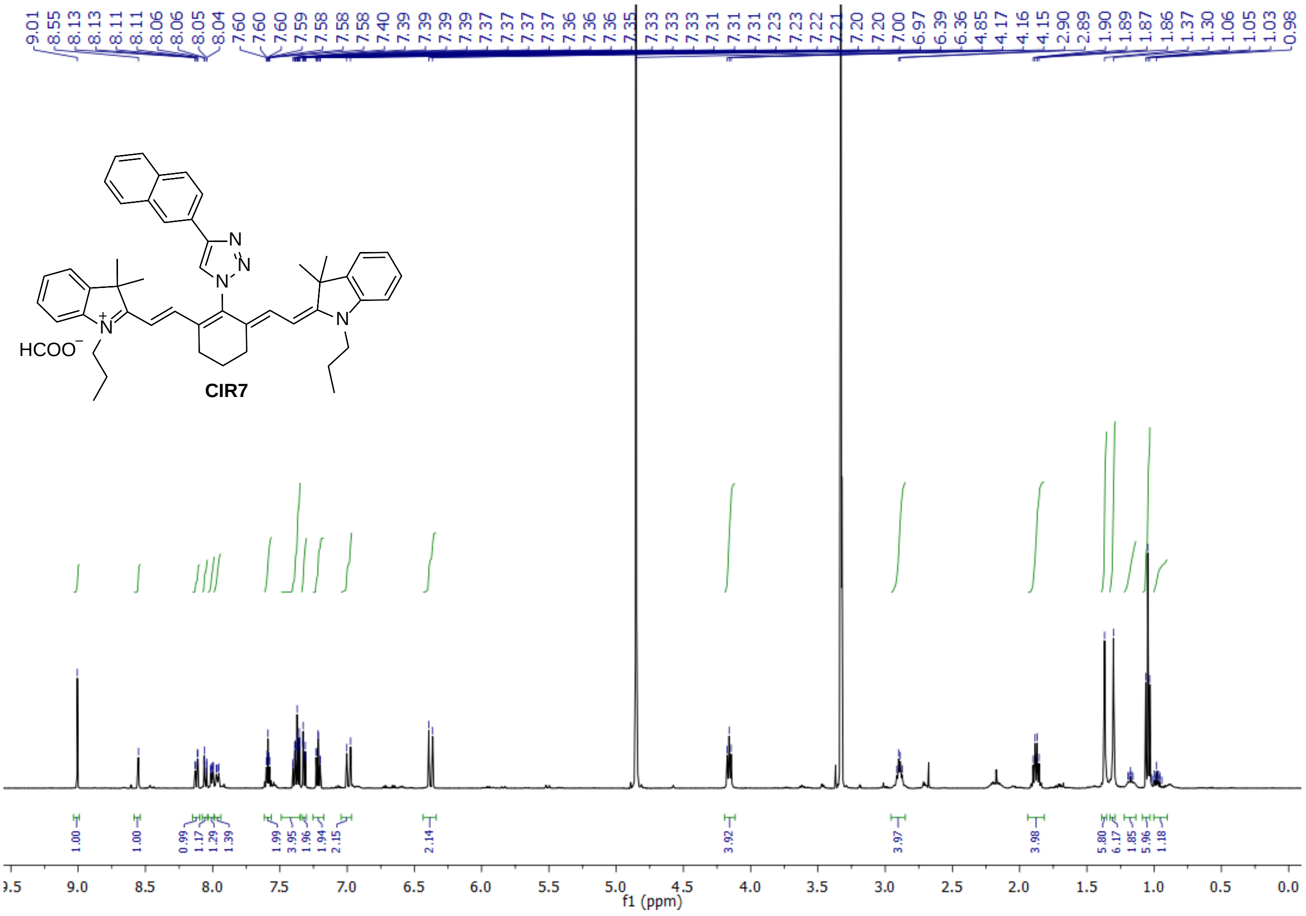
9.01 8.55 8.13 8.13 8.11 8.11 8.06 8.06 8.05 8.04 7.60 7.60 7.60 7.59 7.58 7.58 7.58 7.40 7.39 7.39 7.39 7.39 7.37 7.37 7.37 7.37 7.36 7.36 7.36 7.35 7.33 7.33 7.33 7.31 7.31 7.31 7.23 7.23 7.22 7.21 7.20 7.20 7.00 6.97 6.39 6.36 4.85 4.17 4.16 4.15 2.90 2.89 1.90 1.89 1.87 1.86 1.37 1.30 1.06 1.05 1.03 0.98
HCOO⁻
CIR7
1.00 1.00 0.99 1.17 1.29 1.39 1.99 3.95 1.96 1.94 2.15 2.14 3.92 3.97 3.98 5.80 6.17 1.85 5.96 1.18
f1 (ppm)

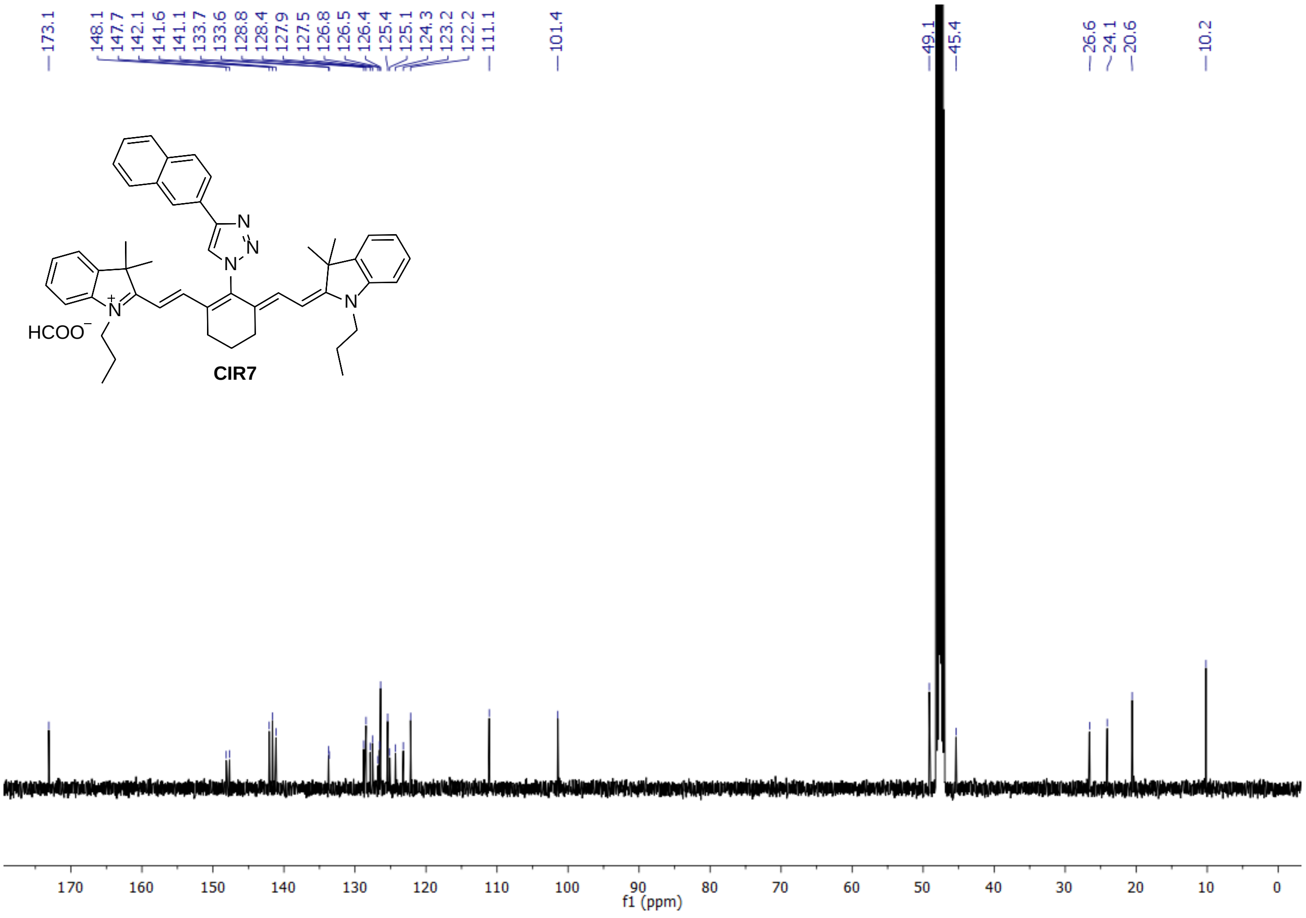
173.1 148.1 147.7 142.1 141.6 141.1 133.7 133.6 128.8 128.4 127.9 127.5 126.8 126.5 126.4 125.4 125.1 124.3 123.2 122.2 111.1 101.4 49.1 45.4 26.6 24.1 20.6 10.2
HCOO⁻
CIR7
f1 (ppm)

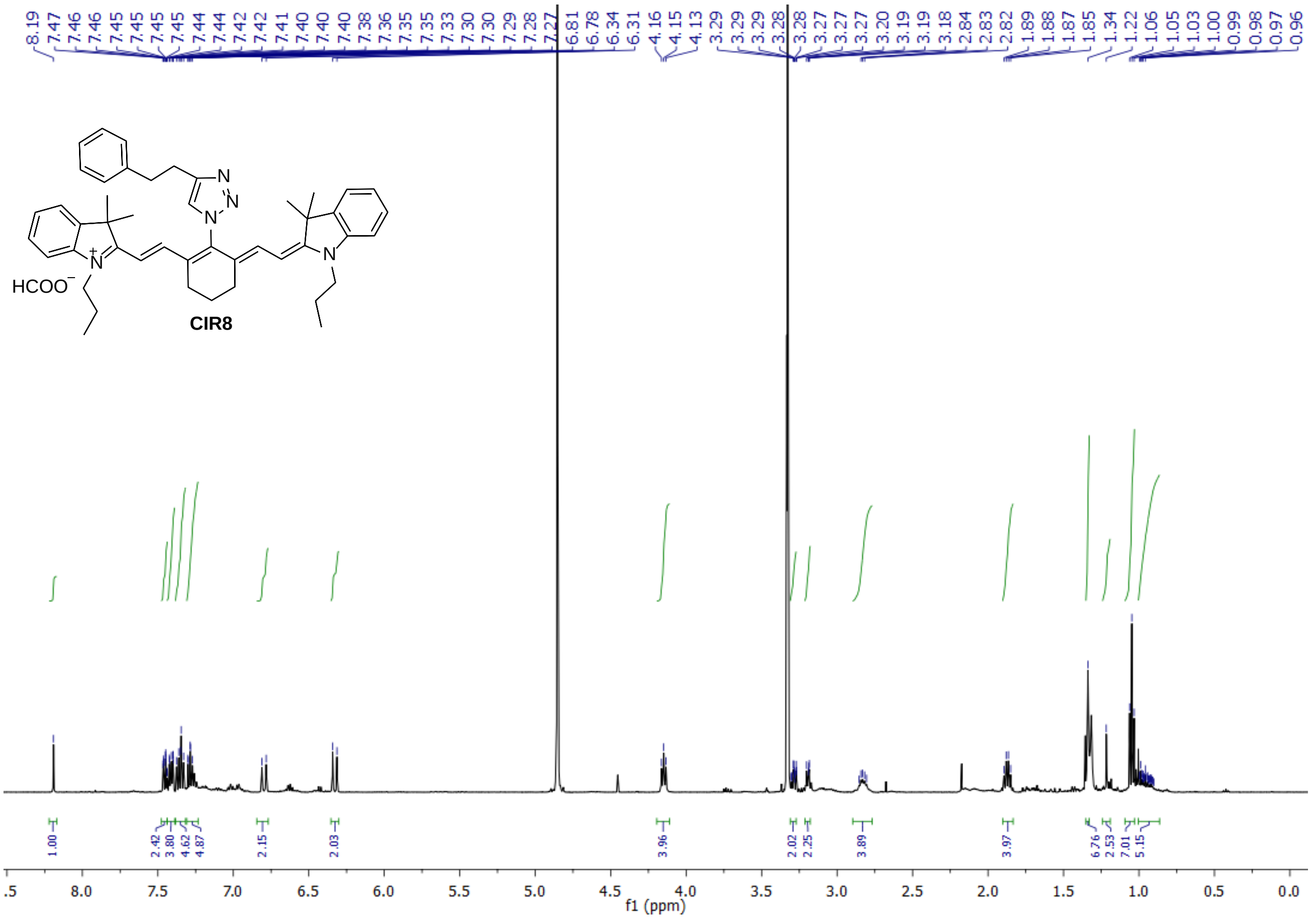
HCOO⁻
CIR8
f1 (ppm)

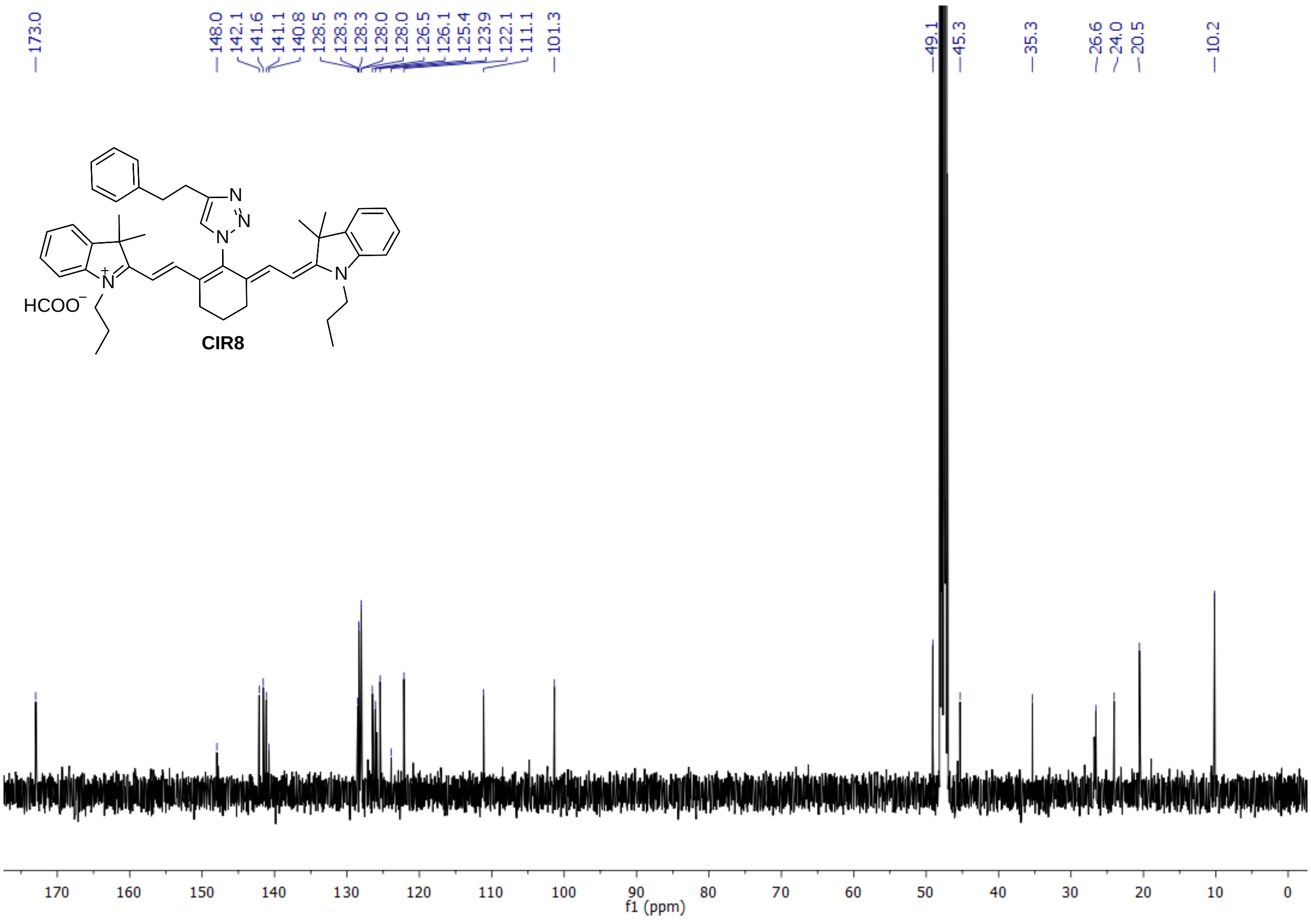
HCOO⁻
CIR8
f1 (ppm)

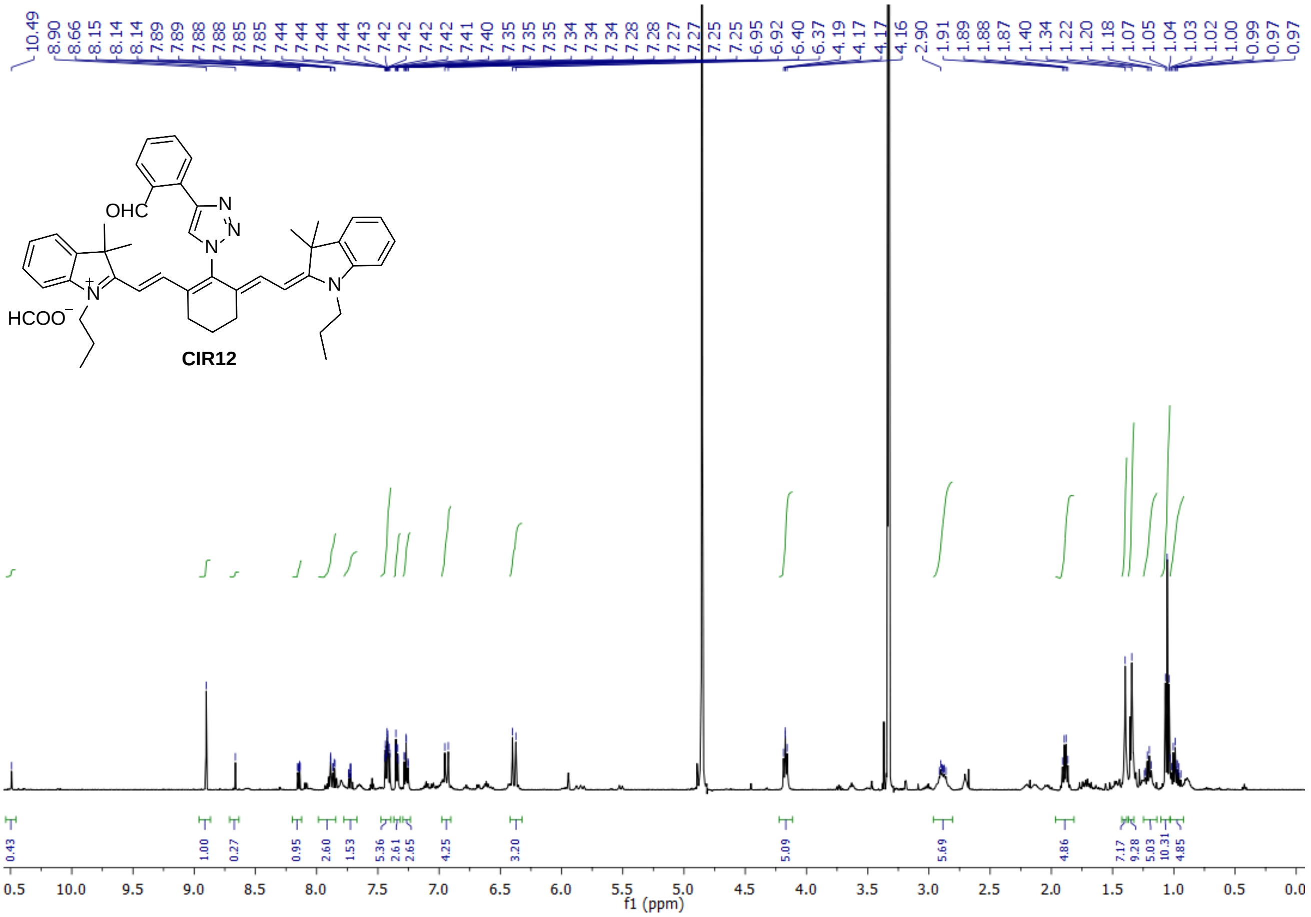
OHC
HCOO⁻
CIR12
f1 (ppm)

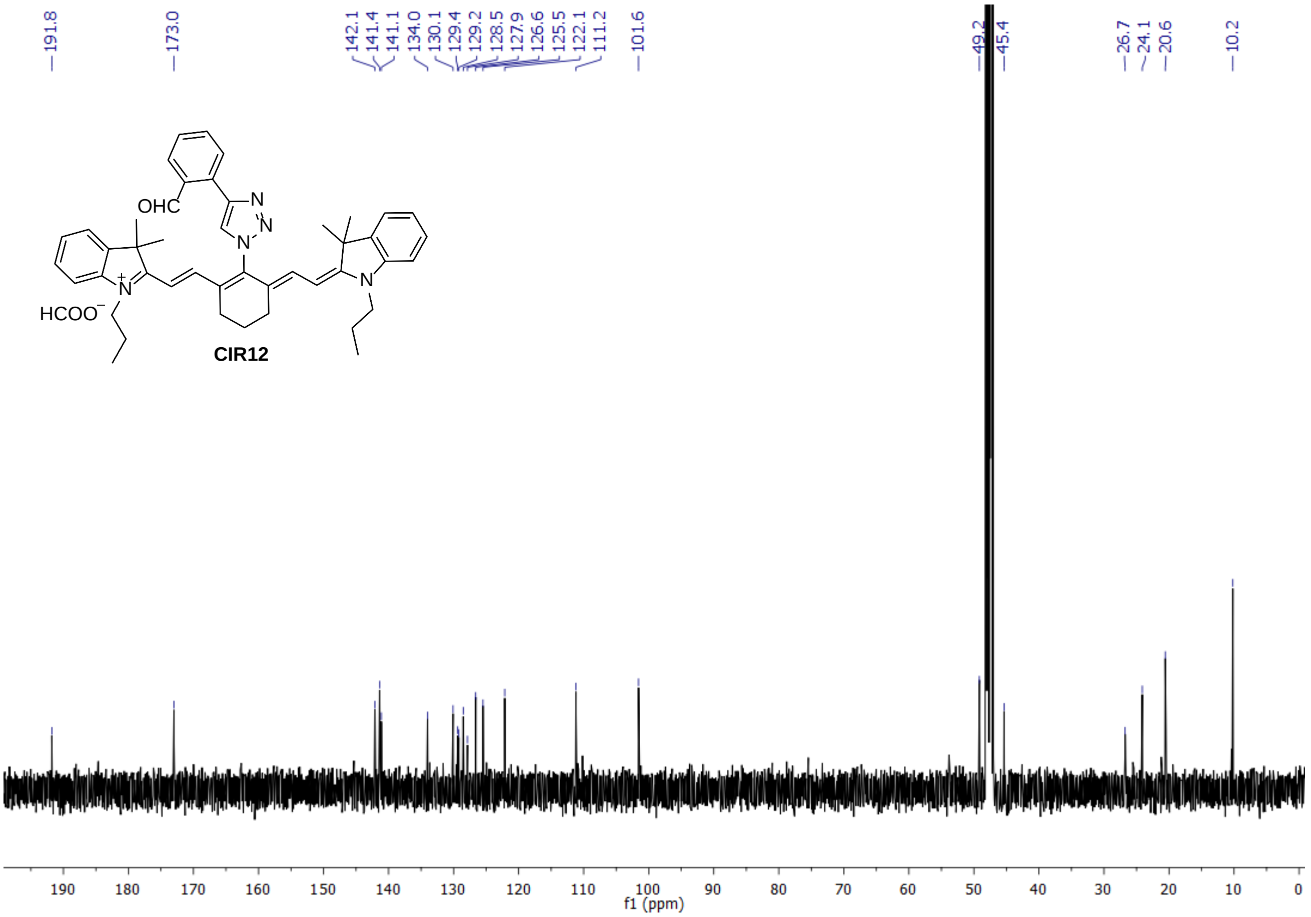
OHC
HCOO⁻
CIR12
f1 (ppm)

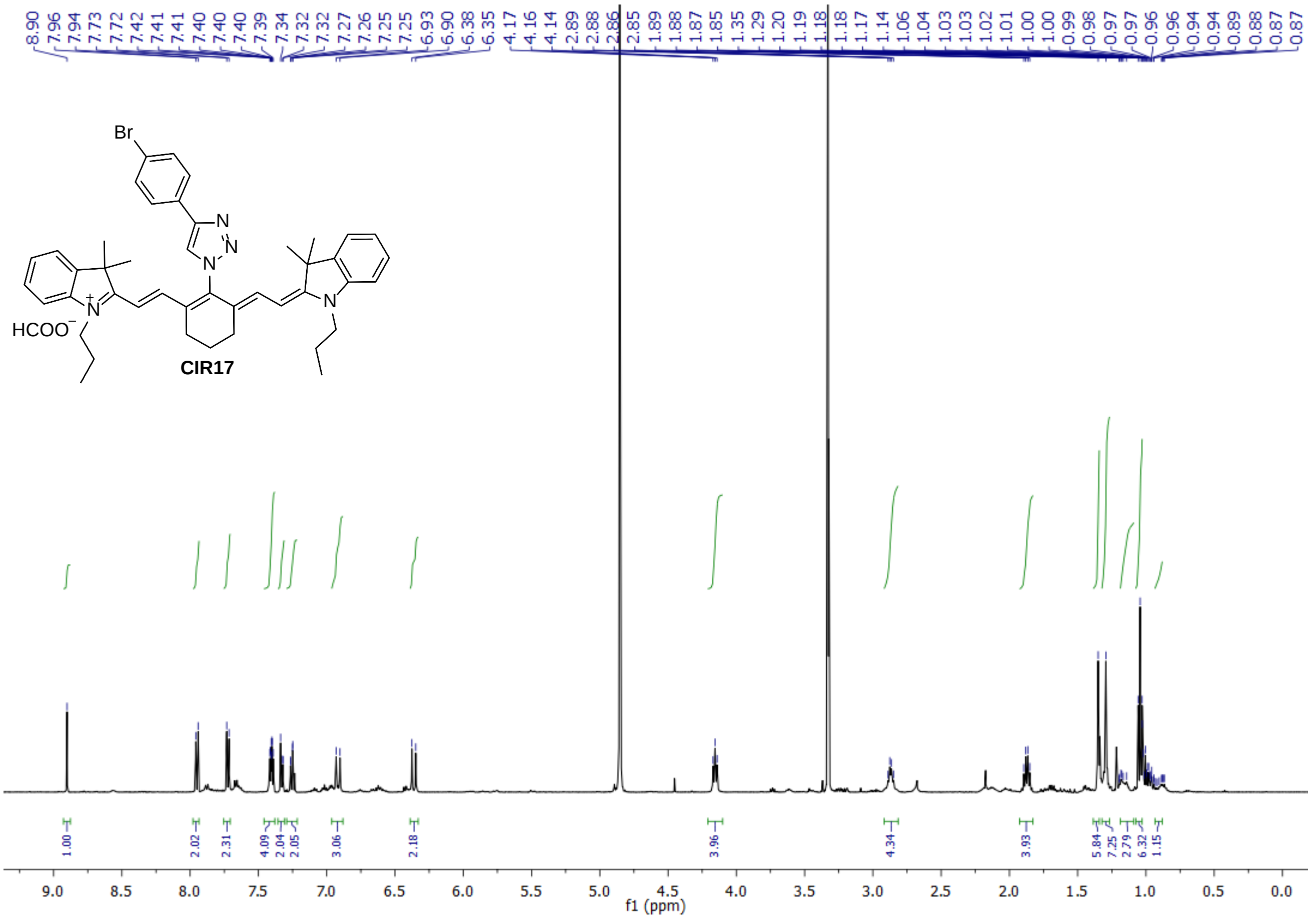
8.90 7.96 7.94 7.73 7.72 7.42 7.41 7.41 7.40 7.40 7.40 7.39 7.34 7.32 7.32 7.27 7.26 7.25 7.25 6.93 6.90 6.38 6.35 4.17 4.16 4.14 2.89 2.88 2.86 2.85 1.89 1.88 1.87 1.85 1.35 1.29 1.20 1.19 1.18 1.18 1.17 1.14 1.06 1.04 1.03 1.03 1.02 1.01 1.00 1.00 0.99 0.98 0.97 0.97 0.96 0.96 0.94 0.94 0.89 0.88 0.87 0.87
Br
N
N
N
N
N
HCOO⁻
CIR17
1.00 2.02 2.31 4.09 2.04 2.05 3.06 2.18 3.96 4.34 3.93 5.84 7.25 2.79 6.32 1.15
9.0 8.5 8.0 7.5 7.0 6.5 6.0 5.5 5.0 4.5 4.0 3.5 3.0 2.5 2.0 1.5 1.0 0.5 0.0
f1 (ppm)

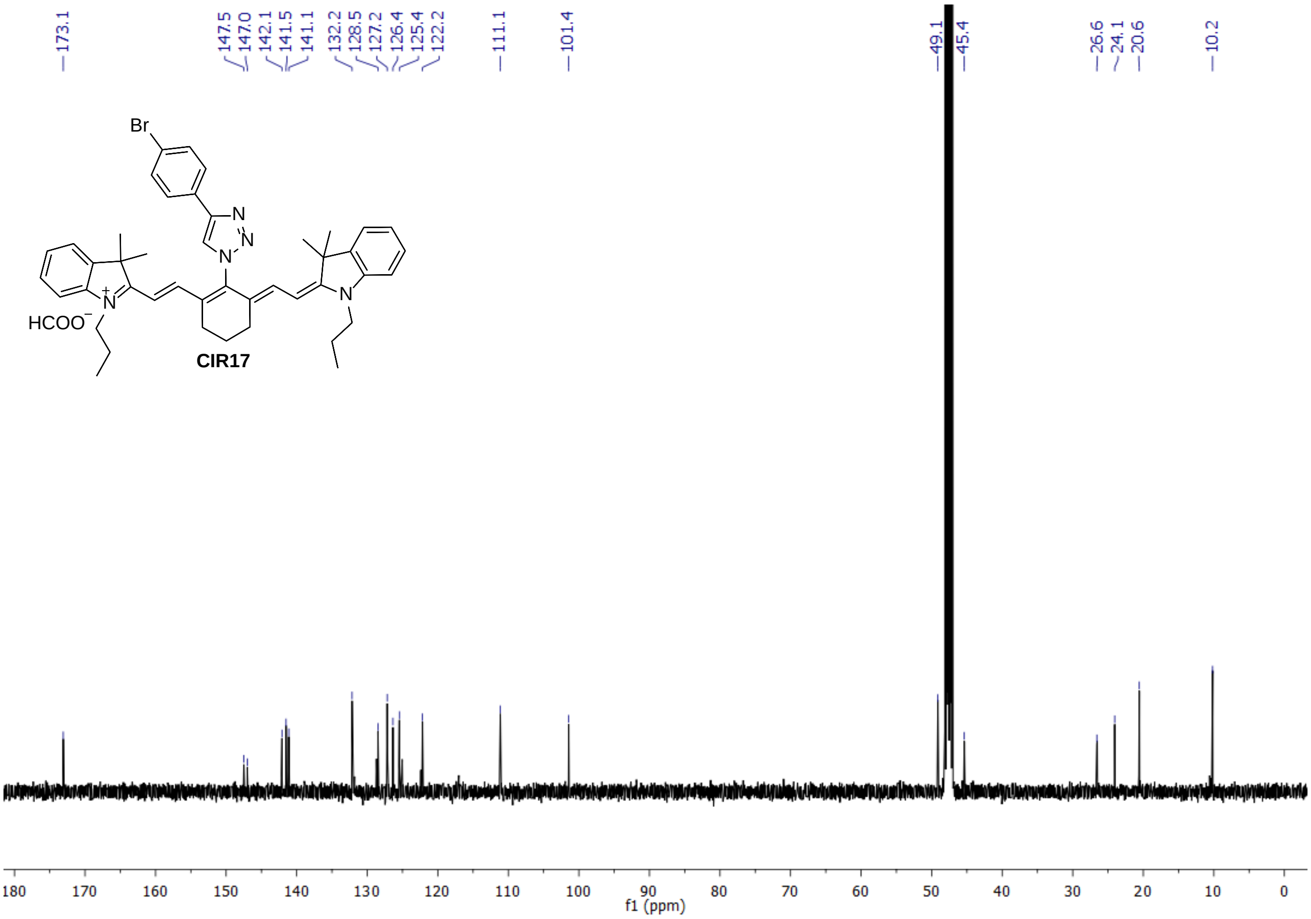
173.1
147.5 147.0 142.1 141.5 141.1 132.2 128.5 127.2 126.4 125.4 122.2
111.1
101.4
49.1 45.4
26.6 24.1 20.6
10.2
Br
N
N
N
N
N
HCOO⁻
CIR17
180 170 160 150 140 130 120 110 100 90 80 70 60 50 40 30 20 10 0
f1 (ppm)

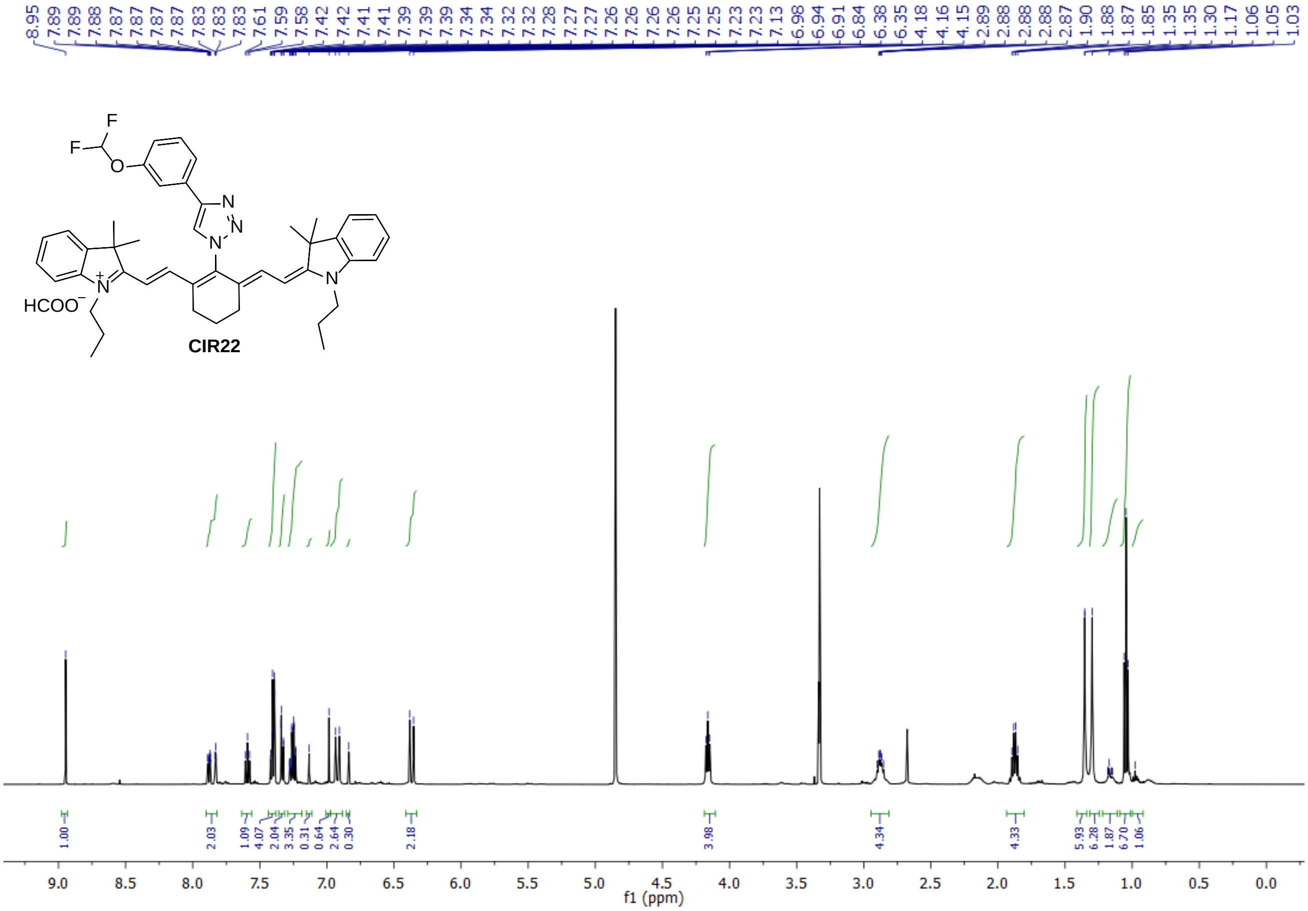
8.95 7.89 7.89 7.88 7.87 7.87 7.87 7.87 7.83 7.83 7.83 7.61 7.59 7.58 7.42 7.42 7.41 7.41 7.39 7.39 7.39 7.34 7.34 7.32 7.32 7.28 7.27 7.27 7.26 7.26 7.26 7.26 7.25 7.25 7.23 7.23 7.13 6.98 6.94 6.91 6.84 6.38 6.35 4.18 4.16 4.15 2.89 2.88 2.88 2.88 2.87 1.90 1.88 1.87 1.85 1.35 1.35 1.30 1.17 1.06 1.05 1.03
F
F
O
N
N
N
N
N
HCOO−
CIR22
1.00 2.03 1.09 4.07 2.04 3.35 0.31 0.64 2.64 0.30 2.18 3.98 4.34 4.33 5.93 6.28 1.87 6.70 1.06
9.0 8.5 8.0 7.5 7.0 6.5 6.0 5.5 5.0 4.5 4.0 3.5 3.0 2.5 2.0 1.5 1.0 0.5 0.0
f1 (ppm)

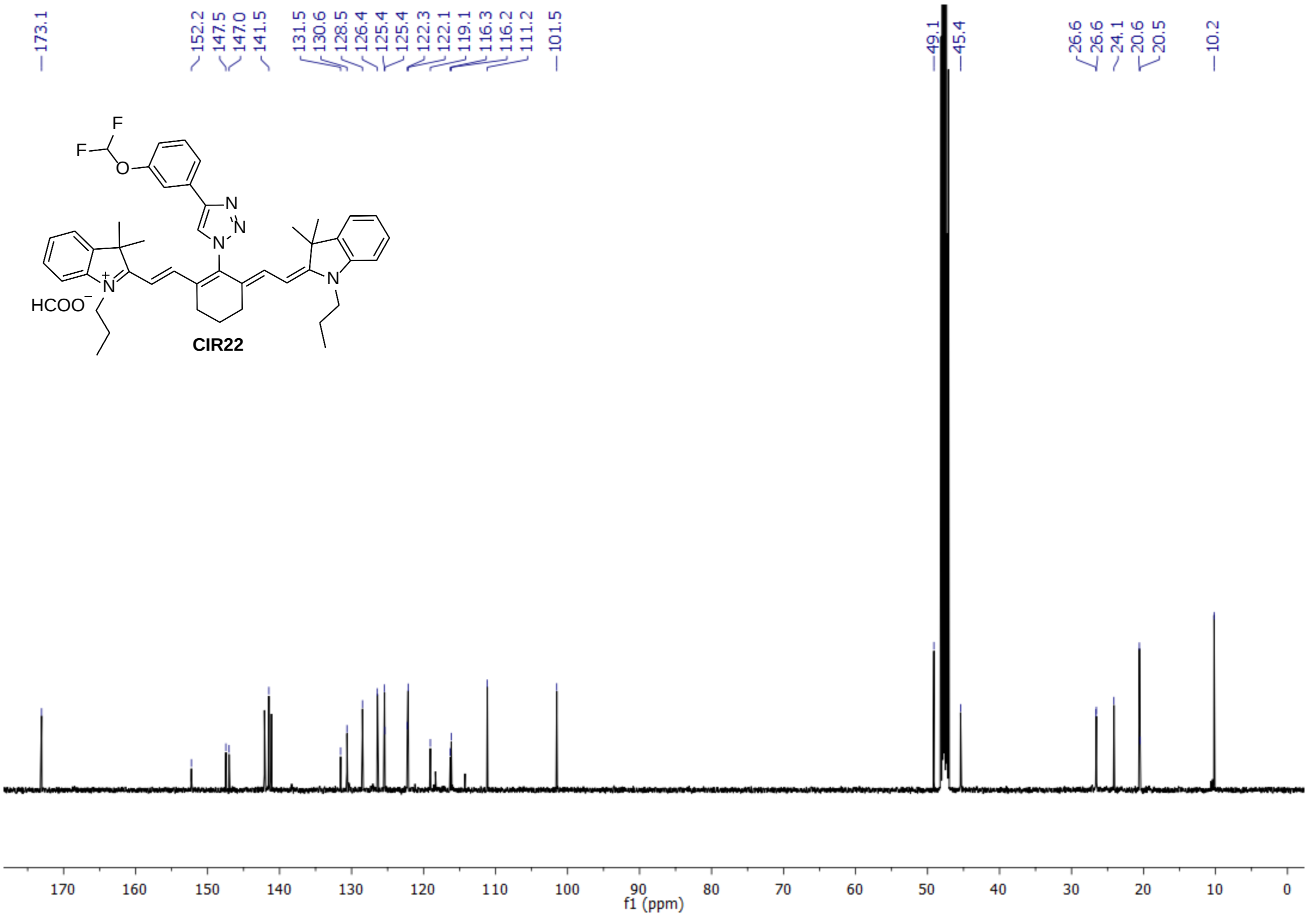
173.1 152.2 147.5 147.0 141.5 131.5 130.6 128.5 126.4 125.4 125.4 122.3 122.1 119.1 116.3 116.2 111.2 101.5 49.1 45.4 26.6 26.6 24.1 20.6 20.5 10.2
F
F
O
N
N
N
N
N
HCOO−
CIR22
170 160 150 140 130 120 110 100 90 80 70 60 50 40 30 20 10 0
f1 (ppm)

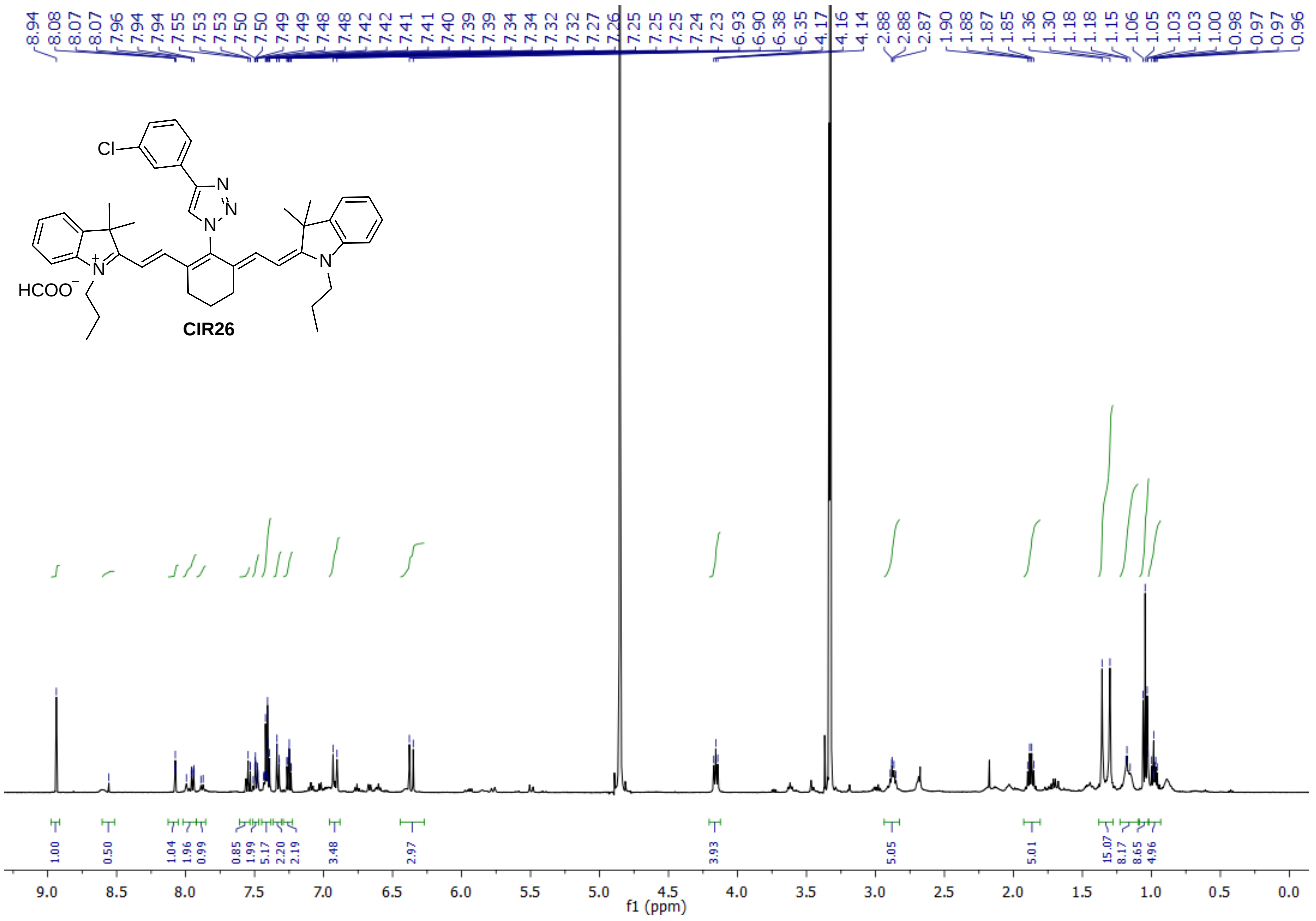
8.94 8.08 8.07 8.07 7.96 7.94 7.94 7.55 7.53 7.53 7.50 7.50 7.49 7.49 7.48 7.48 7.42 7.42 7.41 7.41 7.40 7.39 7.39 7.34 7.34 7.32 7.32 7.27 7.26 7.25 7.25 7.25 7.24 7.23 6.93 6.90 6.38 6.35 4.17 4.16 4.14 2.88 2.88 2.87 1.90 1.88 1.87 1.85 1.36 1.30 1.18 1.18 1.15 1.06 1.05 1.03 1.03 1.00 0.98 0.97 0.97 0.96
Cl
N
N
N
N
N
HCOO−
CIR26
1.00
0.50
1.04
1.96
0.99
0.85
1.99
5.17
2.20
2.19
3.48
2.97
3.93
5.05
5.01
15.07
8.17
8.65
4.96
f1 (ppm)

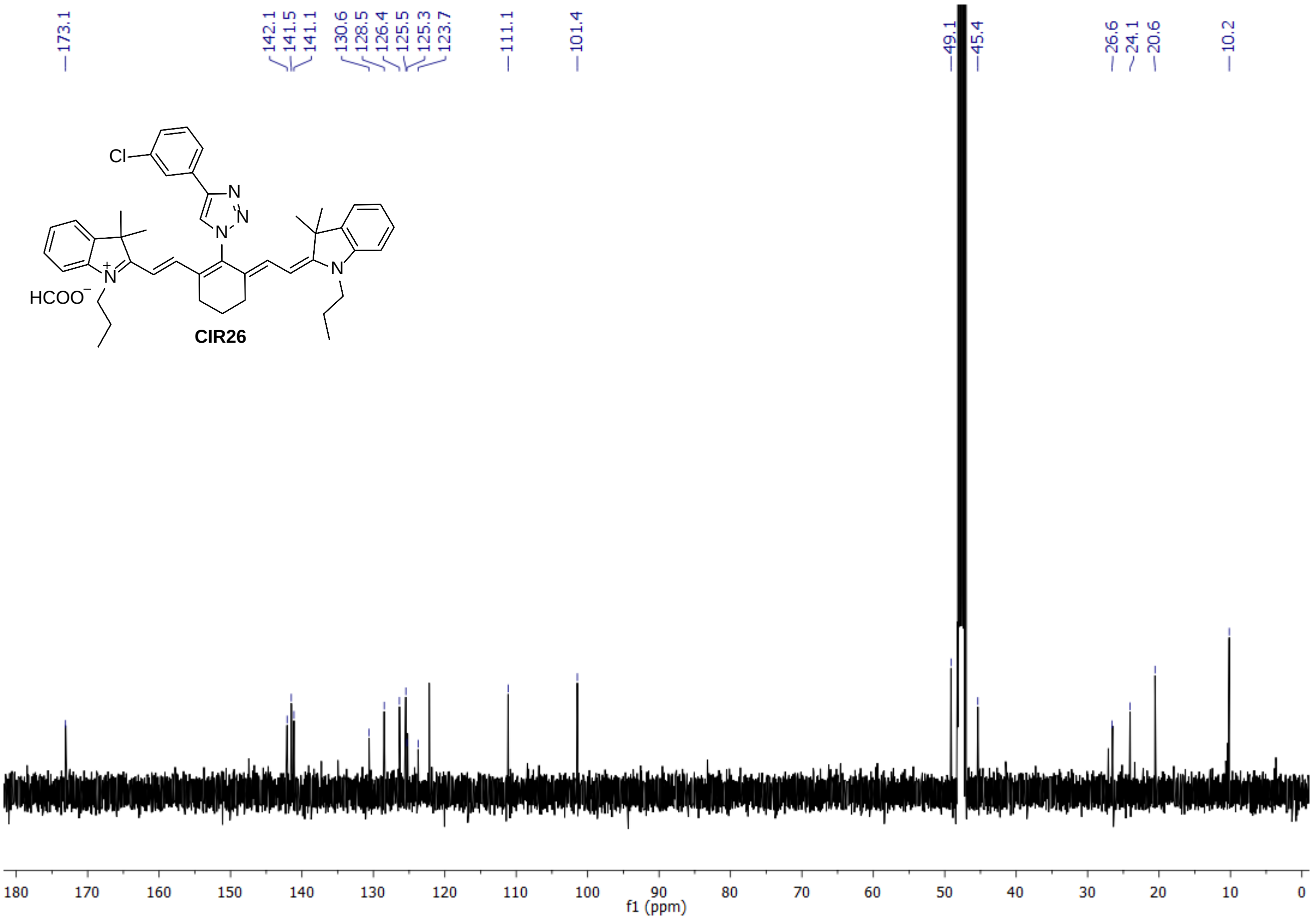
173.1
142.1 141.5 141.1
130.6 128.5 126.4 125.5 125.3 123.7
111.1
101.4
49.1
45.4
26.6 24.1 20.6
10.2
Cl
N
N
N
N
N
HCOO−
CIR26
f1 (ppm)

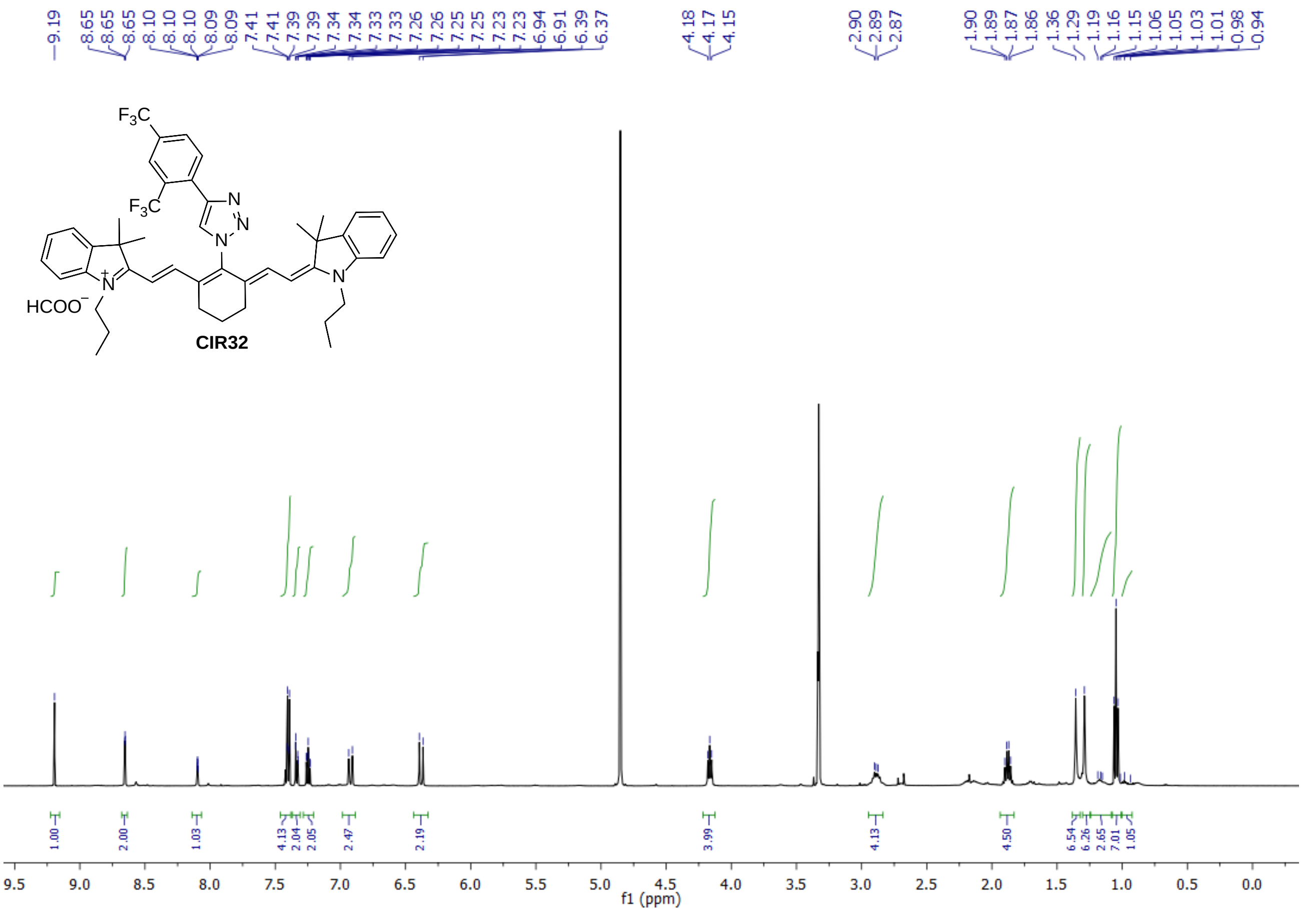
9.19 8.65 8.65 8.65 8.10 8.10 8.09 8.09 7.41 7.41 7.39 7.39 7.34 7.34 7.33 7.33 7.26 7.26 7.25 7.25 7.23 7.23 6.94 6.91 6.39 6.37
4.18 4.17 4.15
2.90 2.89 2.87
1.90 1.89 1.87 1.86 1.36 1.29 1.19 1.16 1.15 1.06 1.05 1.03 1.01 0.98 0.94
F_3C
F_3C
HCOO⁻
CIR32
1.00 2.00 1.03 4.13 2.04 2.05 2.47 2.19 3.99 4.13 4.50 6.54 6.26 2.65 7.01 1.05
9.5 9.0 8.5 8.0 7.5 7.0 6.5 6.0 5.5 5.0 4.5 4.0 3.5 3.0 2.5 2.0 1.5 1.0 0.5 0.0
f1 (ppm)

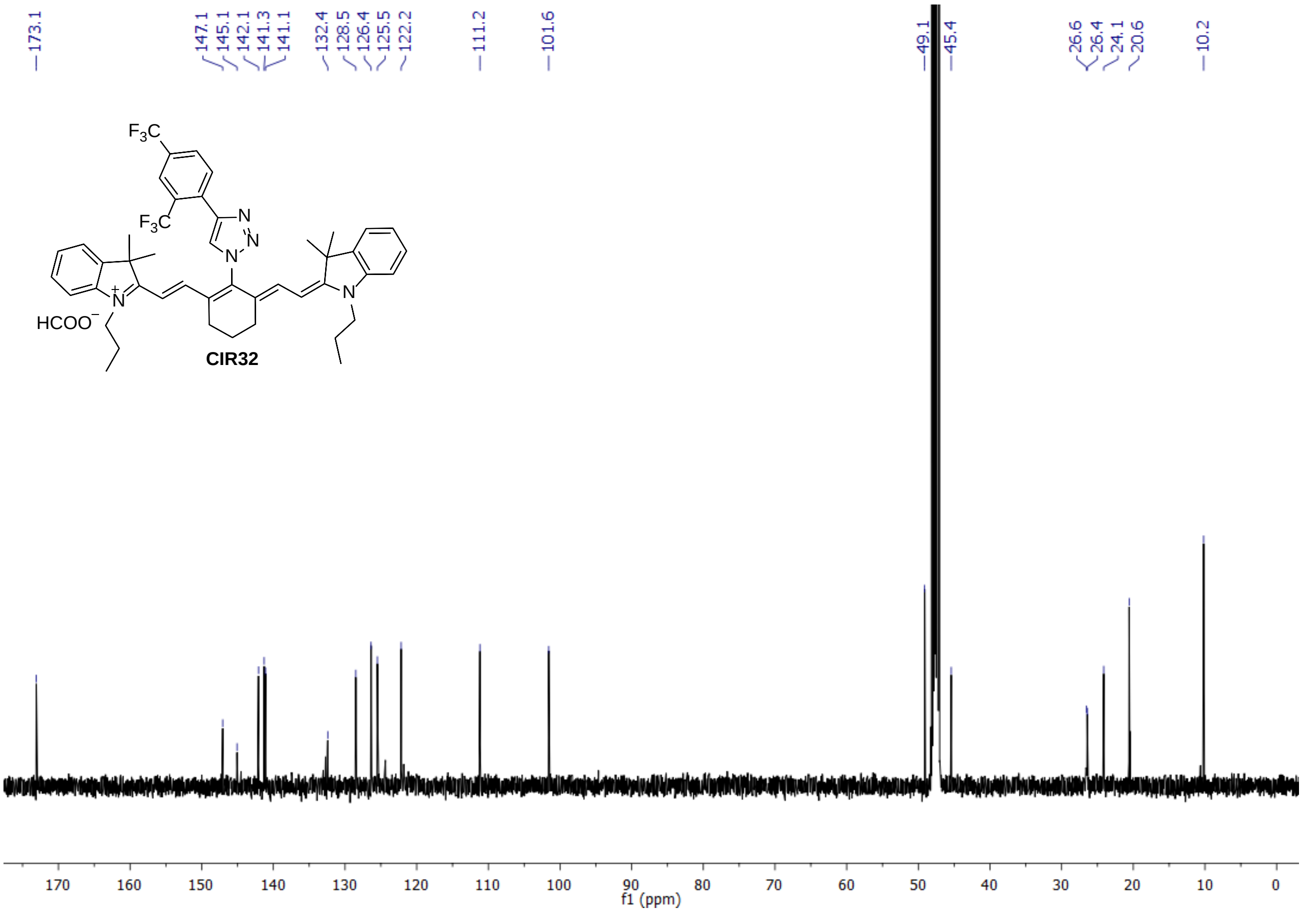
173.1
147.1 145.1 142.1 141.3 141.1
132.4 128.5 126.4 125.5 122.2
111.2
101.6
49.1 45.4
26.6 26.4 24.1 20.6
10.2
F_3C
F_3C
HCOO⁻
CIR32
170 160 150 140 130 120 110 100 90 80 70 60 50 40 30 20 10 0
f1 (ppm)

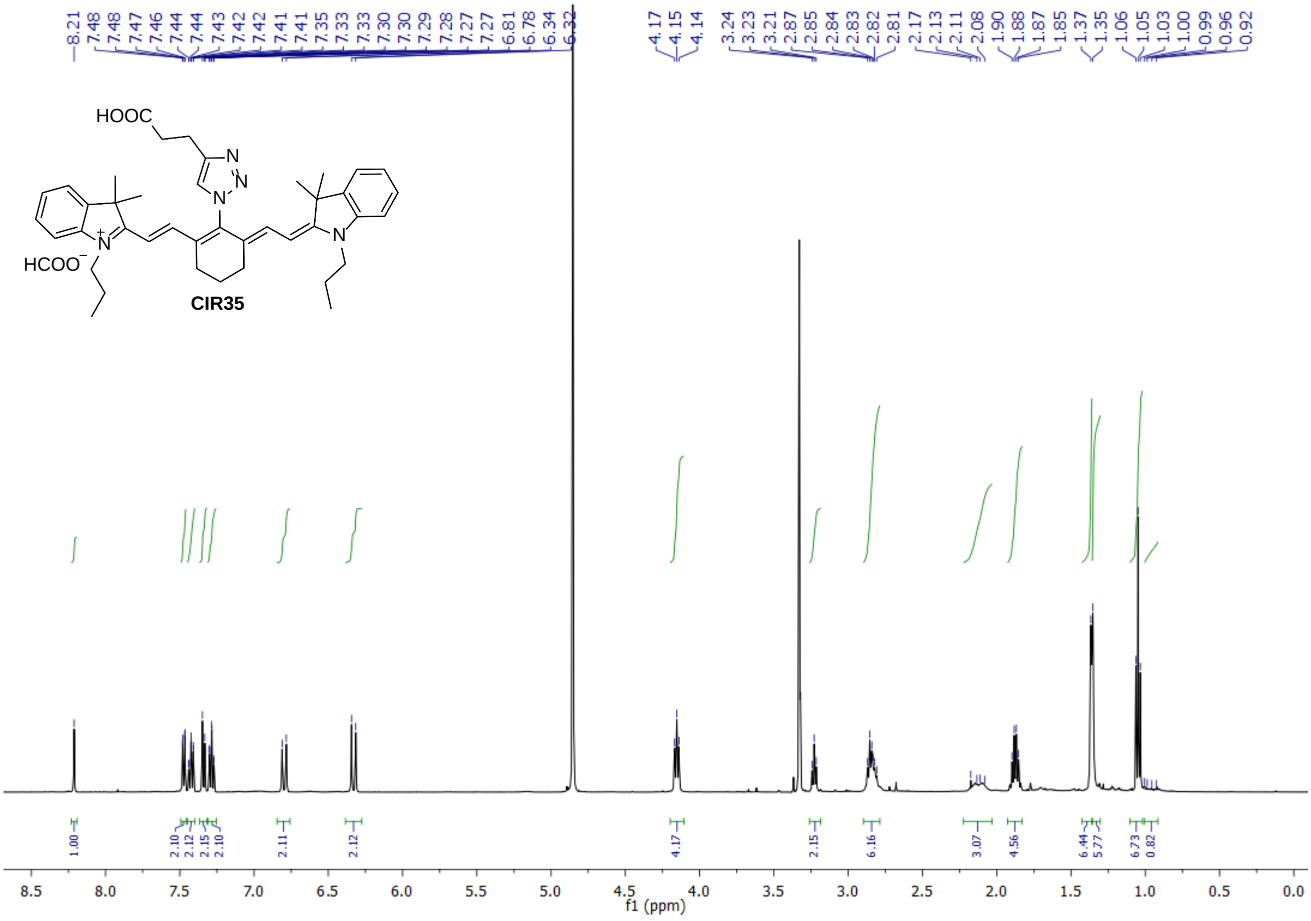
8.21
7.48
7.48
7.47
7.46
7.44
7.44
7.43
7.42
7.42
7.41
7.41
7.35
7.33
7.30
7.30
7.29
7.28
7.27
7.27
6.81
6.78
6.34
6.32
4.17
4.15
4.14
3.24
3.23
3.21
2.87
2.85
2.84
2.83
2.82
2.81
2.17
2.13
2.11
2.08
1.90
1.88
1.87
1.85
1.37
1.35
1.06
1.05
1.03
1.00
0.99
0.96
0.92
HOOC
N
N
N
N
N
HCOO⁻
CIR35
1.00
2.10
2.12
2.15
2.10
2.11
2.12
4.17
2.15
6.16
3.07
4.56
6.44
5.77
6.73
0.82
f1 (ppm)

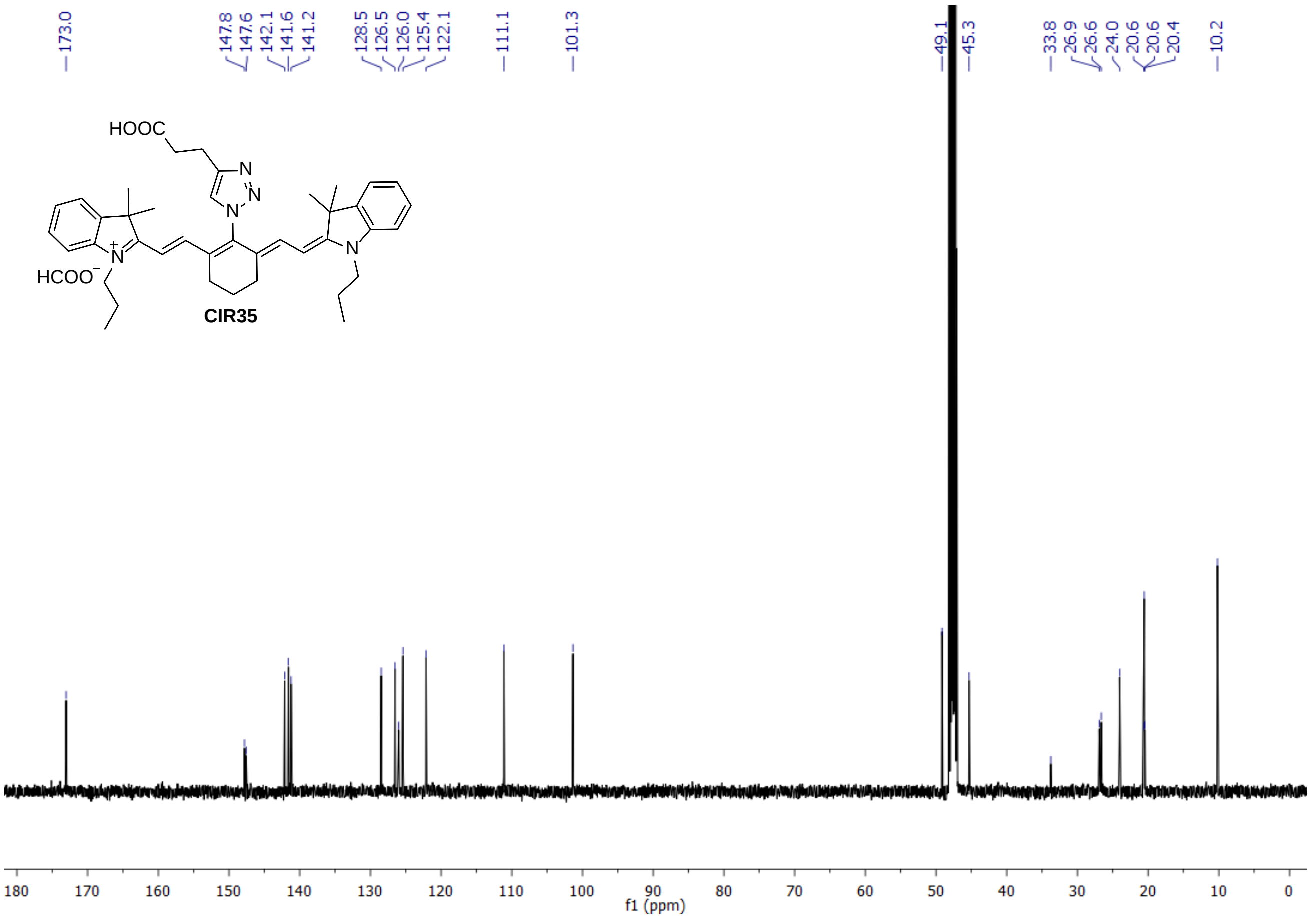
173.0
147.8
147.6
142.1
141.6
141.2
128.5
126.5
126.0
125.4
122.1
111.1
101.3
49.1
45.3
33.8
26.9
26.6
24.0
20.6
20.6
20.4
10.2
HOOC
N
N
N
N
N
HCOO⁻
CIR35
f1 (ppm)

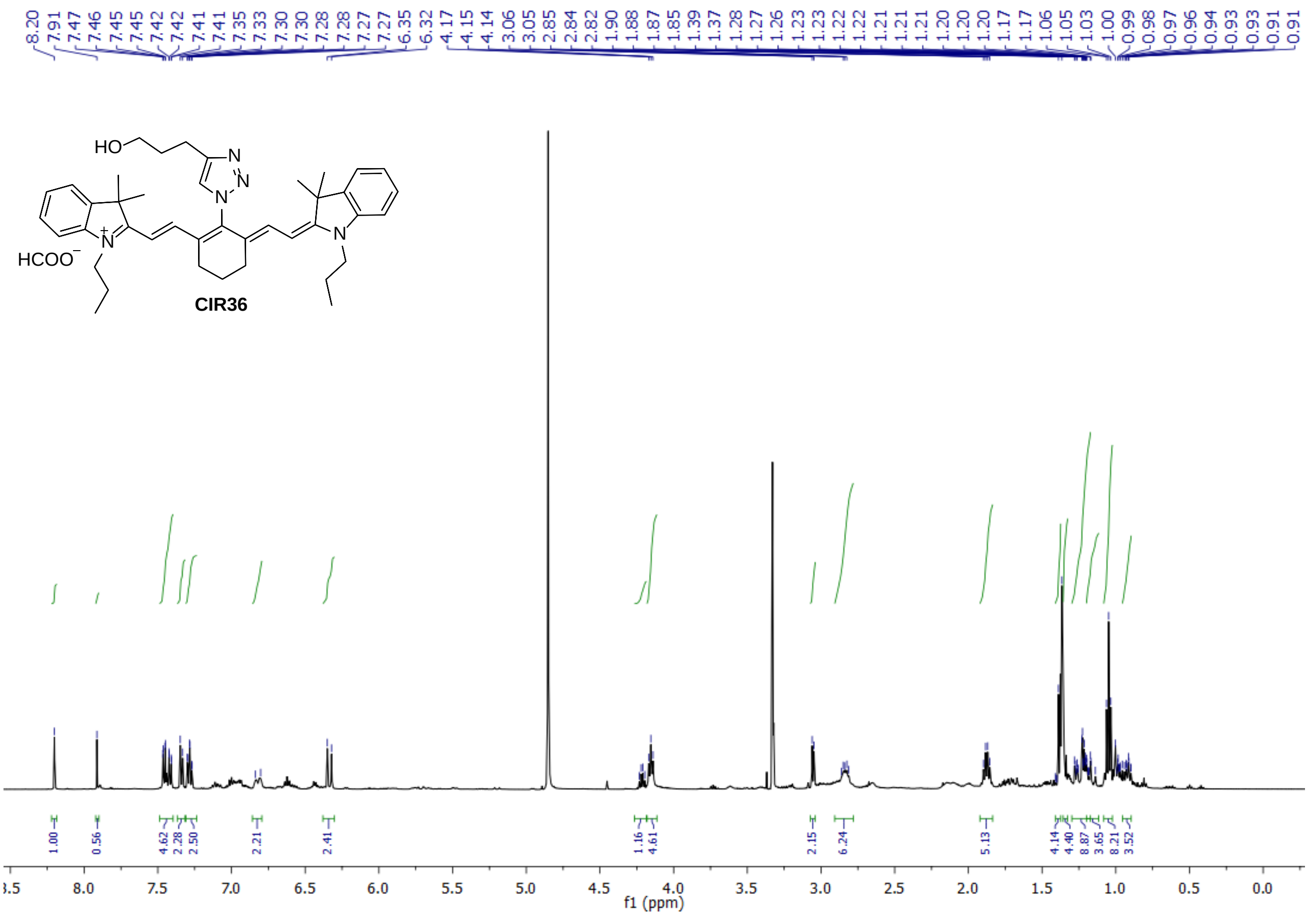

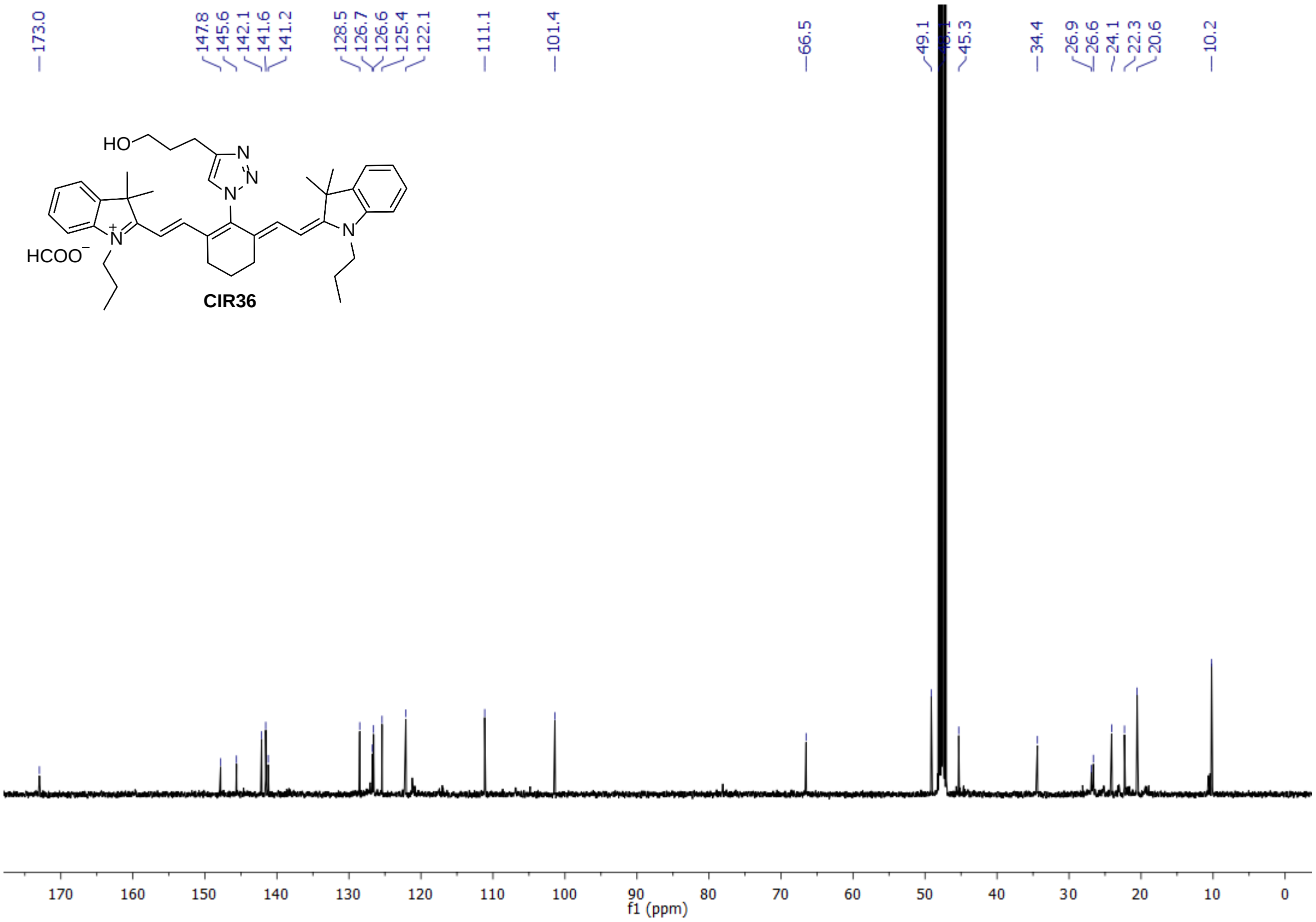

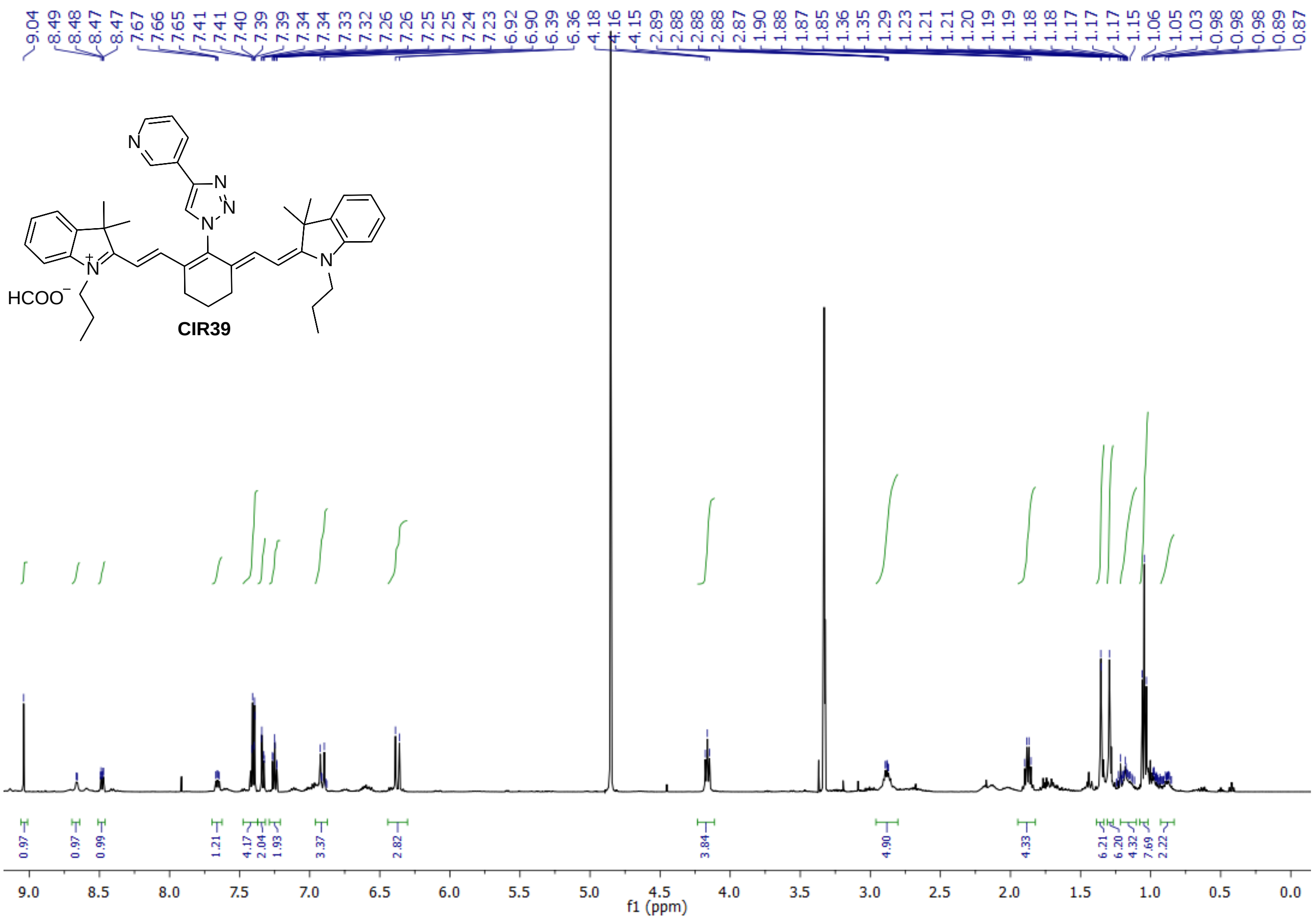
9.04 8.49 8.48 8.47 8.47 7.67 7.66 7.65 7.41 7.41 7.40 7.39 7.39 7.34 7.34 7.33 7.32 7.26 7.26 7.25 7.25 7.24 7.23 6.92 6.90 6.39 6.36 4.18 4.16 4.15 2.89 2.88 2.88 2.88 2.87 1.90 1.88 1.87 1.85 1.36 1.35 1.29 1.23 1.21 1.21 1.20 1.19 1.19 1.18 1.18 1.17 1.17 1.17 1.15 1.06 1.05 1.03 0.98 0.98 0.98 0.89 0.87
HCOO⁻
CIR39
0.97 0.97 0.99 1.21 4.17 2.04 1.93 3.37 2.82 3.84 4.90 4.33 6.21 6.20 4.32 7.69 2.22
9.0 8.5 8.0 7.5 7.0 6.5 6.0 5.5 5.0 4.5 4.0 3.5 3.0 2.5 2.0 1.5 1.0 0.5 0.0
f1 (ppm)

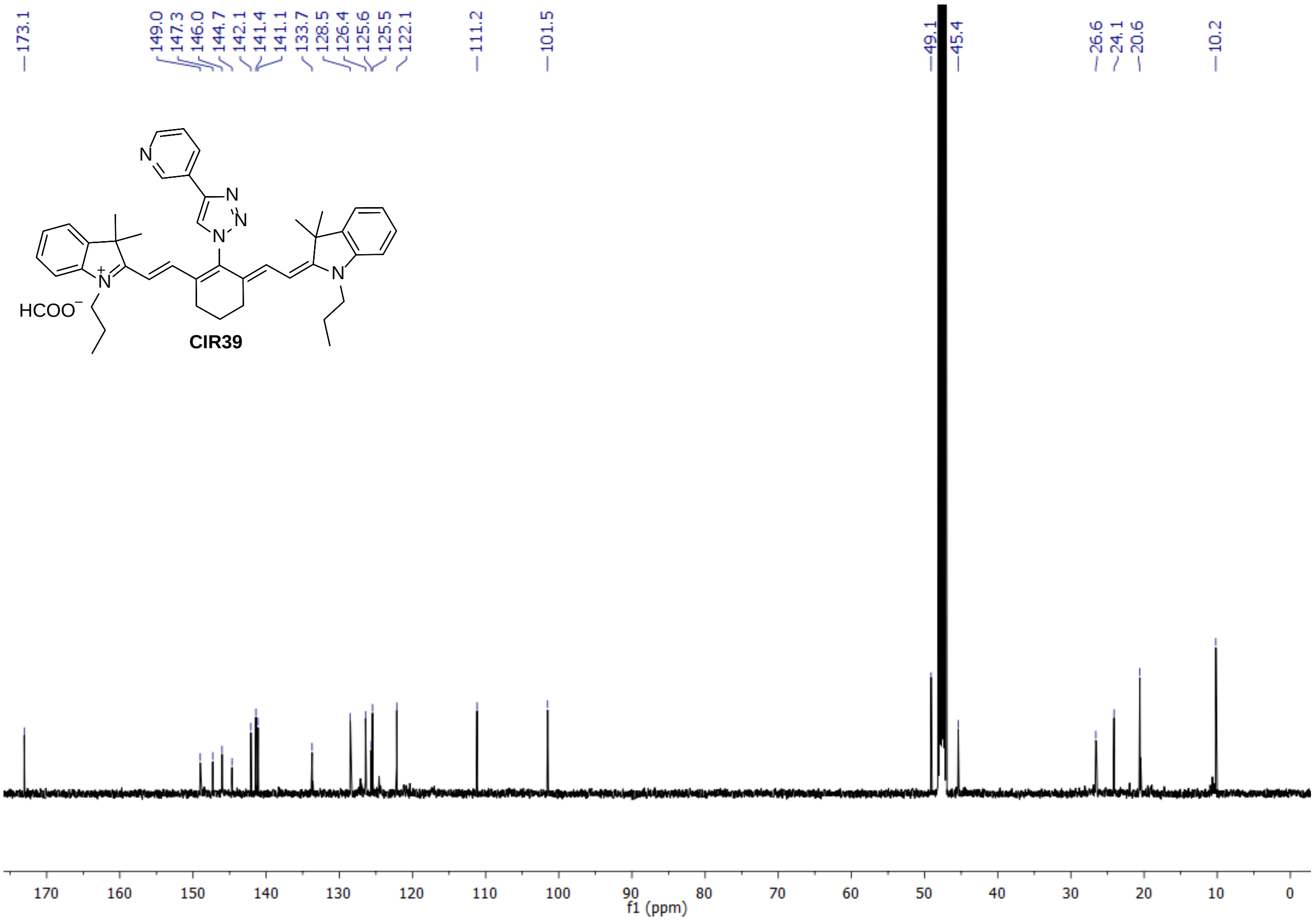
173.1 149.0 147.3 146.0 144.7 142.1 141.4 141.1 133.7 128.5 126.4 125.6 125.5 122.1 111.2 101.5 49.1 45.4 26.6 24.1 20.6 10.2
HCOO⁻
CIR39
170 160 150 140 130 120 110 100 90 80 70 60 50 40 30 20 10 0
f1 (ppm)

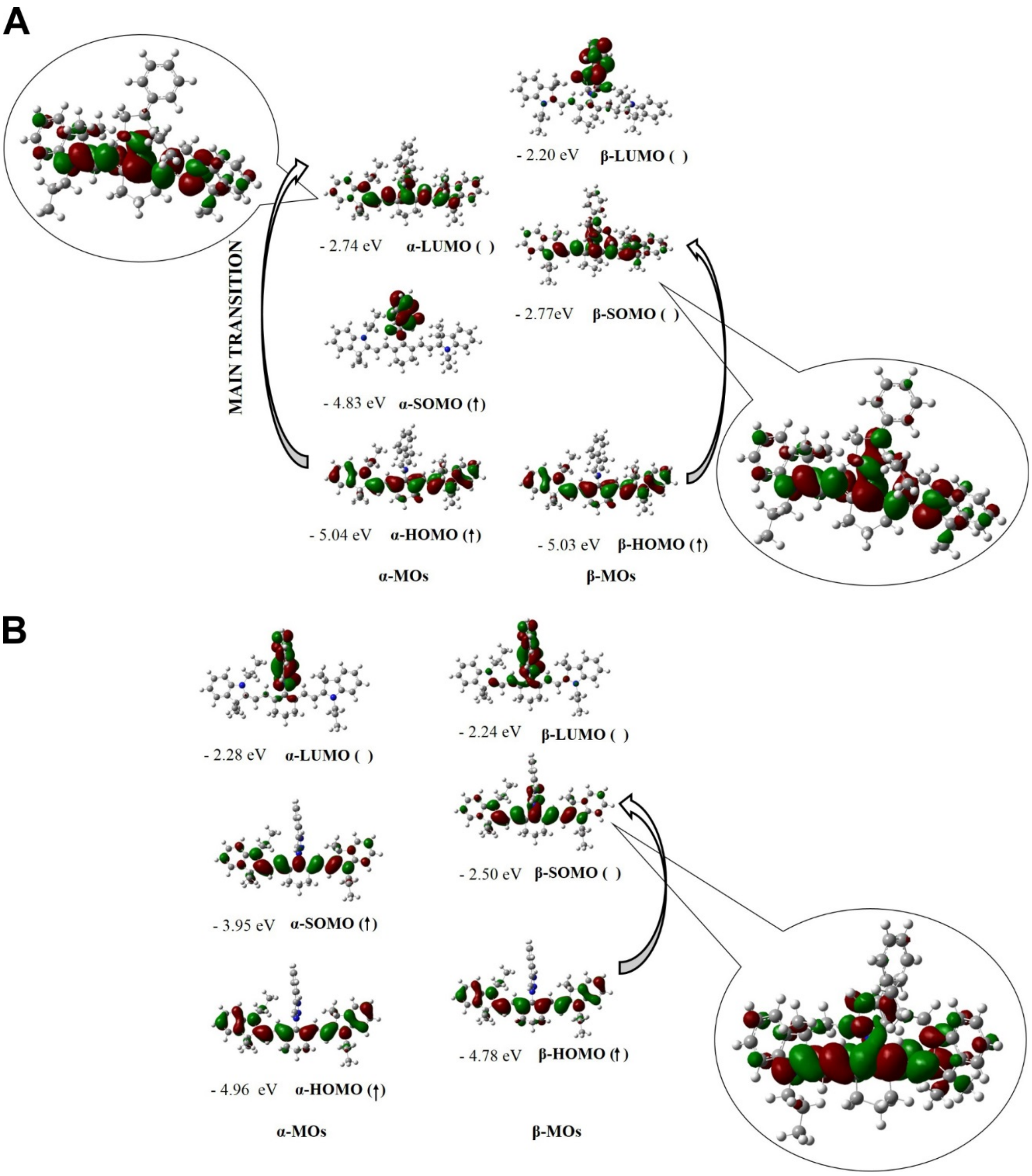

Fig. S1. Shape and energy of frontier orbitals for compound **1** (A) and compound **2** (B), calculated at the B3LYP/6-31G* level using EtOH as the solvent.

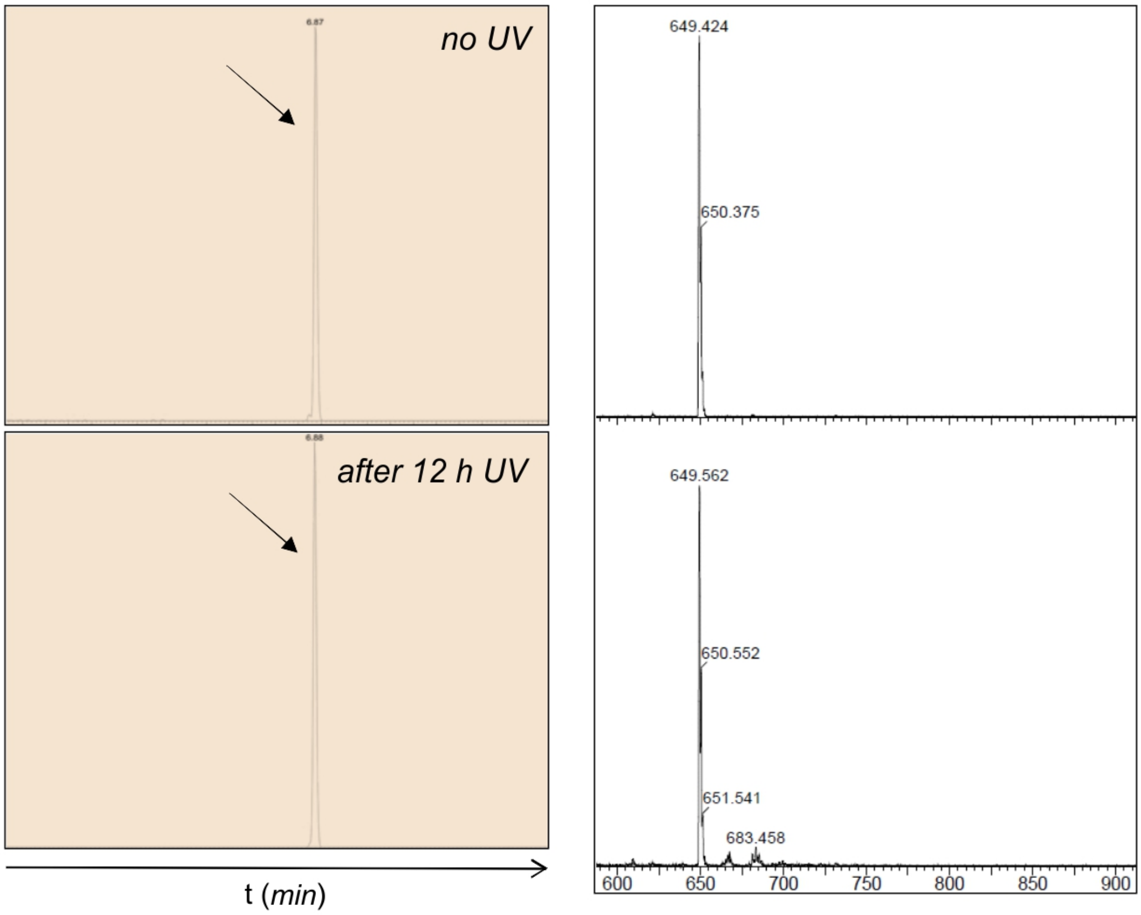

Fig. S2. HPLC-MS analysis (*left*: UV detection, *right*: MS detection) of **CIR38** before and after 12 h irradiation. Arrows pointing at intact **CIR38** ($M^{+}_{calc.}$: 649.4).

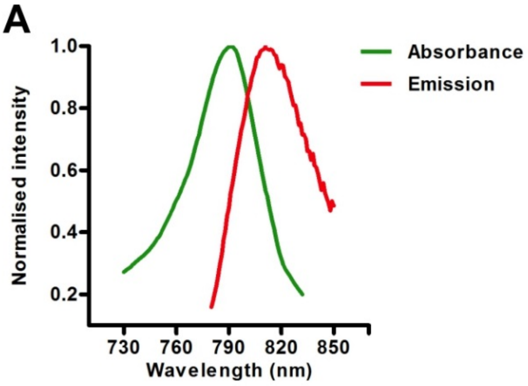

B

	$\lambda_{abs.}$ (nm)	$\lambda_{em.}$ (nm)
**CIR38**	794	818
**CIR38SE**	795	820
**CIR38M**	796	818

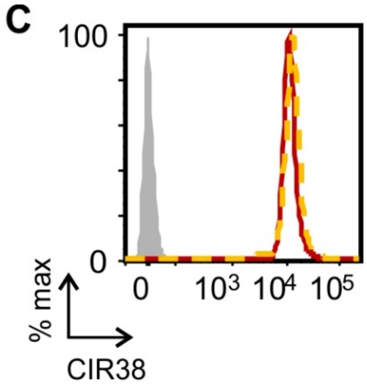

Fig. S3. A) Absorbance and fluorescence spectra of **CIR38M** in PBS. B) Summary table for the spectral characterisation data of **CIR38** derivatives in EtOH. C) Flow cytometric analysis and NIR fluorescence emission of CD4+ T cells after labelling with **CIR38SE** (dashed orange) or **CIR38M** (solid red) under the same experimental conditions (10 μM) in comparison to unlabelled cells (grey shaded area).

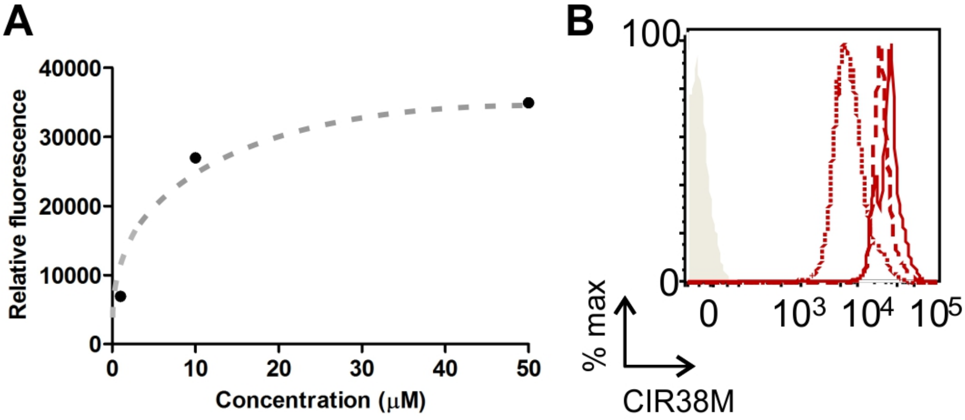

Fig. S4. A) Fluorescence saturation curve for **CIR38M** upon labelling CD4+ T cells. B) Flow cytometric analysis and NIR fluorescence emission of CD4+ T cells after labelling with **CIR38M** at different concentrations (1 μM: dotted line, 10 μM: dashed line, 50 μM: solid line) in comparison to unlabelled cells (grey shaded area).

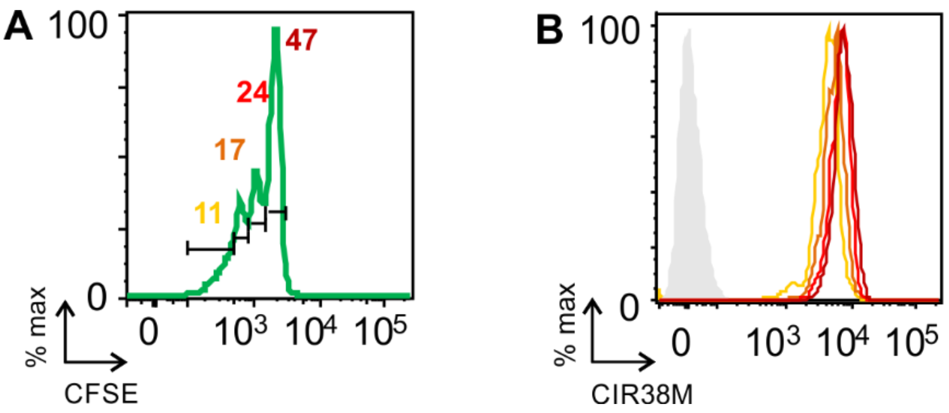

Fig. S5. A) Murine CD4+ T cells were simultaneously labelled with **CIR38M** and **CFSE** before culturing on anti-CD3 and anti-CD28 coated plates. After 3 days of *in vitro* culture, cells were analyzed by flow cytometry and categorized by their number of cell divisions as determined by **CFSE** dilution. **CFSE** fluorescence intensity was used to split the cells into four populations: undivided (accounting for 47% of the total, dark red), cells undergoing one round of proliferation (24% total, light red), two rounds of proliferation (17% total, orange) and three or more rounds of proliferation (11% total, yellow). B) **CIR38M** fluorescence emission on the four cell populations defined in A.

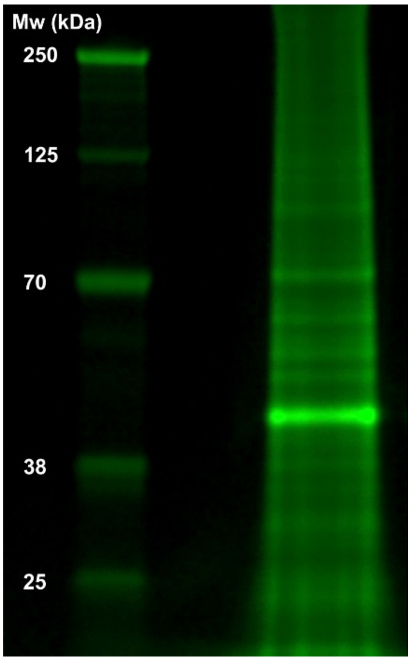

Fig. S6. SDS-PAGE analysis of lysates from CD4+ T cells upon labelling with **CIR38M** and visualisation under an Odyssey® CLx imaging system ($\lambda_{exc.}$ ~ 800 nm). Protein ladder: Chameleon 800 (LI-COR).

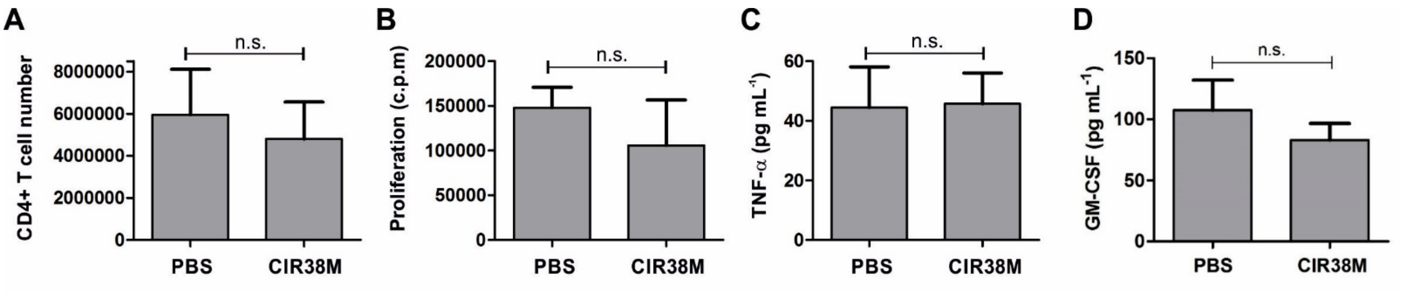

Fig. S7. A) Equal numbers of CD4+ T cells were labelled with **CIR38M** or treated with PBS, incubated in culture media overnight at 37 °C, and viable cells were counted 24 h later. Values are represented as means ± s.d. (n=3). B) CD4+ T cells were labelled with **CIR38M** or treated with PBS and subsequently cultured on anti-CD3 and anti-CD28 coated plates. After 48 h, cell proliferation was assessed by the addition of [^{3}H]-thymidine for the last 18 h of culture. Values are represented as means ± s.d. (n=3). C) CD4+ T cells were labelled with **CIR38M** or treated with PBS and subsequently cultured on anti-CD3 and anti-CD28 coated plates. TNF-α concentrations in the supernatant were determined by ELISA 72 h later. Values are represented as means ± s.d. (n=3); $p > 0.05$ for n.s. D) ELISA determination of cytokine production by **CIR38M**-labelled and unlabelled CD4+ T cells. Values are represented as means ± s.d. (n=6 per group). $p > 0.05$ for n.s.

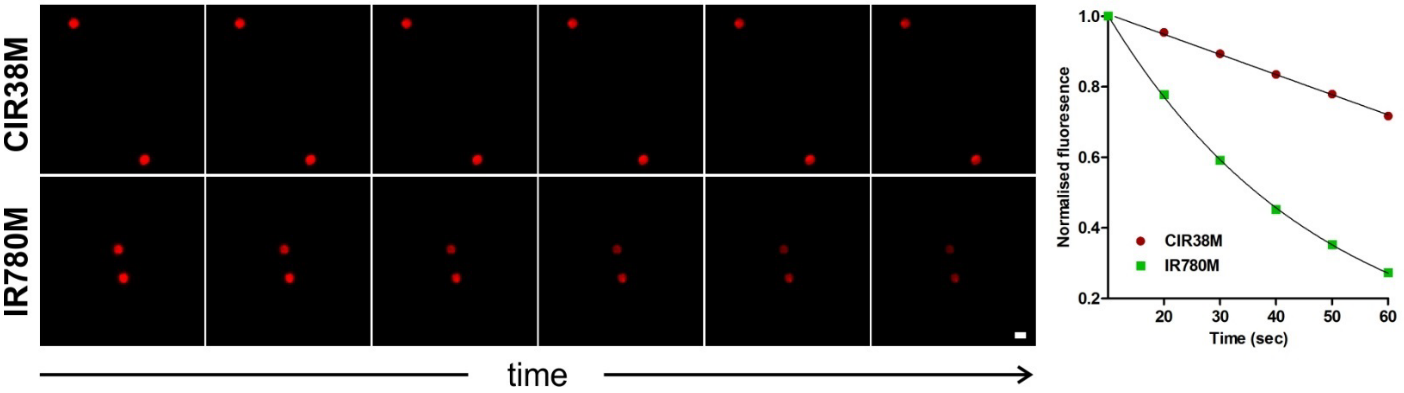

Fig S8. Time-lapse comparative NIR cell imaging of **CIR38M** and **IR780M**-labelled CD4+ T cells under fluorescence microscopy. Samples were constantly irradiated and individual images were acquired every 10 seconds for a total of 1 min. Scale bar: 5 μm. Normalised fluorescence intensity values for both sets of images were fitted to one phase exponential decay regressions (left plot).

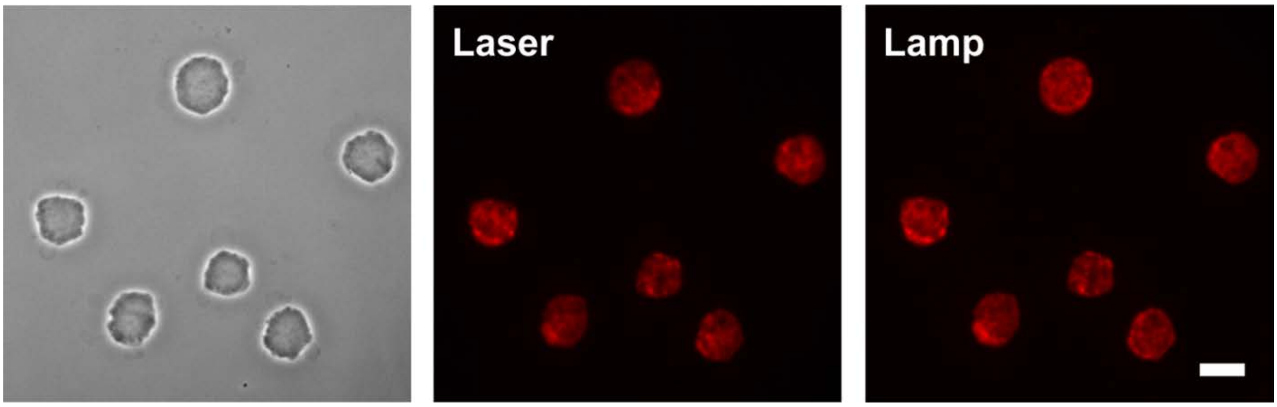

Fig. S9. Comparative fluorescence cell imaging of **CIR38M**-labelled CD4+ T cells under a fluorescence microscope equipped with Ti-sapphire laser and mercury lamp excitation sources. Scale bar: 5 μm.

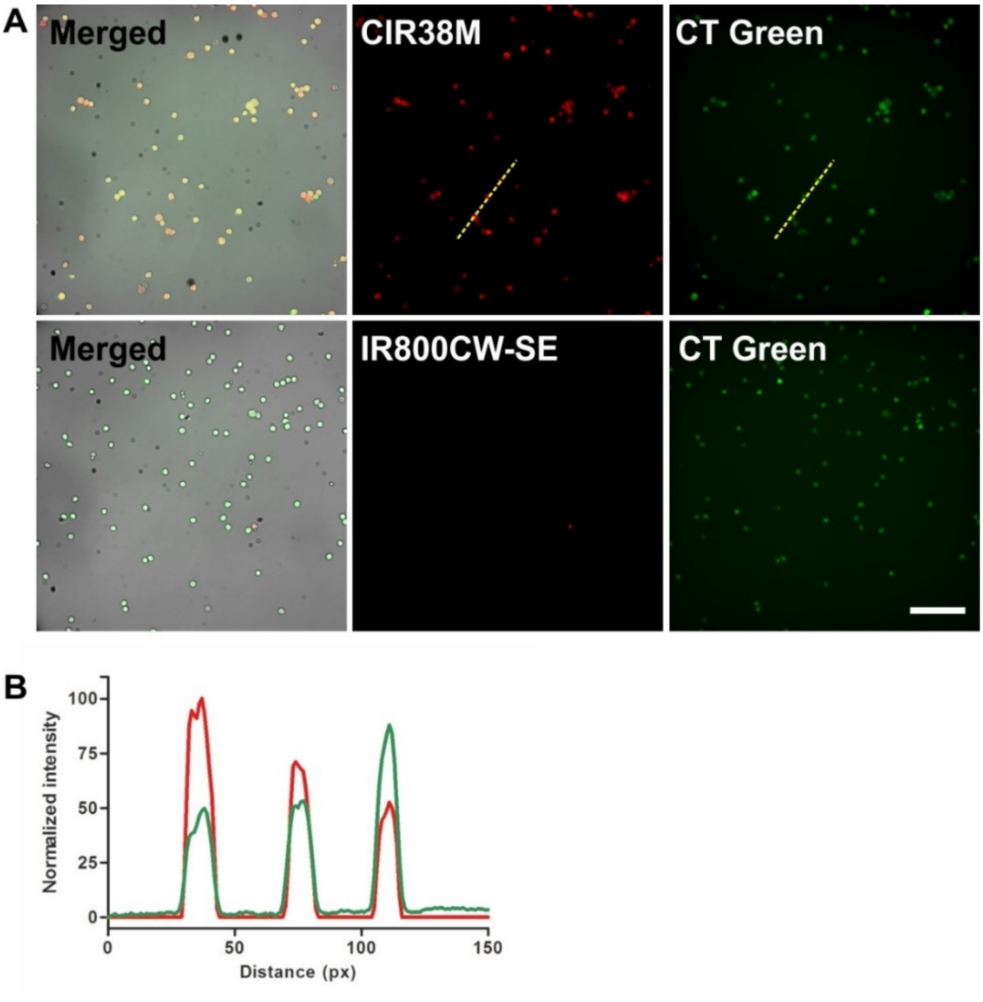

Fig. S10. A) Low-magnification fluorescence microscopy images of CD4+ T cells upon staining with CellTracker Green (green) and **CIR38M** or **IR800CW-SE** (red). Scale bar: 50 μm. B) Plot profile analysis of the dashed yellow line in the top panel in (A), which correlates the intracellular staining of **CIR38M** (red) to CellTracker Green (green).

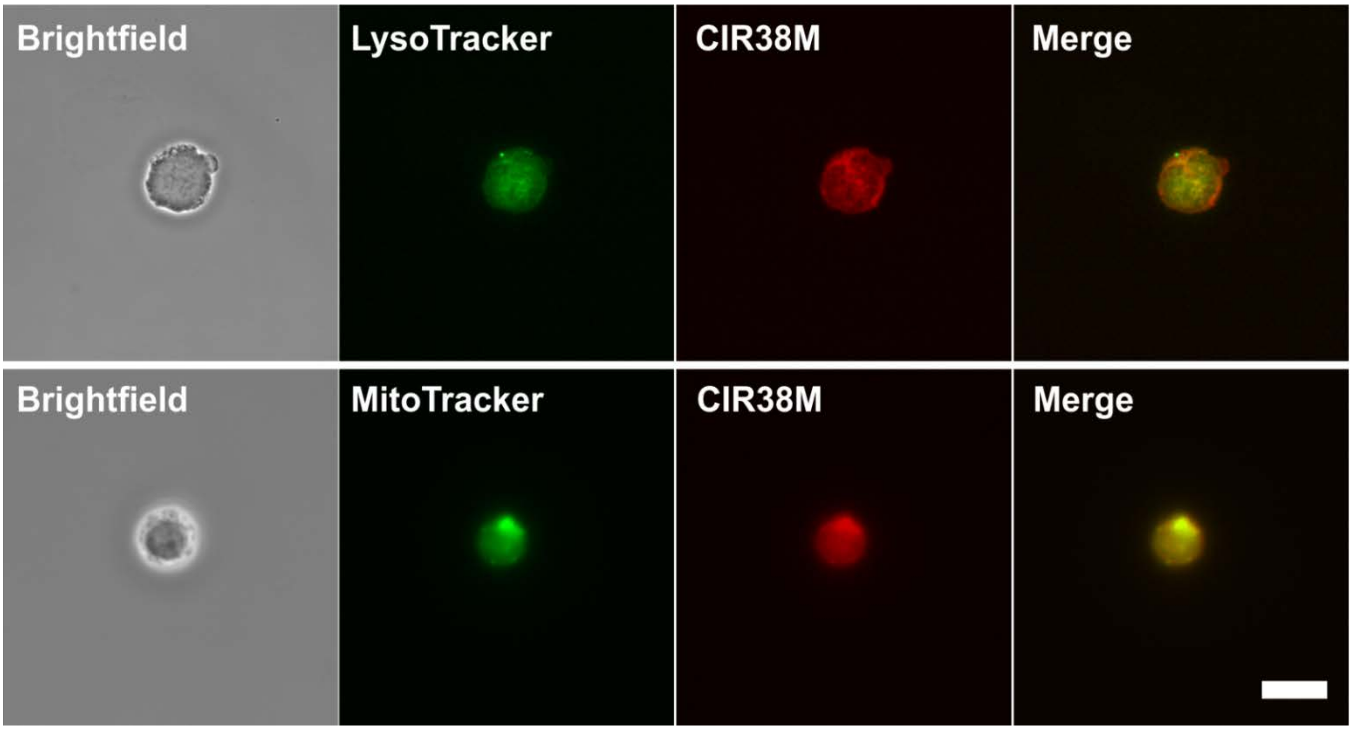

Fig. S11. High-magnification fluorescence microscopy images of CD4+ T cells upon co-staining with LysoTracker Red DND-99 (100 nM, green), MitoTracker Red CMXRos (500 nM, green) and **CIR38M** (10 μM, red). Scale bar: 5 μm.

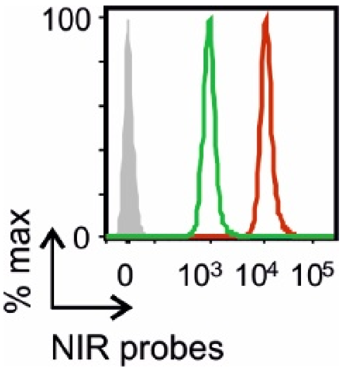

Fig. S12. Flow cytometric analysis CD4+ T cells upon labelling with 10 μM **CIR38M** (red line), 10 μM **IR800CW-SE** (green line), or left unstained (grey shaded area).

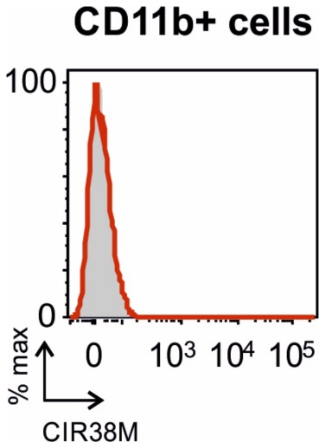

Fig. S13. Histogram depicting lack of fluorescence in host CD11b+ myeloid cells retrieved from the iLNs from mice injected with **CIR38M**-labelled CD4+ T cells (red line) and with unlabelled CD4+ T cells (grey shaded area) on day 7.

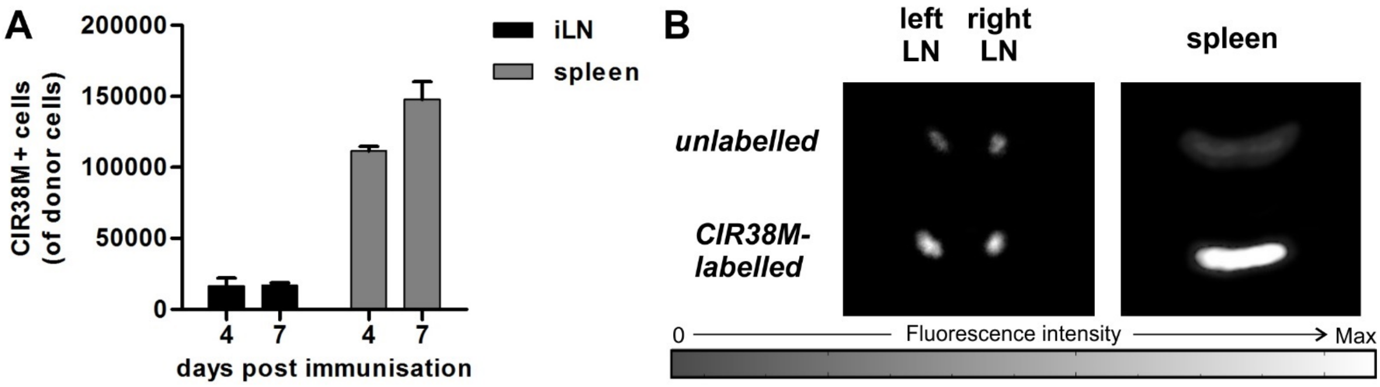

Fig. S14. Mice were injected i.v. with **CIR38M**-labelled pOVA-reactive CD45.1+ CD4+ OT-II T cells and immunised by injection of 50 μL of 10 μg mL^{-1} pOVA in CFA. Mice were sacrificed and inguinal lymph nodes (iLN) and spleens harvested on days 4 or 7. A) Total numbers of **CIR38M**-positive donor cells were determined by flow cytometry. Values are represented as means ± s.e.m (n=3 per group). B) *Ex vivo* tissue fluorescence images ($\lambda_{exc.}$ ~ 760 nm; $\lambda_{em.}$ ~ 800-900 nm) of lymph nodes and spleen tissue from mice that had been injected the same amount of **CIR38M**-labelled or unlabelled T cells for 7 days. Max: 5 × 10^7 photons s^{-1}.

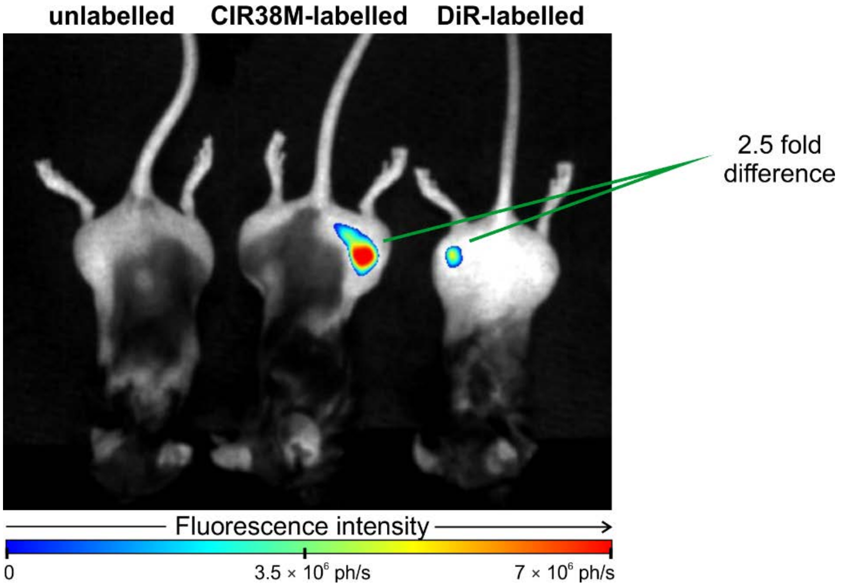

Fig. S15. Whole-body fluorescence images ($\lambda_{exc.}$ ~ 760 nm; $\lambda_{em.}$ ~ 800-900 nm) of B10.PLxC57BL/6 mice (representative image from 3 mice pairs) 2 days after the tail vein i.v. injection of **DiR**-labelled (right), **CIR38M**-labelled (center) or unlabelled CD4+ Tg4 T cells (left). Cell transfer was followed by subcutaneous immunisation with 10 µg of MBP Ac1-9(4Tyr) peptide emulsified in CFA containing 50 µg of heat-killed *Mycobacterium tuberculosis* H37Ra at a total final volume of 100 µL injected subcutaneously into the hind legs.

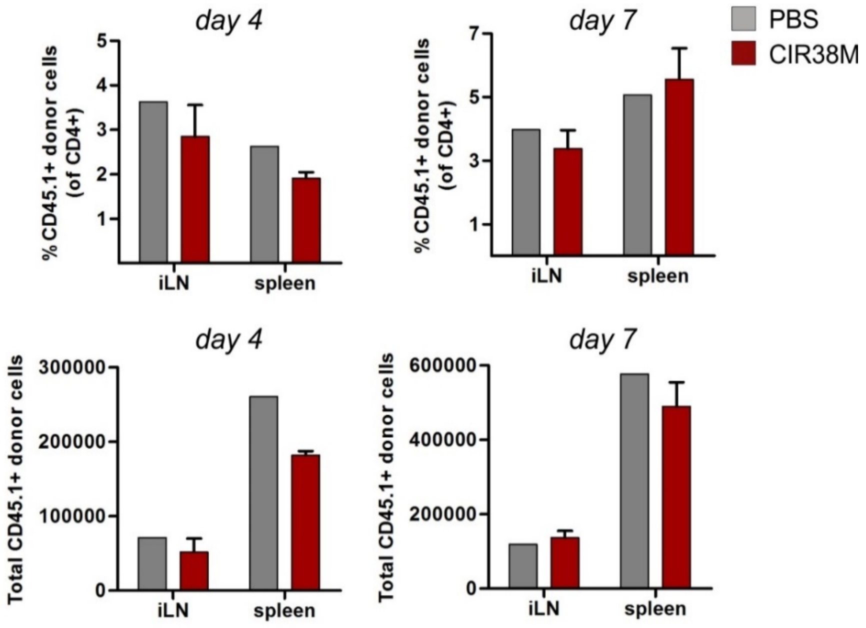

Fig. S16. Proportion and total numbers of CD45.1+ donor cells retrieved from iLNs and spleen on days 4 or 7. Values are represented as means ± s.e.m (n=3 for the **CIR38M**-labelled group).

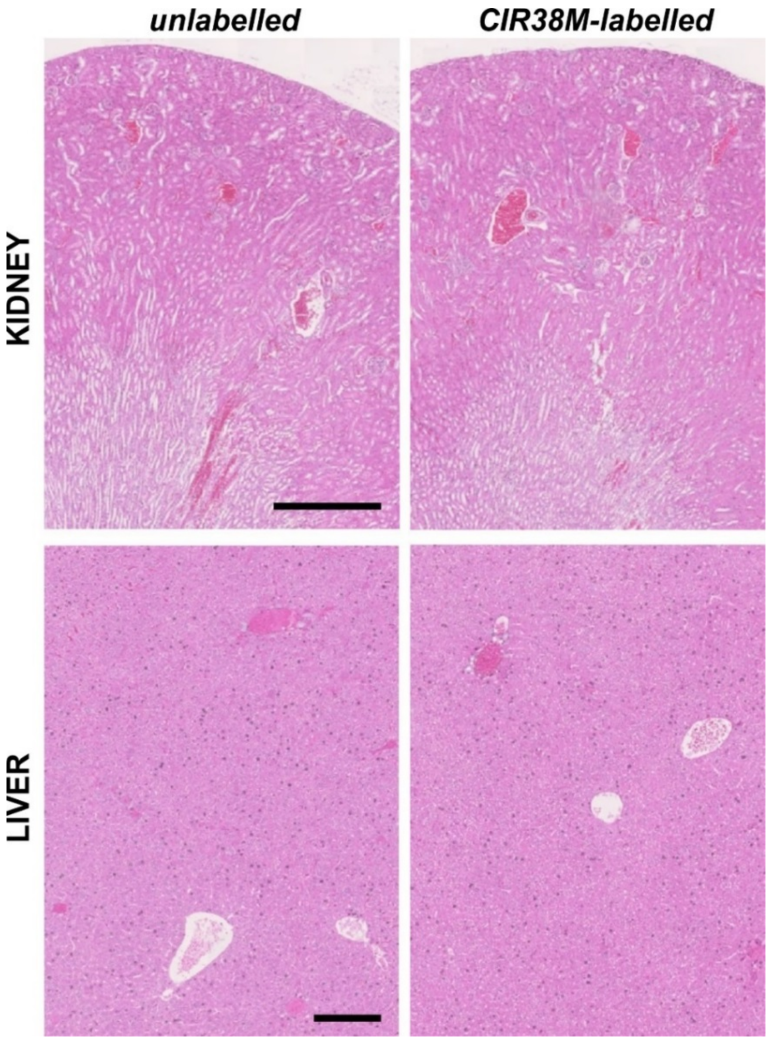

Fig. S17. Histological analysis of kidney (*top panels*) and liver (*bottom panels*) tissues from mice that had been injected with unlabelled or **CIR38M**-labelled CD4+ T cells and immunised with pOVA and CFA. Tissues were harvested 2 days after cell injection, fixed in 4% neutral buffered formalin, paraffin-embedded and stained with haematoxylin and eosin (H&E). Scale bars: 500 μm (top), 200 μm (bottom).

Table S1. Electronic transitions computed for compounds **1** and **2** at the TD-B3LYP/6-31G*//B3LYP/6-31G*, TD-M06-2X/6-31G*//M06–2X/6-31G*, TD-PBE0/6-31G*//PBE0/6-31G* and TD-wB97xd/6-31G*/ wB97xd/6-31G* levels of theory, in gas phase and in EtOH solution.

		**COMPOUND 1 (*N*-amine)**				**COMPOUND 2 (*N*-triazole)**			
	**Solvent**	**E (eV)**	**λ (nm)**	***f***	**Main component of the transition (% contribution)**	**E (eV)**	**λ (nm)**	***f***	**Main component of the transition (% contribution)**
**TD-B3LYP/6-31G***	**EtOH**	2.08	596	1.9236	α-HOMO → α-LUMO (49.62%)<br>β-HOMO → β-SOMO (49.45%)	1.59	782	0.2284	α-SOMO → α-LUMO+2 (19.08%)<br>β-HOMO → β-SOMO (77.29%)<br>β-HOMO → β-LUMO (1.61%)
						2.35	528	2.0565	α-SOMO → α-LUMO+2 (71.74%)<br>β-HOMO → β-SOMO (16.31%)<br>β-HOMO → β-LUMO (6.19%)
	**None**	2.16	573	1.8163	α-HOMO → α-LUMO (50.43%)<br>β-HOMO → β-SOMO (49.65%)<br>α-HOMO ← α-LUMO (1.32%)<br>β-HOMO ← β-SOMO (1.42%)	1.89	656	0.3313	α-HOMO → α-LUMO (68.34%)<br>β-HOMO → β-SOMO (19.24%)<br>β-HOMO → β-LUMO (8.14%)
						2.46	504	1.1710	α-SOMO → α-LUMO+2 (46.61%)<br>β-HOMO → β-SOMO (6.94%)<br>β-HOMO → β-LUMO (31.61%)<br>β-HOMO → β-LUMO+1 (6.64%)
**TD-M062X/6-31G***	**EtOH**	2.28	544	1.9461	α-HOMO → α-LUMO (27.42%)<br>α-SOMO → α-LUMO (21.33%)<br>β-HOMO → β-LUMO +1 (46.41%)	1.63	762	0.3756	α-SOMO → α-LUMO+2 (12.04%)<br>β-HOMO → β-SOMO (82.68%)
						2.58	480	1.9499	α-SOMO → α-LUMO+2 (77.94%)<br>β-HOMO → β-SOMO (12.04%)
	**None**	2.29	542	1.8462	α-HOMO-1 → α-LUMO+1 (1.30%)<br>α-HOMO → α-LUMO (49.94%)<br>β-HOMO-1 → β-LUMO+1 (1.1%)<br>β-HOMO → β-SOMO (46.22%)	1.62	767	0.3108	α-SOMO → α-LUMO+3 (13.53%)<br>β-HOMO → β-SOMO (79.63%)
						2.63	465	1.7330	α-SOMO → α-LUMO+3 (69.97%)<br>β-HOMO → β-SOMO (12.56%)
**TD-PBE0/6-31G***	**EtOH**	2.16	574	2.0300	α-HOMO → α-LUMO (47.90%)<br>α-SOMO → α-LUMO (2.90%)<br>β-HOMO → β-SOMO (49.01%)	1.71	726	0.2203	α-SOMO → α-LUMO+2 (20.41%)<br>β-HOMO → β-SOMO (76.39%)
						2.42	512	2.0344	α-SOMO → α-LUMO+2 (68.97%)<br>β-HOMO → β-SOMO (20.33%)
	**None**	2.25	551	1.8667	α-HOMO → α-LUMO (50.20%)<br>β-HOMO → β-SOMO (49.51%)<br>β-HOMO ← β-SOMO (1.06%)	1.53	811	0.1454	α-SOMO → α-LUMO (11.09%)<br>α-SOMO → α-LUMO+3 (10.37%)<br>β-HOMO → β-LUMO (71.50%)

						2.44	508	0.9514	α-SOMO → α-LUMO+3 (36.78%)<br>β-HOMO → β-LUMO (25.91%)<br>β-HOMO → β-LUMO+1 (19.58%)
**TD-WB97XD/6-31G***	**EtOH**	2.27	546	2.0903	α-SOMO → α-LUMO (46.03%)<br>β-HOMO → β-SOMO (45.74%)	1.90	651	0.3232	α-SOMO → α-LUMO+2 (12.53%)<br>β-HOMO → β-SOMO (76.02%)
						2.69	460	1.9252	α-SOMO → α-LUMO+2 (69.77%)<br>β-HOMO → β-SOMO (13.68%)
	**None**	2.32	533	1.9332	α-HOMO-1 → α-LUMO+1 (1.87%)<br>α-HOMO → α-LUMO (2.98%)<br>α-SOMO → α-LUMO (44.98%)<br>β-HOMO-1 → β-LUMO+1 (1.73%)<br>β-HOMO → β-SOMO (46.13%)	1.87	662	0.2899	α-SOMO → α-LUMO+3 (13.03%)<br>β-HOMO → β-SOMO (38.69%)<br>β-HOMO → β-LUMO (37.87%)
						2.71	452	1.6290	α-SOMO → α-LUMO+3 (55.77%)<br>β-HOMO → β-LUMO (12.24%)

Table S2. Chemical and spectral characterisation data for the CIR fluorophores.

Compound code	Chemical structure	$M_{calc.}$ $[M+H^+]_{exp.}$	tR‡ (purity)	Yield (%)	$\lambda_{exc.}$ (nm)	$\lambda_{em.}$ (nm)	QY*
**2**	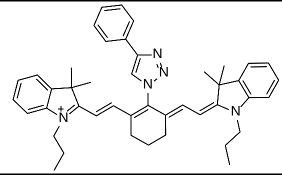	648.4 (648.5)	7.75 (100%)	48	803	840	10
**CIR1**	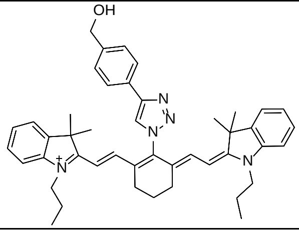	678.4 (678.3)	7.06 (100%)	55	802	839	10
**CIR2**	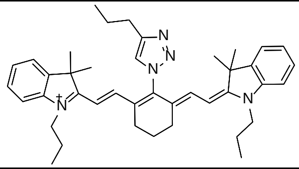	614.4 (614.6)	7.88 (98%)	37	801	831	14
**CIR3**	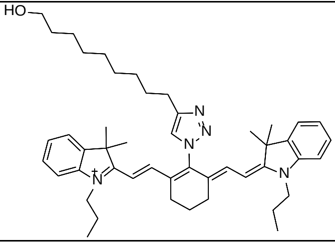	714.5 (714.5)	7.90 (97%)	17	801	835	18
**CIR4**	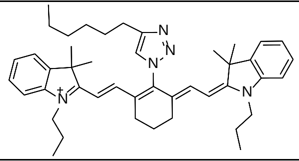	656.5 (656.5)	8.16 (98%)	18	800	839	11
**CIR6**	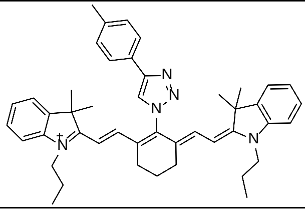	662.4 (662.4)	7.98 (100%)	34	802	833	13
**CIR7**	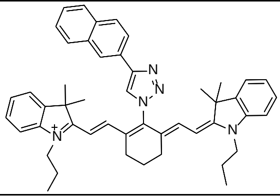	698.4 (698.4)	8.08 (100%)	19	802	840	14
**CIR8**	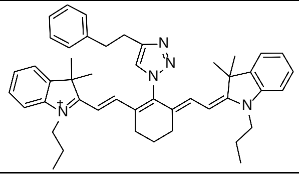	676.4 (676.4)	7.77 (100%)	16	801	839	15
**CIR9**	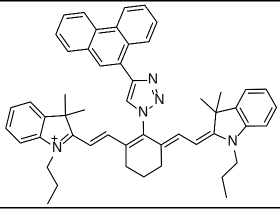	748.4 (748.3)	8.61 (100%)	18	803	835	12

**CIR10**	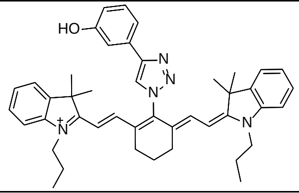	664.4<br>(664.5)	7.17<br>(100%)	13	802	834	13
**CIR11**	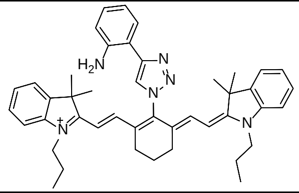	663.4<br>(663.5)	7.59<br>(100%)	11	803	840	6
**CIR12**	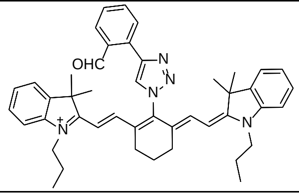	676.4<br>(676.5)	7.59<br>(100%)	18	804	840	9
**CIR13**	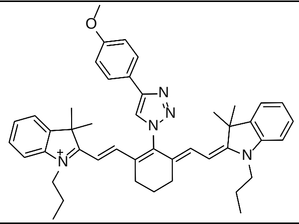	678.4<br>(678.4)	7.57<br>(100%)	14	802	838	11
**CIR14**	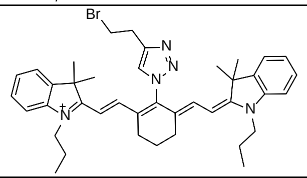	679.8<br>(680.4)	7.55<br>(42%)	19	802	839	9
**CIR15**	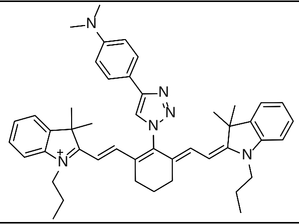	691.4<br>(691.5)	7.68<br>(100%)	14	802	839	6
**CIR16**	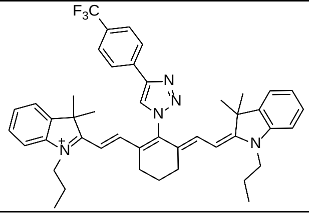	716.4<br>(716.3)	7.98<br>(100%)	12	803	838	10
**CIR17**	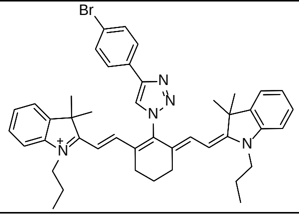	726.3<br>(726.3)	7.98<br>(100%)	20	802	840	13
**CIR18**	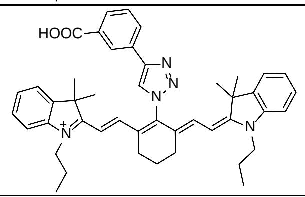	692.4<br>(692.4)	7.13<br>(100%)	12	803	836	13

**CIR19**	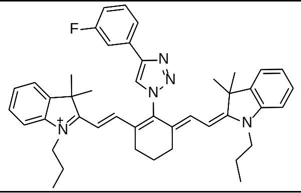	666.4 (666.5)	7.76 (100%)	48	802	832	12
**CIR20**	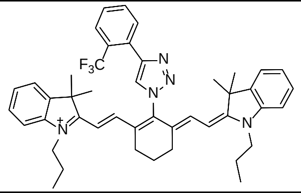	716.4 (716.5)	7.89 (85%)	27	807	840	10
**CIR21**	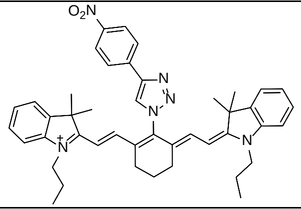	693.4 (693.4)	7.61 (100%)	19	803	839	9
**CIR22**	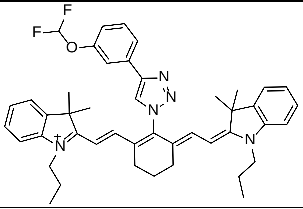	714.4 (714.4)	7.63 (100%)	42	803	839	10
**CIR23**	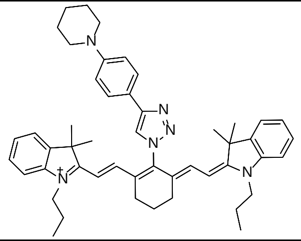	731.4 (731.5)	7.61 (100%)	13	802	836	1
**CIR24**	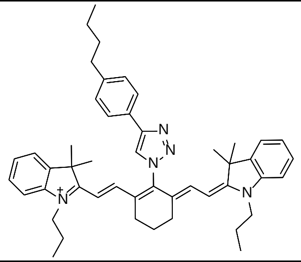	704.4 (704.4)	8.48 (100%)	16	802	840	9
**CIR25**	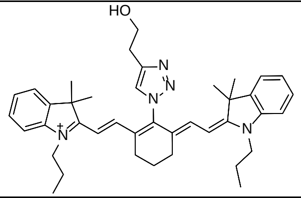	616.4 (616.4)	6.81 (100%)	30	800	829	14
**CIR26**	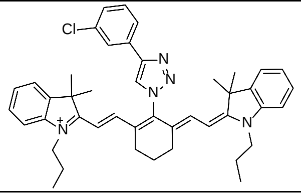	682.4 (682.4)	7.77 (100%)	16	803	840	9
**CIR27**	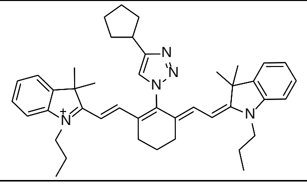	640.4 (640.5)	7.77 (100%)	14	800	840	14

**CIR28**	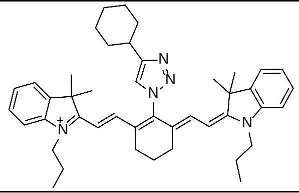	654.4 (654.4)	8.10 (89%)	33	800	833	14
**CIR29**	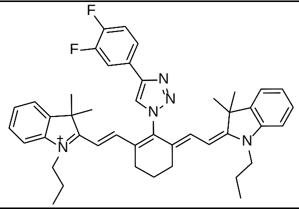	684.4 (684.4)	7.93 (100%)	25	803	838	13
**CIR30**	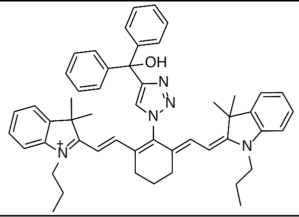	754.4 (754.5)	7.93 (100%)	33	803	835	13
**CIR31**	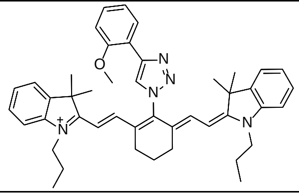	678.4 (678.5)	7.80 (95%)	31	803	838	11
**CIR32**	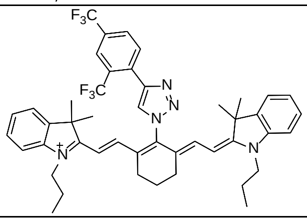	784.4 (784.3)	8.33 (100%)	21	804	840	11
**CIR33**	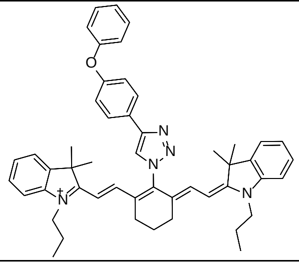	740.4 (740.3)	8.11 (100%)	28	803	840	13
**CIR34**	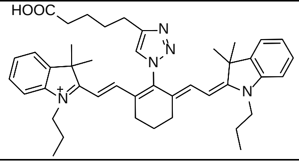	672.4 (672.5)	6.95 (100%)	13	801	833	15
**CIR35**	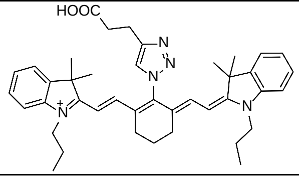	644.4 (644.3)	6.80 (100%)	26	801	838	15
**CIR36**	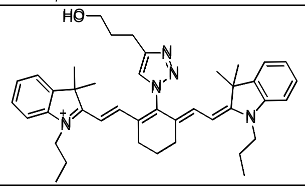	630.4 (630.3)	6.95 (100%)	52	801	831	19
**CIR37**	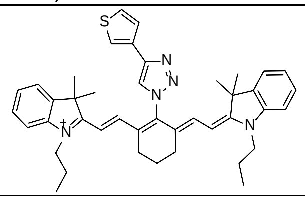	654.4 (654.3)	7.54 (94%)	38	803	832	14

**CIR38**	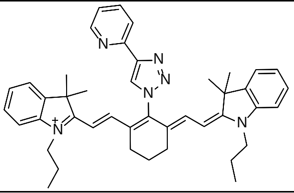	649.4 (649.5)	7.41 (100%)	22	804	840	15
**CIR39**	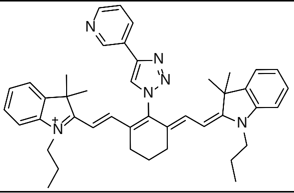	649.4 (649.4)	7.13 (100%)	32	803	835	13
**CIR40**	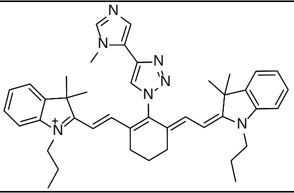	652.4 (652.5)	6.37 (69%)	14	803	835	13
**CIR41**	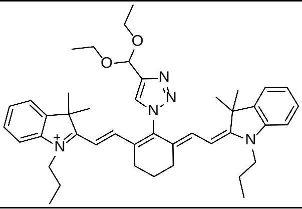	674.4 (674.5)	7.55 (100%)	36	803	838	13
**CIR42**	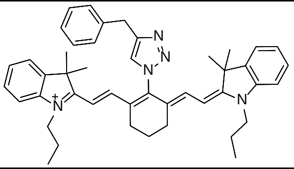	662.4 (662.4)	7.59 (100%)	33	802	838	13
**CIR43**	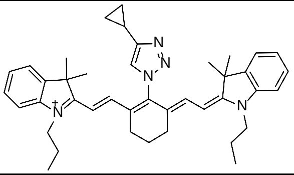	612.4 (612.4)	7.31 (100%)	35	801	835	15
**CIR44**	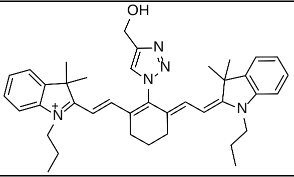	602.4 (602.4)	6.81 (100%)	55	801	835	14
**CIR45**	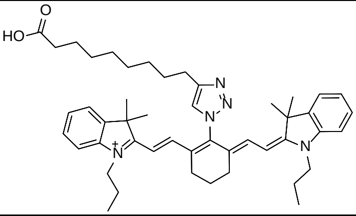	728.4 (728.6)	7.41 (100%)	3	801	832	18
**CIR46**	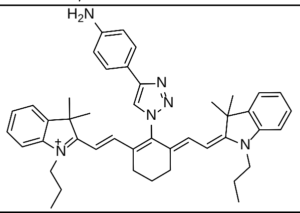	663.4 (663.5)	7.09 (100%)	2	802	835	4

‡ Retention time and UV-based purity were determined under the HPLC conditions described in the Experimental Section. * Wavelengths and fluorescence quantum yields determined in DMSO using ICG (in DMSO, QY: 12%) as the reference standard.

Table S3. HRMS characterisation of the CIR library.

Compound code	$M_{calc.}$	$M_{exp.}$
**2**	648.4061	648.4069
**CIR1**	678.4166	678.4196
**CIR2**	614.4217	614.4237
**CIR3**	714.5106	714.5107
**CIR4**	656.4687	656.4692
**CIR6**	662.4217	662.4254
**CIR7**	698.4217	698.4218
**CIR8**	676.4373	676.4379
**CIR9**	748.4373	748.4368
**CIR10**	664.4010	664.4015
**CIR11**	663.4169	663.4168
**CIR12**	676.4009	676.4002
**CIR13**	678.4166	678.4223
**CIR14**	678.3166	678.3184
**CIR15**	691.4482	691.4487
**CIR16**	716.3935	716.3939
**CIR17**	726.3166	n.d.
**CIR18**	692.3959	692.3974
**CIR19**	666.3966	666.3953
**CIR20**	716.3935	716.3938
**CIR21**	693.3912	693.3913
**CIR22**	714.3978	714.3976
**CIR23**	731.4796	731.4828
**CIR24**	704.4687	704.4664
**CIR25**	616.4010	616.4033
**CIR26**	682.3671	682.3687
**CIR27**	640.4374	640.4374
**CIR28**	654.4530	654.4582
**CIR29**	684.3872	684.3868
**CIR30**	754.4479	754.4449
**CIR31**	678.4166	678.4175
**CIR32**	784.3808	784.3834
**CIR33**	740.4323	740.4322
**CIR34**	672.4272	672.4275
**CIR35**	644.3959	644.3962
**CIR36**	630.4166	630.4184
**CIR37**	654.3625	654.3637
**CIR38**	649.4013	649.4029
**CIR39**	649.4013	649.4020
**CIR40**	652.4122	652.4152
**CIR41**	674.4428	674.4427
**CIR42**	662.4217	662.4246
**CIR43**	612.4061	612.4122
**CIR44**	602.3853	602.3843
**CIR45**	728.3898	728.3347
**CIR46**	663.4170	663.4168

Table S4. Spectral properties of heptamethine and cyanine dyes.

Compound	$\lambda_{exc.}$ (nm)	$\lambda_{em.}$ (nm)	QY (%)	ε (M^{-1} cm^{-1})	Photostability (%)
**CIR38(M)**	796	818	19±4	238,000	91±9
**IR780**	785	806	9±2	345,000	64±2
**IR800CW-SE**	785	806	16±1	295,000	93±2
**IR783**	786	810	15±2	224,000	95±2

Fluorescence quantum yields determined in DMSO using ICG (in DMSO, QY: 12%) as the reference standard. Wavelengths and extinction coefficients determined in EtOH. Photostability indicated as means ± s.e.m of chemical integrity after 12 h irradiation (50 μM PBS) (n=5).

Table S5. Biochemical analysis of serum from mice injected with unlabelled or **CIR38M**-labelled CD4+ T cells. All values are represented as means ± s.e.m and no significant statistical differences were observed between both groups ($p > 0.05$).

**Biomarker**	**PBS**	**CIR38M**
Cholesterol (mmol L^{-1})	2.22±0.21	2.51±0.09
Amylase (U L^{-1})	1580±99	2410±538
Alanine transaminase (U L^{-1})	56±24	54±19
Aspartate transaminase (U L^{-1})	135±42	167±30
Alkaline phosphatase (U L^{-1})	81±12	61±3
Albumin (g L^{-1})	22.80±0.75	22.50±0.63
Urea (mmol L^{-1})	7.90±0.21	8.97±0.32
Creatinine (µmol L^{-1})	<10	<10
Triglyceride (mmol L^{-1})	1.88±0.00	2.08±0.23
Calcium (mmol L^{-1})	2.75±0.08	2.82±0.03
Phosphate (mmol L^{-1})	3.91±0.36	3.87±0.08
Creatine kinase (U L^{-1})	411±193	451±80
Total bilirubin (mmol L^{-1})	2.90±0.92	4.37±0.57
